# Bifunctional Anti-Non-Amyloid Component α-Synuclein Nanobodies Are Protective *In Situ*

**DOI:** 10.1371/journal.pone.0165964

**Published:** 2016-11-08

**Authors:** David C. Butler, Shubhada N. Joshi, Erwin De Genst, Ankit S. Baghel, Christopher M. Dobson, Anne Messer

**Affiliations:** 1 Neural Stem Cell Institute, Rensselaer, NY, 12144, United States of America; and Department of Biomedical Sciences; University at Albany, Albany, NY, 12208, United States of America; 2 Wadsworth Center, New York State Department of Health, Albany, NY, 12208, United States of America; 3 Department of Chemistry, University of Cambridge, Lensfield Road, Cambridge, CB2 1EW, United Kingdom; Louisiana State University Health Sciences Center, UNITED STATES

## Abstract

Misfolding, abnormal accumulation, and secretion of α-Synuclein (α-Syn) are closely associated with synucleinopathies, including Parkinson’s disease (PD). VH14 is a human single domain intrabody selected against the non-amyloid component (NAC) hydrophobic interaction region of α-Syn, which is critical for initial aggregation. Using neuronal cell lines, we show that as a bifunctional nanobody fused to a proteasome targeting signal, VH14PEST can counteract heterologous proteostatic effects of mutant α-Syn on mutant huntingtin Exon1 and protect against α-Syn toxicity using propidium iodide or Annexin V readouts. We compared this anti-NAC candidate to NbSyn87, which binds to the C-terminus of α-Syn. NbSyn87PEST degrades α-Syn as well or better than VH14PEST. However, while both candidates reduced toxicity, VH14PEST appears more effective in both proteostatic stress and toxicity assays. These results show that the approach of reducing intracellular monomeric targets with novel antibody engineering technology should allow *in vivo* modulation of proteostatic pathologies.

## Introduction

Parkinson’s Disease (PD) is a neurodegenerative disease of aging, characterized neurologically by uncontrolled tremors and bradykinesia due to loss of dopamine-producing neurons in the substantia nigra [1]. Lewy bodies and Lewy neurites, containing misfolded aggregated α-Synuclein (α-Syn), are the most prominent neuropathologic finding [2–4]. Oligomeric, protofibrillar and fibrillar isoforms and multimeric structures can be found both within, and extruded from, affected cells. However, none of these can proceed in the absence of the primary intracellular α-Syn misfolding event, which is therefore an important therapeutic target. The non-amyloid component (NAC) hydrophobic interaction region of α-Syn is critical for aggregation [5], as demonstrated by the absence of aggregation when this region is deleted from the protein [6]. However, this region has been exceptionally difficult to manipulate using traditional immune/antibody or other approaches. We have previously used a human yeast surface display library to select a series of single-chain Fv (scFv) and single domain (nanobody) antibody fragments to determine whether anti-NAC binders could reduce aggregation and protect against the pathogenic effects of overexpressed α-Syn. Our initial candidate, the scFv NAC32, offered modest protection, and initial proof of concept for the target [7]. The strongest binder from the series, an unprotected human VH, was unstable in cytoplasm, requiring further engineering developed for a Huntington’s disease (HD) therapeutics project.

For the aggregating mutant huntingtin exon 1 protein fragment with 72Q repeats, (mhttex1-72Q), we and others have shown that variable antibody (Fv) fragments expressed intracellularly from genes (intrabodies) can significantly reduce aggregation and pathogenic properties of these expanded polyglutamine (polyQ) proteins *in situ* (multiple cell culture models) and *in vivo* (mouse and Drosophila models.) True long-term correction was more limited in the animal models, due to irreversible misfolding during the time that the antigen-antibody complex was dissociated [8–12]. Efficacy is further enhanced with bifunctional constructs that have a proteasomal targeting PEST degron to enhance the degradation during the time that the complex is associated. This degron is directly fused to intrabodies that prevent misfolding of their target antigen [13, 14]. The degron used in this study is from mouse Ornithine Decarboxylase (ODC), a short-lived protein containing a C-terminal PEST degron that has been shown to heterologously reduce the half-life of GFP transcription reporters [15–17].

Fusion with the PEST motif was able to increase cytoplasmic solubility of anti-a-syn fragments (including the unprotected VH14) due to the overall negative charge, while enhancing degradation [14]. In the current study, efficacy of VH14PEST is assessed using a series of in situ models and assays, since each models some but not all aspects of a-syn pathogenesis. These include α-Syn turnover, protection against proteostatic stress in the presence of an additional misfolding Huntingtin (htt) protein, and toxicity measured by multiple criteria. NbSyn87 is a camelid nanobody that binds to residues 118–131 within the C-terminal region of α-Syn. This domain has been identified as the binding site for several existing protective antibodies [18]; therefore we compared two anti-C-terminal nanobodies to the VH14 anti-NAC nanobody. While both offer protection, the NAC region is clearly the more effective target in our assays.

## Methods

### Expression plasmids

Intrabody expression plasmids were labeled with a C-terminal hemagglutinin (HA) epitope tag with amino acid sequence YPYDVPDYA to identify intrabodies. To direct the intrabodies and their cargo to the proteasome, a standard or scrambled PEST motif corresponding to amino acids 422–461 from mouse ODC (GenBank accession number NM_013614.2) was after the HA-tag. NbSyn2, NbSyn87 [19], and VH14 single domain (GenBank Accession number JX430807.1) nanobodies were subcloned with standard cloning techniques into pcDNA3.1- and pAAV-MCS according to the following cloning strategy: XbaI-intrabody-NotI-HA, XbaI-intrabody-NotI-HA-PEST, or XbaI-intrabody-NotI-HA-PEST-Scramble. α-Syn-(Gly_4_Ser)_4_-GFP, α-Syn-(A53T)-(Gly_4_Ser)_4_-GFP, α-Syn, and α-Syn(A53T) were subcloned into pcDNA3.1 at Nhe1 and HindIII restriction sites. Vectors for the expression of human mutant httex1-72Q as previously described were labeled with either GFP (pcDNA3.1-mhttex1-72Q-GFP) [20] or RFP (pcDNA3.1-mhttex1-72Q-RFP) [21]. All expression plasmids were verified by Sanger DNA sequencing and prepared with EndoFree Plasmid Maxi (Qiagen) prep kits according to manufacturer protocol.

### Cell culture and transfection

Undifferentiated ST14A cells, a rat striatal progenitor cell line with high transfectability and many neuronal characteristics (gift from Dr. Elena Cattaneo, Milan IT), were cultured according to standard protocols [22]. 2 μg of total DNA was transfected per well in 6 well plate. JetPEI DNA transfection reagent (Polyplus Transfection Inc.) was used to transiently transfected cells as previously described [23]. For all transfections unless otherwise noted, cells were imaged 48 hours’ post-transfection, and then harvested for downstream applications. For endogenous α-Syn assay, normal SH-SY5Y cells (ATCC) were transiently transfected using Jet PRIME DNA transfection reagents (Polyplus Transfection Inc.), with control (CON; empty vector) or VH14, VH14PEST, or VH14SCRPEST (scrambled inactive PEST) constructs. They were then differentiated into the neuronal pathways 48 hours post- transfection for 3 days with 10 μM retinoic acid (RA) prior to harvesting [24]. To verify that targeted degradation is occurring through the proteasome, specific proteasome inhibitor epoxomicin (10 μM per well) or the vehicle DMSO was applied to the cells 12 h prior to the harvest in a subset of experiments.

### Cell fractionation, Western blotting, and antibodies

48 hours or 5 days after transfection, respectively, transiently transfected ST14A and RA-differentiated SH-SY5Y cells were collected from 6 well culture dishes by trypsinization. Cell pellets were washed in 1x PBS and then lysed in RIPA buffer (50 mM Tris pH 7.5, 150 mM NaCl, 1% NP40, 0.25% sodium deoxycholate, 0.1% SDS) with added 1X Protease Inhibiter Cocktail (Roche). Cell lysates were centrifuged at 1800 rcf to separate soluble and insoluble components. Soluble protein from the supernatant was quantified using Bio-Rad DC Protein Assay kit (Bio-Rad Laboratories). Lysates were adjusted to 1 mg/mL in 2X denaturing sample buffer (125 mM Tris, 4% SDS, 20% glycerol, 10% 2-mercaptoethanol, 0.02% bromophenol blue, pH 6.8) and boiled. 10–15 μg of sample protein was separated by sodium dodecyl sulfate polyacrylamide gel electrophoresis (SDS-PAGE) using 4–20% Criterion Precast Gels (Bio-Rad Laboratories, Inc). A transblot semi-dry (SD) electroblotter (BIORAD) was used to blot the gels on PDVF membranes (Millipore) at 24-27V for 30 min. Membranes were probed for monoclonal α-Syn (anti-syn1; 1:1500; Transduction labs), Actin (anti-actin; 1:2000; Sigma), and HA (anti-HA; 1: 3,000; Covance). Primary antibodies were labeled with goat anti-mouse-HRP (1∶2000, Santa Cruz) secondary antibodies and detected with ECL (PerkinElmer). Exposures were adjusted to be in the linear range of the blot. In some cases, this resulted in minimal detection of the HA labeled band on VH14PEST.

### Aggregation assay

ST14A cells were plated into a 6 well plate. At 24 hours, when they were approximately 70% confluent they were co-transfected with human N terminal huntingtin exon1 protein fragments with 72 polyglutamine repeat length labeled with RFP (httex1-Q72~RFP), either GFP or A53TSyn~GFP, and either empty vector or VH14PEST or VH14SCRPEST. A total of 2 μg of DNA was used per well, in the following fractions: 0.2 μg httex1-Q72~RFP; 0.8 μg of A53T mutant α-Syn~GFP or GFP, and 1 μg empty vector or VH14PEST or VH14SCRPEST. The medium was replaced after four hours of transfection. 48 hours after transfection, the number of RFP-positive (red) foci was counted in 10 random 20X fields per treatment. A 406 lens was used to observe unfixed live cells. A SPOT RT color CCD camera and SPOT advanced software (Diagnostic instruments) were used to capture live cell images 48 hours post transfection. NOTE: While the RFP label is clearly in an aggregated form that matches that previously extensively characterized by us and by others, we are using the term “foci” since we did do a biochemical characterization on these cells.

### Flow cytometry

48 hours post transfection, exhausted media from each well was collected into a 50ml centrifuge tube. The corresponding cells were collected by trypsinization, combined with exhausted media, and then passed through a 70 μm cell strainer (BD Biosciences; removes clumped cells that would clog the tubing), and into a new 50 ml tube. The cells were pelleted by centrifugation (180 rcf×5 minutes) and resuspended in 0.5 ml of FACS buffer/well (~10^6^ cells/ml). To determine viability with Propidium Iodide (PI), cells were pelleted by centrifugation (180 rcf×5 minutes) and resuspended in 0.5ml of FACS buffer (1× Ca^2+^ and Mg^2+^ free PBS, 10% FBS) containing 50 μM PI (Sigma). Labeled cells were sorted by fluorescence using a BD FACSCalibur Flow Cytometer (BD Biosciences) with a minimum of 10,000 events recorded per sample using BD CellQuest Pro software. Data were displayed in two-color dotplot formats on a log-scale, and cell death was expressed as a percentage by dividing the number of cells that stained with PI by the total number of events. Because nanobody-PEST constructs decreased the mean fluorescent intensity (MFI) of GFP, gating for GFP positive cells and PI-positive cells for viability was not possible. To determine the apoptotic role of α-Syn, ST14A cells were transfected as described above, and analyzed for Annexin V expression. Surface expression of phosphatidylserine has been established as an indicator of cellular death associated with apoptosis [25]. Annexin V and 7-Aminoactinomycin D (7AAD) staining of cells was done using R-phycoerythrin-conjugated Annexin V (Annexin V-PE) apoptosis detection kit (BD Pharmingen, San Jose, CA, USA) according to the manufacturers’ instructions. Using BD CellQuest Pro software, data were displayed in two-color dotplot formats on a log-scale. (y-axis = 7AAD, x-axis = annexinV PE). Viable cells are Annexin V negative and 7AAD-negative. Cells in the early phases of cell death are Annexin V positive and 7AAD-negative, and necrotic cells are Annexin V positive and 7AAD-positive. Raw flow cytometry data is shown in S3 Fig and S4 Fig.

### Statistical analysis

All values represent the mean of at least three independent experiments, with multiple individual values per experiment. Proteins were quantified densitometrically and normalized to control (empty vector) and actin. At least 3 independent experiments were performed. Tukey’s pairwise comparison was used to establish the significance of decreased α-Syn with intrabody-PEST and intrabody-SCR-PEST relative to empty vector control, after one-way ANOVA established that the α-Syn level was significantly affected by the presence of intrabody fused with PEST and SCR-PEST motifs (*p<0.05, compared with control). Statistical significance was determined by one-way ANOVA using Minitab or Statview statistical software. P values <0.05 were designated as statistically significant.

## Results

### Bifunctional nanobody-PEST constructs reduce α-Syn levels

**Fig 1:**
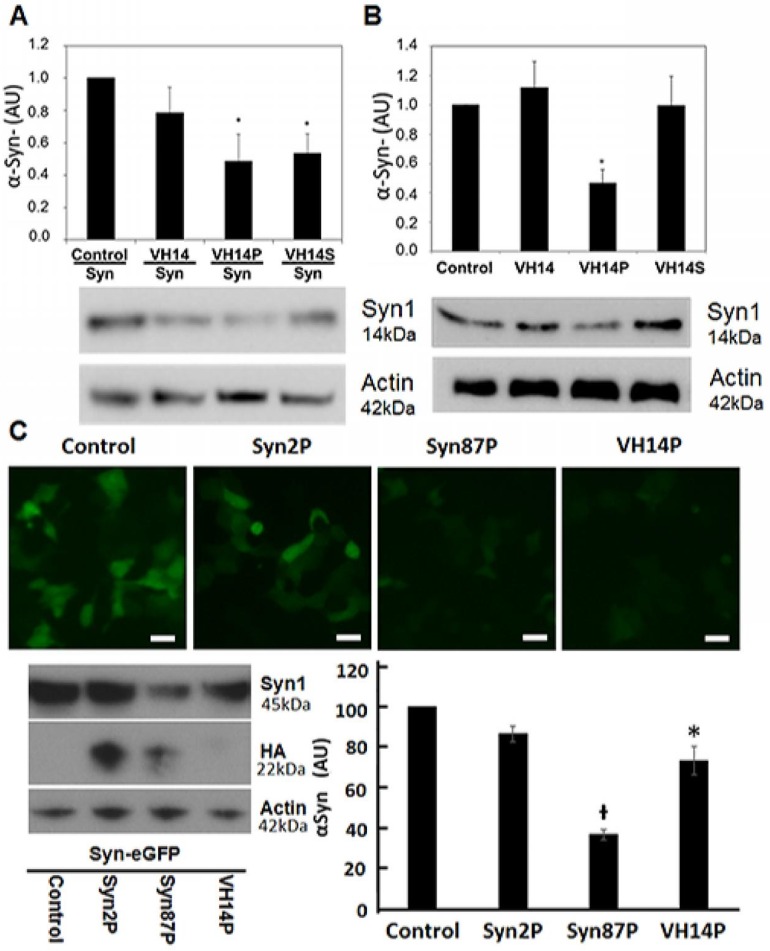
Bifunctional nanobody-PEST reductions of α-Syn levels. (A) Dual transfection of wild type α-Syn plus VH14 constructs in ST14A cells. VH14PEST (VH14P) and VH14 with a scrambled PEST degron (VH14S) significantly reduced (*p<0.05, n = 5) α-Syn based on Western blot densitometry of α-Syn compared with VH14 and empty vector control. (B) Endogenous α-Syn after transfection of VH14PEST constructs in SH-SY5Y cells. Western blot densitometry shows that α-Syn is significantly (*p<0.05, n = 4) reduced by VH14P compared with empty vector control, VH14 and VH14S control (C) Live cell imaging, Western blot, and densitometry for Syn with anti-Syn1. Syn87PEST (Syn87P) significantly reduced empty vector control Syn~GFP protein levels compared to VH14P and Syn2P (Mean ± SEM. *p<0.05 compared to Control and Syn87PEST; ᵻ p<0.05 compared to all groups; n = 3).

Overexpression and endogenous α-Syn expression models were used in two cell lines to mimic the disease pathology that is associated with increased α-Syn levels. Our approach sought to harness the proteasomal machinery for the targeted degradation of α-Syn. To develop bifunctional intracellular nanobodies that redirect α-Syn to the proteasome for degradation, while preventing its misfolding, we fused the mODC-PEST degron (amino-acids 422–461) to the C-terminus of a series of anti-α-Syn nanobodies. To monitor α-Syn expression levels, subcellular localization, and aggregation, we created GFP fusion constructs. Because direct fusion of GFP to the C-terminus of α-Syn results in partial truncation of the fusion protein that spontaneously form inclusions in the neuronal cytoplasm [26], a flexible (Gly_4_Ser)_4_ linker [27], was inserted between GFP and Syn to allow independent folding of the two proteins. In previous studies, VH14PEST was shown to significantly decrease α-Syn~GFP levels [14]. To rule out the possibility that VH14PEST binds to epitopes that are present only in α-Syn~GFP, we tested two additional paradigms. WT α-Syn was co-transfected with VH14 constructs into ST14A cells. Both the intact and the scrambled PEST constructs significantly reduced the amount of WT α-Syn (p< 0.05) (Fig 1A). In addition, nanobody PEST effects on endogenous α-Syn levels were investigated using RA-differentiated SH-SY5Y cells, which show measurable α-Syn. In this case only the intact PEST showed a significant decrease (p<0.05) (Fig 1B).

Next, two anti-C-terminal-α-Syn camelid-derived nanobodies were fused to the PEST degron, allowing comparisons to the anti-NAC VH14. Live cell imaging of co-transfected ST14A cells revealed that Syn~GFP fluorescence was significantly reduced with NbSyn87PEST (Syn87P) and VH14PEST (VH14P) compared to NbSyn2PEST (Syn2P) (Fig 1C). In agreement with live cell imaging, western blotting for α-Syn resulted in a ~64% reduction of α-Syn using Syn87PEST and ~27% using VH14PEST. All nanobodies were labeled with the short HA tag. Anti-HA western blotting for intracellular nanobody levels revealed that VH14PEST protein steady state levels were barely detectable compared to Syn2PEST and Syn87PEST levels (Fig 1C). To verify that NbSyn87PEST is directing α-Syn to the proteasome, we treated cells for 24hrs with 10μm epoxomicin. In agreement with our previously-published studies showing proteasome specificity for VH14PEST [14], protein levels for HA and α-Syn were increased in epoxomicin treated cells compared to vehicle control (S1 Fig).

### VH14PEST can counteract the heterologous proteostatic effects of overexpressed α-Syn on mutant huntingtin Exon1

**Fig 2:**
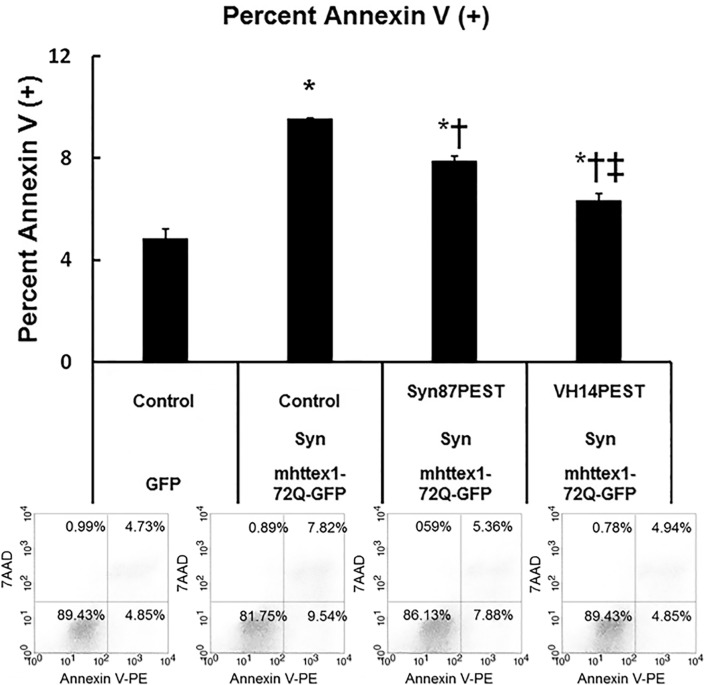
VH14PEST decreases the toxicity of α-Syn as measured by a proteostatic assay with mhttex1-72QGFP. VH14PEST reduced toxicity induced by proteostatic stress of co-expressing mhttex1-72Q-GFP with WT α-Syn overexpression. Overexpression of empty vector control plus mhttex1-72Q-GFP and WT Syn significantly increased early apoptotic cell death compared to empty vector GFP control [Mean ± SEM. *p<0.05 significantly different from all groups; n = 3]. VH14PEST significantly (‡p<0.05) reduced the percentage of apoptotic cell death to a greater extent than Syn87PEST [Mean ± SEM. *p<0.05 compared to Empty Vector control GFP; ‡p<0.05 comparing VH14PEST vs. Syn87PEST; †p<0.05 comparing Syn87PEST vs empty vector control-Syn-mhttex1-72Q-GFP.]. Lower Panel: Representative scatter plots showing the percentage of cells in each quadrant.

Biologically, it is becoming increasingly clear that proteostatic stress due to multiple metastable proteins and limited chaperone activity is an important feature of neurodegenerative diseases [28]. Specifically, α-Syn potentiates the neurotoxicity of mutant huntingtin protein *in vitro* and *in vivo* [29–32]. Our previous studies have shown very robust readouts for HD assays, and somewhat increased aggregation in assays using the A53T mutant form of α-Syn. Therefore, as an initial functional assay in ST14A cells, RFP-labeled mhttex1-72Q was added to overexpressed mutant α-Syn~GFP, providing a visible readout of heterologous proteostatic effects on the mhttex1 aggregation [29–33]. If nanobody PEST reduces α-Syn sufficiently, α-Syn-enhanced accumulation of the mhttex1 should be reduced. ST14A cells were co-transfected with mhttex1-72Q-RFP plus either GFP or the A53T mutant α-Syn~GFP, +/- VH14PEST constructs. With no VH14PEST, there was a significant increase in the RFP-positive htt foci in the presence of A53T α-Syn (S2 Fig). Fluorescent foci were decreased significantly by adding VH14PEST. There was no protective effect of the PEST scrambled fusion construct. The proteostatic model was then expanded to determine if foci correlated with toxicity, and if the C-terminal nanobody construct Syn87PEST was also protective. These experiments were done using overexpressed WT α-Syn to more closely model human PD. Apoptosis was evaluated by flow cytometry with Annexin V. ST14A cells were co-transfected with mhttex1-72Q-GFP, plus α-Syn, and either the anti-Syn nanobodies Syn87PEST, VH14PEST, or GFP. 48h after transfection, live cells were stained with Annexin V and then analyzed by flow cytometry. As expected, early apoptotic death was significantly increased (p<0.05) by the overexpression of multiple potentially aggregating proteins compared to empty vector control (Fig 2). In agreement with S2 Fig, where VH14PEST reduced punctate mhttex1-72Q-RFP, the percentage of cells undergoing early apoptosis was significantly decreased compared to Syn87PEST. However, Syn87PEST did significantly reduce early apoptosis compared to empty vector control GFP alone.

### Nanobodies fused to PEST can protect against direct α-Syn toxicity

**Fig 3:**
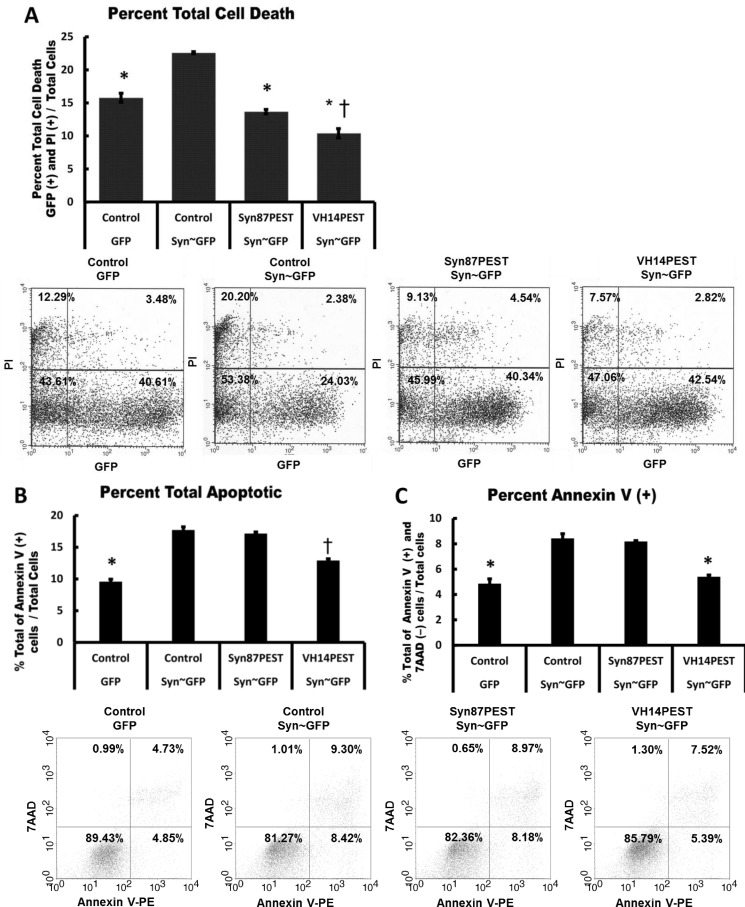
Overexpression of Syn87PEST and VH14PEST reduced total cell death to control levels in ST14A cells. (A) VH14PEST reduced the percentage of total cell death to a greater extent than Syn87PEST. [Mean ± SEM. * p<0.05 compared to Syn-GFP; † p<0.05 compared to Syn87PEST; n = 3]. (B & C) Representative scatter plots showing the percentage of cells in each quadrant. Viable cells are annexin V (−) and 7-AAD (−); annexin V (+) and 7-AAD (−) cells are in early apoptosis; annexin V (+) and 7-AAD (+) cells are in late apoptosis; necrotic cells are annexin V (−) and 7-AAD (+). (B) The percent total of cells undergoing late apoptosis was significantly reduced by VH14PEST compared to empty vector control co-transfected GFP levels. (Mean ± SEM. *p<0.05 comparing control GFP, to other groups; ᵻ p<0.05 compared VH14PEST/SYNGFP to other groups; n = 3). (C) The percent Annexin V positive cells undergoing early apoptosis was significantly reduced to empty vector control co-transfected GFP levels. (Mean ± SEM. *p<0.05 comparing control GFP, VH14PEST Syn-GFP to other groups; n = 3).

To further compare the two nanobodies and their protective effects, parallel experiments were performed using propidium iodide or Annexin V readouts in ST14A cells transfected with α-Syn~GFP or α-SynA53T~GFP. As in the assay above, VH14PEST reduces α-Syn induced toxicity more effectively than Syn87PEST, although there is some protective effect by the latter. To distinguish if changes seen in cell death and viability are due to apoptosis, we assayed the cells with Annexin V and PI. Fig 3A shows that when α-Syn is overexpressed in ST14A cells, Syn87PEST and VH14PEST reduced total cell death, quantified by PI staining, to control levels. Apoptosis was evaluated through Annexin V labeling of transfected ST14A cells. Overexpression of Syn~GFP significantly increased apoptosis compared to control (p<0.05). VH14PEST significantly (p<0.05) reduced total apoptosis (Fig 3B) compared to empty vector control Syn~GFP and Syn87PEST Syn~GFP. VH14PEST significantly reduced (p<0.05) the percentage of Annexin V positive cells (Fig 3C), which correlates to early apoptosis, to empty vector control GFP levels.

## Discussion

When the concentration of α-Syn increases intracellularly, this elevated concentration can lead to misfoding, and formation of toxic α-Syn oligomers, protofibrils, and fibrils [34]. However, monomeric α-Syn is thought to be unstructured when initially synthesized. Bi-functional intracellular nanobodies that can inhibit misfolding, and reduce the concentrations of α-Syn monomers, offer an appealing therapeutic approach. This study compares two specific individual candidate nanobodies in depth. VH14 is a human heavy chain only construct selected against the NAC hydrophobic interaction region of α-Syn, with very low solubility in the absence of fusions that alter the overall charge. NbSyn87 is a camelid nanobody that binds to α-Syn residues 118–130 [19]; it is also heavy chain only, but as a camelid VHH appears to possess the properties of higher solubility, thermal stability, and refolding capacity than the unprotected human VH [35, 36]. However, while the nanobody that binds to the C-terminal region of α-Syn clearly can reduce the intracellular levels of α-Syn, and has some beneficial effects on both proteostatic stress and cellular toxicity, it appears that the protective effects of the solubilized VH14PEST fusion protein are somewhat stronger.

The proteasomal targeting signal has been fused to antibody variable fragments in order to direct the antigen-antibody complexes to the proteasome for clearance. Such fusions also impart a strong negative charge on the protein, which improves intracellular solubility [14, 37]. To date, this has primarily been tested in systems where the targets mhttex1-72Q protein fragments [13] or α-Syn [14] have been labeled with fluorochromes to allow live cell imaging. However, such labeling can potentially perturb the system; therefore, a set of experiments verifying efficacy in the absence of label and using endogenous cellular levels of α-Syn were performed. Both overexpressed WT α-Syn and endogenous α-Syn showed enhanced turnover in VH14PEST transfected cells. When WT α-Syn was overexpressed in dual transfections of ST14A cells, both the intact and the scrambled PEST constructs significantly reduced the α-Syn. On the other hand, using the RA-differentiated SH-SY5Y neuronal cell line, only the functionally complete PEST nanobody was effective. The transfection efficiency of the different constructs was similar when transfected into ST14A cells; therefore, this does not appear to be the reason for the differential effectiveness of the scrambled PEST sequence (data not shown). In the dual transient ST14A transfection paradigm, levels of α-Syn would be higher than those produced endogenously in the differentiated SH-SY5Y cell line. The higher load of α-Syn may be degraded by both lysosomes and proteasomes in the ST14A cell line, but may be primarily via proteasomes in the endogenous case. Also, the scrambled PEST sequence may have retained some capacity to direct the fusion proteins to the proteasome, which again might be a stronger contributor where the α-Syn clearance capacity needs to be greater.

A range of full-length and fragment antibody studies have suggested that the C-terminal region might be a very effective target, although some of this work appeared to target primarily extracellular species [38]. Two anti-C-terminal nanobodies have been studied extensively [19, 39, 40]. These two nanobodies were therefore compared to VH14 for turnover and protective efficacy of their PEST fusions. A GFP labeled α-Syn was used to allow live cell imaging and was followed by quantitation by western blotting. The α-Syn clearance capacity was Syn87>VH14>>Syn2. Syn2, binds to the extreme C terminus (residues 136–140) and buries the C-terminal carboxylic group of Ala 140 in a deep pocket on the surface of the nanobody [39, 40]. Therefore, Syn2 was most likely unable to bind to the α-Syn~GFP protein, despite the use of the flexible (Gly_4_Ser)_4_ linker between the α-Syn and the GFP, and was not continued in our analyses.

Proteostatic stress assays allowed evaluation of an important aspect of functional protection, particularly in aging neurons. Recent reports have shown that α-Syn potentiates the neurotoxicity of mutant huntingtin protein *in vitro* and *in vivo* [29–32]. Mutant httex1-72Q protein fragments have been shown to form cytoplasmic aggregates and toxicity *in vitro* in the ST14A cells [13]. In R6/2 and httQ150 transgenic mice brains, with the progression of the disease, the aggregates were shown to become bigger, and functional deficits appeared after the presence of the aggregates [41, 42]. These data indicated the aggregating mhttex1-72Q, as seen in structures referred to here as foci, may be the cause of cellular toxicity in our model. Proteasome targeting of intrabodies was initially developed for HD by Butler et al. [13] using the ST14 cell model transiently transfected the mhttex1-72Q-GFP plus the huntingtin specific scFv intrabody anti-htt N17 C4-PEST to demonstrate reduction of cellular toxicity in a flow cytometry assay. Recently, in the models using transfected neurons and *in vivo* in HD mice, Tomas-Zapico [31] identified colocalization of microaggregates of α-Syn and N-terminal mutant htt (N-mut-htt) in the brains of R6/1 and HD94 inducible mice expressing α-Syn. Expression of N-mut-htt increased the number of α-Syn filaments, while knocking out α-Syn decreased the number of N-mut-htt aggregates. In HD mice, the reduction of body weight and early HD related motor symptoms were alleviated with α-Syn knockout [30]. This indicated that α-Syn can alter the PolyQ toxicity, and that it is possible to use the reduction in the number of htt foci as a surrogate measure of reduction of toxicity by VH14PEST. The number of RFP positive htt foci in the presence of mutant A53T-Syn was significantly reduced by VH14PEST (S2 Fig). It is possible that polyQ expression may overwhelm the cellular folding machinery and α-Syn may add to the stress [31]. It is also possible that these two proteins may be competing for the lysosomal degrading machinery (Ravikumar [30, 43–45]. Our bifunctional intrabody approach for degrading the intracellular α-Syn might also have potential as a future HD therapy, or for other neurodegenerative diseases that are recognized as participants in an interacting proteostatic stress network [28].

Critical questions for design and delivery of nanobodies as anti-PD therapeutics include which conformation(s) of α-Syn are contributing to pathogenesis, which epitopes are available on this conformer at critical stages of the process, and where within or outside the cells these targets can be localized. VH14 would be expected to bind primarily to the monomeric form of α-Syn, since the NAC region may be rapidly bound in homo or heterologous multimers, with the epitope buried internally and inaccessible to the nanobody [7]. Clearance of higher order structures of α-Syn have been shown to require autophagy [46]. A recent publication showed that relative concentrations of the different forms of α-Syn, including extracellular forms of the monomer, may play important roles in PD [47]. It was especially interesting that when the monomeric form was added exogenously to cells, it could trigger aggregation of the endogenous α-Syn, leading to apoptosis. Furthermore, reduction in the levels of monomer (in this case by fibrillar seeding) was protective [47].

In previous studies, we have also seen some anti-α-Syn effects with a single-chain Fv clone from an NAC binding selection, although it appeared to have lower intracellular stability than the engineered VH14PEST nanobody used here [7]. In an effort to identify additional stable nanobodies that had undergone *in vivo* maturation, we immunized an alpaca using a combination of full-length α-Syn and AA53-87-KLH peptides. (Collaboration with Dr. Charles Shoemaker, Tufts Cummings School of Veterinary Medicine) The resultant phage display library was screened against the immunizing NAC peptide and ten intact clones were selected for further analysis. None of these showed stronger effects than VH14 (data not shown); therefore, the human clone was continued as our lead anti-NAC candidate. If, moving forward, it appears that a more pan-specific anti- α-Syn intrabody that recognizes both monomers and oligomers would be useful (alone or in combination), we can pursue further work with the scFv-D10 construct that showed some anti- α-Syn efficacy in HEK293 non-neuronal cell assays, despite some instability [48].

There could be several explanations for the observations that the nanobody against the C-terminal cleared α-Syn more efficiently, but was less effective at protection against toxicity. We favor the hypothesis that clearance beyond the minimal effective level may actually induce an additional cellular stressor. The differences between the two clones tested in the current studies were modest, and may be specific to the individual constructs tested, rather than the specific epitopes. Differences could be a function of relative steady-state levels that are the end product of efficiency of translation/ initial folding in combination with degradation of the fusion with PEST. Higher expression levels of intrabodies are not necessarily superior, since they have the potential to themselves require additional chaperone activity which would increase proteostatic stress, although camelid nanobodies are frequently more soluble intracellularly than unprotected human or mouse single-domain antibodies [35]. These two individual constructs also may have different affinities for their targets in the complex milieu of the cellular cytoplasm, which is difficult to quantitate functionally. In this therapeutic paradigm, modest affinity that allows dissociation at the head of the proteasome may be more effective than a very strong binder where the entire antigen-antibody complex will enter the organelle and be cleared.

The results identify nanobody candidates to move forward for *in vivo* testing. Given that both constructs did offer a significant level of protection, pilot testing of both would be worthwhile in a rapid animal model. Protecting even a modest percentage of cells in this way could reduce cell-to-cell spread in the brain. A direct application of this *in situ* work is that stable nanobody production in healthy stem cells that will be used for transplants may also protect them from exogenous misfolded α –Syn or other prion-like proteins that depend upon high intracellular concentrations of monmeric forms of the proteins. Such delivery could be *ex vivo*. As a more ubiquitously applicable antibody engineering technology, fusion of antibody fragments against specific targets with degron polypeptides offers the option to knock down intracellular proteins at the translational and post-translational levels for functional therapeutic and mechanistic studies.

## Conclusions

The studies reported in this paper clearly support the efficacy of nanobody reagents that target the hydrophobic interaction region of α-Syn for PD as well as other synucleinopathies such as dementia with Lewy bodies, and multiple system atrophy. Although most anti-PD studies to date have found that therapeutically active antibodies derived from immunizations with α-Syn bind to the C-terminal regions, some of this effect could be due to enhanced immunogenicity of epitopes from this region. By screening a non-immune human yeast surface display library against specific internal peptides, we were able to select clones that targeted domains that are less immunogenic, and/ or more transiently available to the immune system. Our data highlight the importance of evaluating multiple aspects of candidate nanobodies on a case by case basis. *In vivo* testing of both constructs will be very informative.

## Supporting Information

S1 Fig Proteasome Inhibition. 24 hour treatment with 10μM proteasome inhibitor epoxomicin revealed that Syn87PEST degrades α-Syn through the proteasome. Proteasome inhibition of Syn87PEST-Syn~GFP co-transfected cells resulted in an increase of anti-α-Syn (Syn1) protein levels. Syn87PEST nanobody protein levels measured by HA were increased proteasome inhibition.
(TIF)

S2 Fig VH14PEST decreases the toxicity of α-Syn as measured by a proteostatic assay with mhttex1-72QRFP. RFP-positive foci (Inset) were counted under the fluorescence microscope in 20X fields. At least 10 random 20X fields were counted per treatment. The number of foci was significantly affected by the presence of nanobody fused with PEST (*** p < 0.001, comparisons between GFP + Control and A53T~GFP + Control; A53T~GFP + Control and A53T~GFP + VH14PEST; n = 3). Residuals were checked for normality.
(TIF)

S3 Fig Flow cytometry raw data files for Figs 2 and 3. (PDF)

S4 Fig Flow cytometry raw data files for Fig 3. (PDF)
