# Supplementary figures and images for: Bifunctional Anti-Non-Amyloid Component α-Synuclein Nanobodies Are Protective In Situ
Source: PLoS One. 2016 Nov 8;11(11):e0165964. doi: 10.1371/journal.pone.0165964 (PMC5100967; doi:10.1371/journal.pone.0165964)

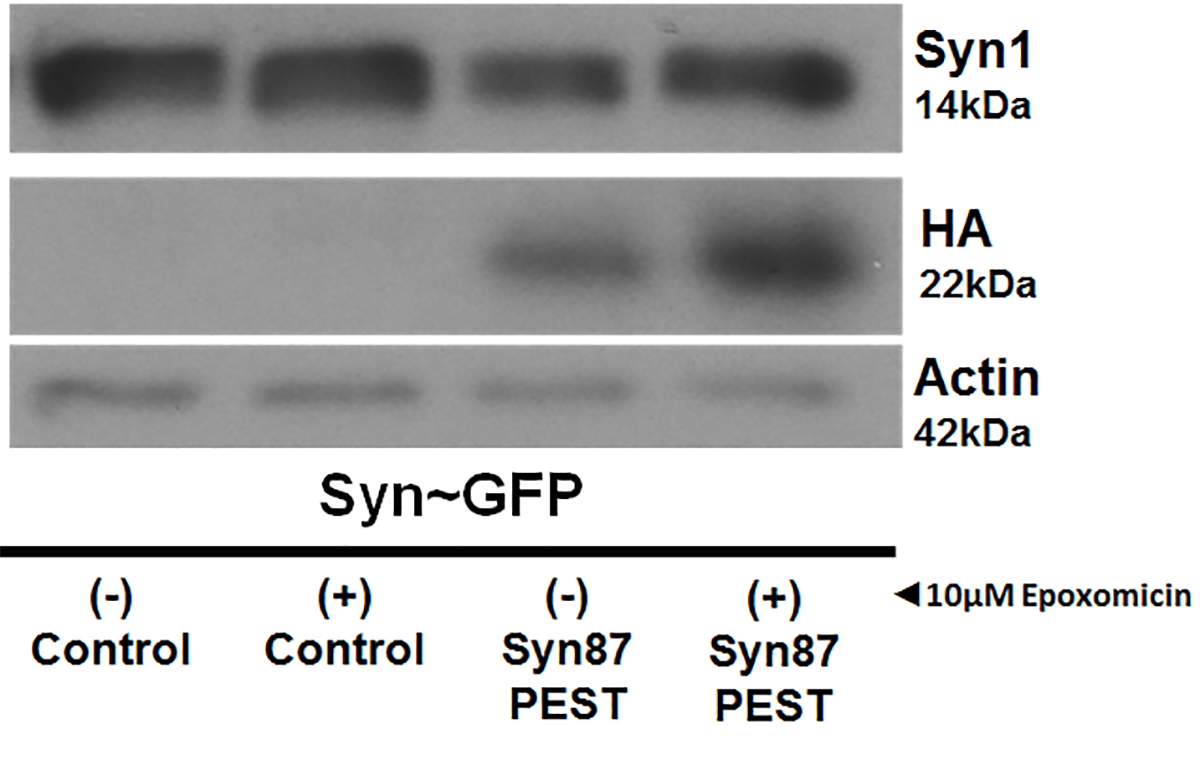

Supplement: S1 Fig — 24 hour treatment with 10μM proteasome inhibitor epoxomicin revealed that Syn87PEST degrades α-Syn through the proteasome. Proteasome inhibition of Syn87PEST-Syn~GFP co-transfected cells resulted in an increase of anti-α-Syn (Syn1) protein levels. Syn87PEST nanobody protein levels measured by HA were increased proteasome inhibition. (TIF) [file pone.0165964.s001.tif]

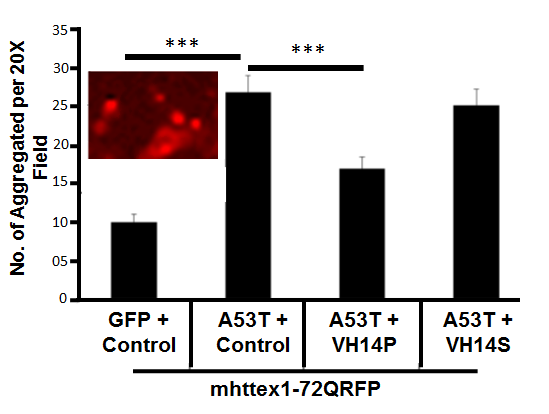

Supplement: S2 Fig — RFP-positive foci (Inset) were counted under the fluorescence microscope in 20X fields. At least 10 random 20X fields were counted per treatment. The number of foci was significantly affected by the presence of nanobody fused with PEST (*** p < 0.001, comparisons between GFP + Control and A53T~GFP + Control; A53T~GFP + Control and A53T~GFP + VH14PEST; n = 3). Residuals were checked for normality. (TIF) [file pone.0165964.s002.tif]
