# Supplementary material for: Bifunctional Anti-Non-Amyloid Component α-Synuclein Nanobodies Are Protective In Situ
Source: PLoS One. 2016 Nov 8;11(11):e0165964. doi: 10.1371/journal.pone.0165964 (PMC5100967; doi:10.1371/journal.pone.0165964)
Supplement: S3 Fig — (PDF) [file pone.0165964.s003.pdf]

Sample #	Treatment
9	ST14A Transfection control
10	ST14A Transfection control
11	ST14A Transfection control
12	Empty Vector Control-pAAV/ GFP-pcDNA3.1-
13	Empty Vector Control-pAAV/ GFP-pcDNA3.1--
14	Empty Vector Control-pAAV/ GFP-pcDNA3.1-
18	Empty Vector Control-pAAV /Syn~GFP-pcDNA3.1-
19	Empty Vector Control-pAAV /Syn~GFP-pcDNA3.1-
20	Empty Vector Control-pAAV /Syn~GFP-pcDNA3.1-
21	Syn87PEST-pAAV /Syn~GFP-pcDNA3.1-
22	Syn87PEST-pAAV /Syn~GFP-pcDNA3.1-
23	Syn87PEST-pAAV /Syn~GFP-pcDNA3.1-
24	Vh14PEST-pAAV / Syn~GFP-pcDNA3.1-
25	Vh14PEST-pAAV / Syn~GFP-pcDNA3.1-
26	Vh14PEST-pAAV / Syn~GFP-pcDNA3.1-
27	Empty Vector Control-pAAV /Syn-pcDNA3.1-/mhttex1-72QGFP-pcDNA3.1-
28	Empty Vector Control-pAAV /Syn-pcDNA3.1-/mhttex1-72QGFP-pcDNA3.1-
29	Empty Vector Control-pAAV /Syn-pcDNA3.1-/mhttex1-72QGFP-pcDNA3.1-
30	Syn87PEST-pAAV /Syn-pcDNA3.1-/mhttex1-72QGFP-pcDNA3.1-
31	Syn87PEST-pAAV /Syn-pcDNA3.1-/mhttex1-72QGFP-pcDNA3.1-
32	Syn87PEST-pAAV /Syn-pcDNA3.1-/mhttex1-72QGFP-pcDNA3.1-
33	Vh14PEST-pAAV /Syn-pcDNA3.1-/mhttex1-72QGFP-pcDNA3.1-
34	Vh14PEST-pAAV /Syn-pcDNA3.1-/mhttex1-72QGFP-pcDNA3.1-
35	Vh14PEST-pAAV /Syn-pcDNA3.1-/mhttex1-72QGFP-pcDNA3.1-

ST14A

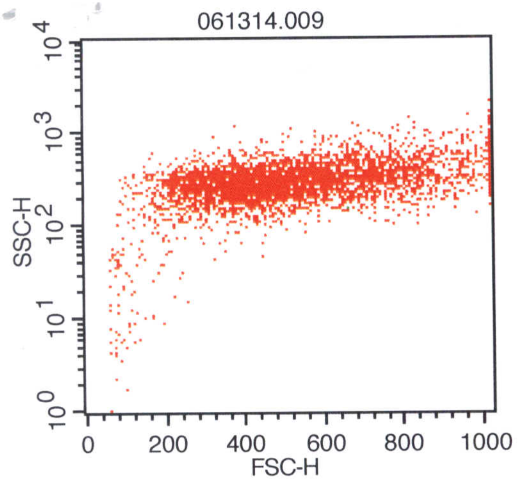

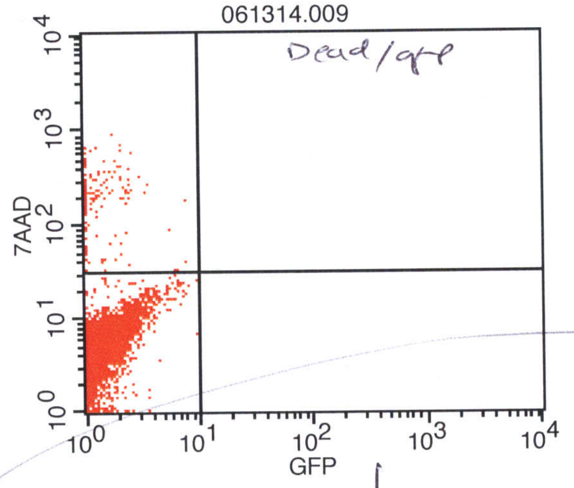

File: 061314.009
Sample ID:
Acquisition Date: 13-Jun-14
Gated Events: 20000
X Parameter: FSC-H (Linear)

Region	Events	% Gated	%
R2	2	0.01	

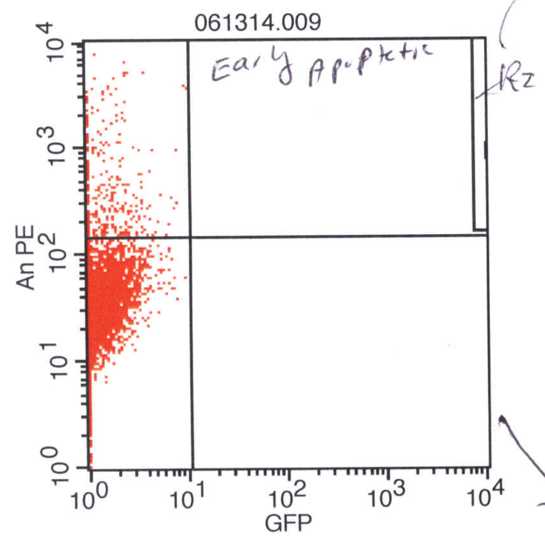

File: 061314.009
Sample ID:
Acquisition Date: 13-Jun-14
Gated Events: 19998
X Parameter: GFP (Log)
Quad Location: 10, 30

Log Data Units: Linear Values
Patient ID:
Gate: G1
Total Events: 20000
Y Parameter: 7AAD (Log)

Quad	Events	% Gated	% Total	X Mean	X Geo Mean	Y Mean	Y Geo Mean
UL	771	3.86	3.85	1.61	1.40	255.84	196.06
UR	16	0.08	0.08	22.01	17.97	135.48	70.49
LL	19205	96.03	96.03	1.53	1.41	5.66	4.70
LR	6	0.03	0.03	616.36	60.16	10.15	4.51

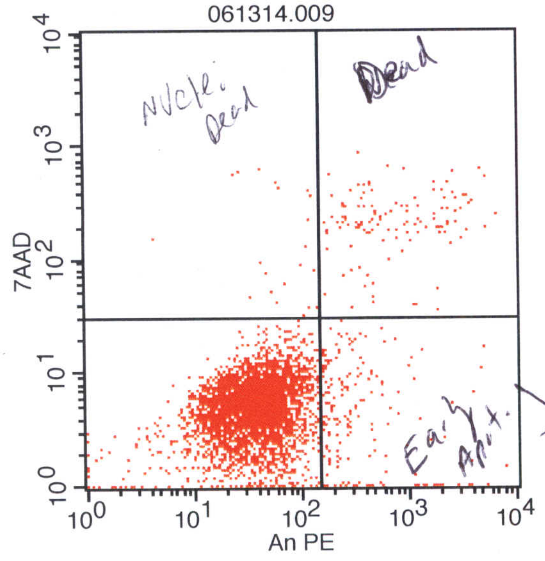

File: 061314.009
Sample ID:
Acquisition Date: 13-Jun-14
Gated Events: 19998
X Parameter: GFP (Log)
Quad Location: 11, 145

Log Data Units: Linear Values
Patient ID:
Gate: G1
Total Events: 20000
Y Parameter: An PE (Log)

Quad	Events	% Gated	% Total	X Mean	X Geo Mean	Y Mean	Y Geo Mean
UL	1533	7.67	7.66	1.88	1.62	879.82	477.00
UR	10	0.05	0.05	27.91	22.83	1094.34	708.58
LL	18446	92.24	92.23	1.50	1.40	42.12	33.93
LR	9	0.05	0.04	415.65	37.33	57.64	35.05

File: 061314.009
Sample ID:
Acquisition Date: 13-Jun-14
Gated Events: 19998
X Parameter: An PE (Log)
Quad Location: 149, 30

Log Data Units: Linear Values
Patient ID:
Gate: G1
Total Events: 20000
Y Parameter: 7AAD (Log)

Quad	Events	% Gated	% Total	X Mean	X Geo Mean	Y Mean	Y Geo Mean
UL	137	0.69	0.69	83.17	68.66	197.92	128.87
UR	647	3.24	3.23	1280.75	786.10	266.18	210.75
LL	18359	91.80	91.80	42.06	33.86	5.60	4.72
LR	855	4.28	4.28	614.08	347.43	6.93	4.38

ST14A

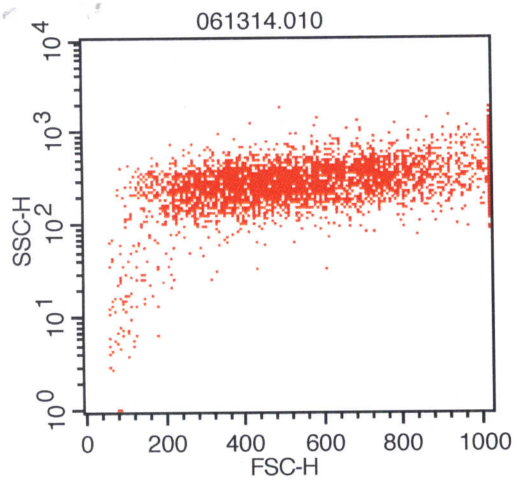

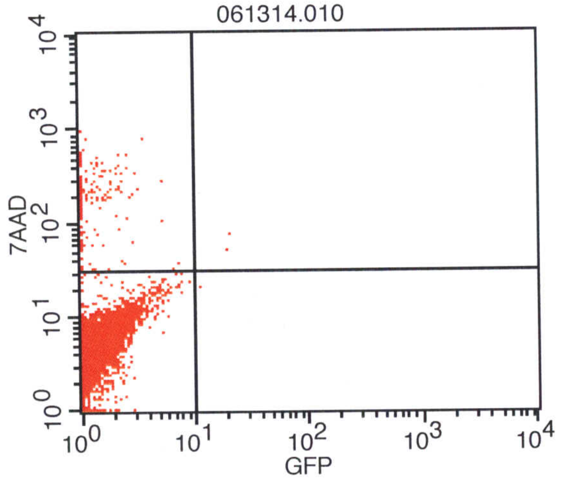

File: 061314.010
Sample ID:
Acquisition Date: 13-Jun-14
Gated Events: 20000
X Parameter: FSC-H (Linear)

Region	Events	% Gated	%
R2	0	0.00	

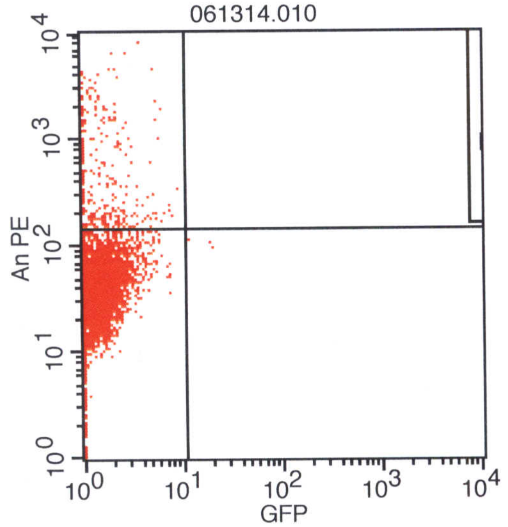

File: 061314.010
Sample ID:
Acquisition Date: 13-Jun-14
Gated Events: 20000
X Parameter: GFP (Log)
Quad Location: 10, 30

Log Data Units: Linear Values
Patient ID:
Gate: G1
Total Events: 20000
Y Parameter: 7AAD (Log)

Quad	Events	% Gated	% Total	X Mean	X Geo Mean	Y Mean	Y Geo Mean
UL	751	3.75	3.75	1.69	1.43	239.80	180.13
UR	19	0.10	0.10	59.71	22.55	220.25	87.25
LL	19223	96.11	96.11	1.53	1.41	5.79	4.80
LR	7	0.03	0.03	12.43	12.27	23.73	22.58

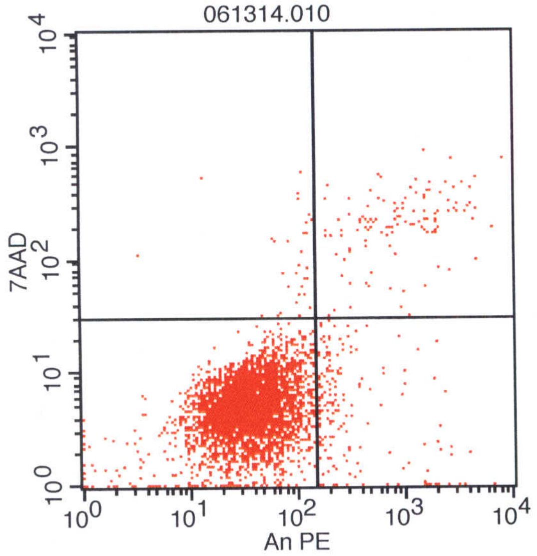

File: 061314.010
Sample ID:
Acquisition Date: 13-Jun-14
Gated Events: 20000
X Parameter: GFP (Log)
Quad Location: 11, 145

Log Data Units: Linear Values
Patient ID:
Gate: G1
Total Events: 20000
Y Parameter: An PE (Log)

Quad	Events	% Gated	% Total	X Mean	X Geo Mean	Y Mean	Y Geo Mean
UL	1547	7.74	7.74	1.96	1.66	915.32	485.34
UR	9	0.04	0.04	105.57	32.75	893.16	462.77
LL	18432	92.16	92.16	1.50	1.39	42.96	34.73
LR	12	0.06	0.06	18.31	16.55	100.29	96.76

File: 061314.010
Sample ID:
Acquisition Date: 13-Jun-14
Gated Events: 20000
X Parameter: An PE (Log)
Quad Location: 149, 30

Log Data Units: Linear Values
Patient ID:
Gate: G1
Total Events: 20000
Y Parameter: 7AAD (Log)

Quad	Events	% Gated	% Total	X Mean	X Geo Mean	Y Mean	Y Geo Mean
UL	127	0.64	0.64	78.34	58.00	179.76	112.05
UR	638	3.19	3.19	1411.17	881.40	252.81	196.49
LL	18368	91.84	91.84	43.04	34.77	5.72	4.80
LR	867	4.33	4.33	595.40	335.45	7.60	4.92

STIYA

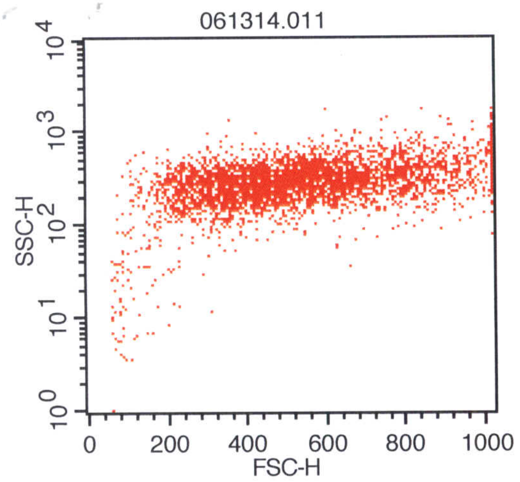

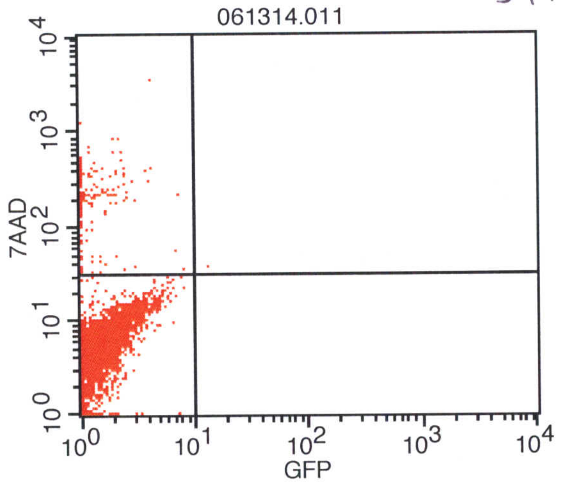

File: 061314.011
Sample ID:
Acquisition Date: 13-Jun-14
Gated Events: 20000
X Parameter: FSC-H (Linear)

Region	Events	% Gated	%
R2	0	0.00	

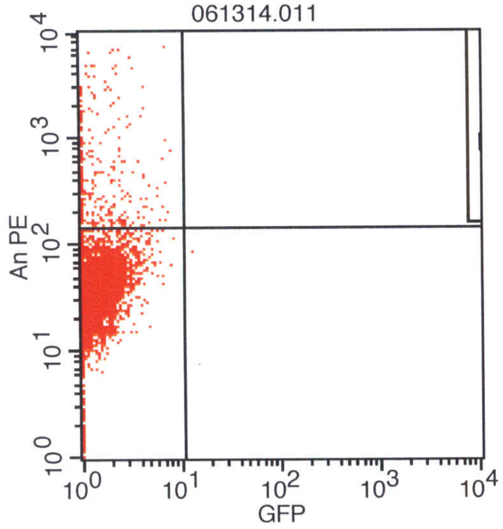

File: 061314.011
Sample ID:
Acquisition Date: 13-Jun-14
Gated Events: 20000
X Parameter: GFP (Log)
Quad Location: 10, 30

Log Data Units: Linear Values
Patient ID:
Gate: G1
Total Events: 20000
Y Parameter: 7AAD (Log)

Quad	Events	% Gated	% Total	X Mean	X Geo Mean	Y Mean	Y Geo Mean
UL	810	4.05	4.05	1.59	1.39	256.00	193.71
UR	16	0.08	0.08	26.10	17.63	95.42	63.46
LL	19171	95.86	95.86	1.53	1.42	5.75	4.80
LR	3	0.01	0.01	11.49	11.48	27.49	27.47

File: 061314.011
Sample ID:
Acquisition Date: 13-Jun-14
Gated Events: 20000
X Parameter: GFP (Log)
Quad Location: 11, 145

Log Data Units: Linear Values
Patient ID:
Gate: G1
Total Events: 20000
Y Parameter: An PE (Log)

Quad	Events	% Gated	% Total	X Mean	X Geo Mean	Y Mean	Y Geo Mean
UL	1499	7.50	7.50	1.84	1.59	845.11	447.37
UR	11	0.06	0.06	32.30	20.72	953.86	573.49
LL	18482	92.41	92.41	1.51	1.40	42.45	34.38
LR	8	0.04	0.04	12.10	12.02	78.61	77.74

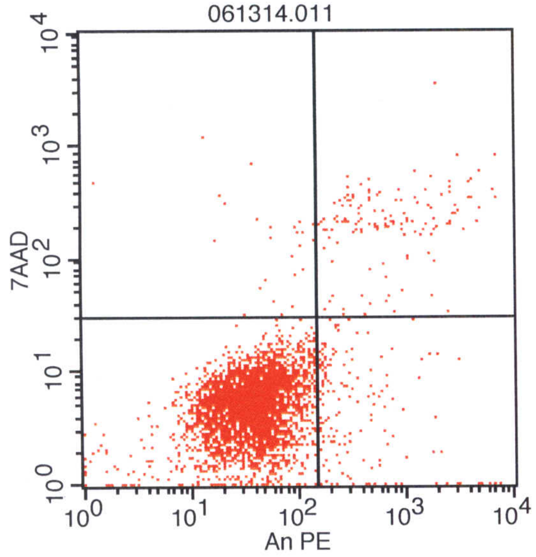

File: 061314.011
Sample ID:
Acquisition Date: 13-Jun-14
Gated Events: 20000
X Parameter: An PE (Log)
Quad Location: 149, 30

Log Data Units: Linear Values
Patient ID:
Gate: G1
Total Events: 20000
Y Parameter: 7AAD (Log)

Quad	Events	% Gated	% Total	X Mean	X Geo Mean	Y Mean	Y Geo Mean
UL	165	0.83	0.83	80.90	65.92	168.11	113.56
UR	660	3.30	3.30	1240.33	713.88	274.42	216.08
LL	18378	91.89	91.89	42.43	34.33	5.69	4.80
LR	797	3.98	3.98	565.72	328.22	7.22	4.70

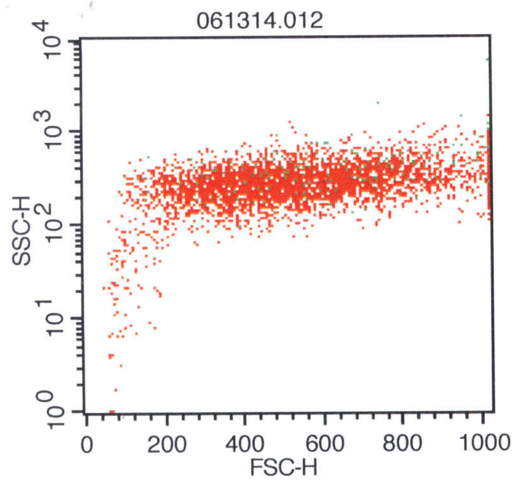

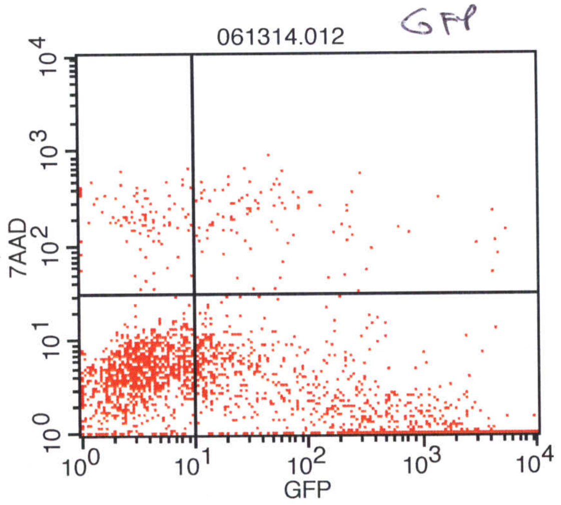

File: 061314.012
Sample ID:
Acquisition Date: 13-Jun-14
Gated Events: 20000
X Parameter: FSC-H (Linear)

Region	Events	% Gated	%
R2	1395	6.98	

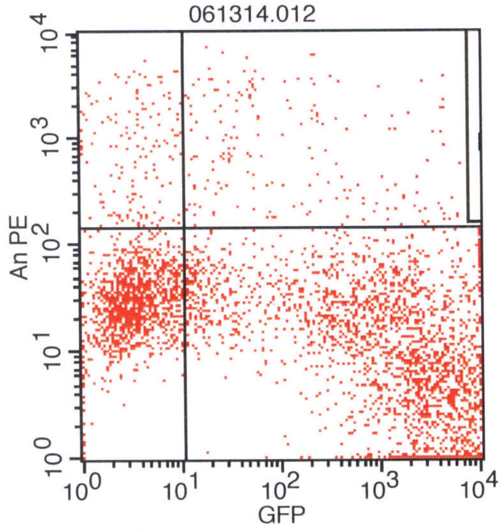

File: 061314.012
Sample ID:
Acquisition Date: 13-Jun-14
Gated Events: 18605
X Parameter: GFP (Log)
Quad Location: 10, 30

Log Data Units: Linear Values
Patient ID:
Gate: G1
Total Events: 20000
Y Parameter: 7AAD (Log)

Quad	Events	% Gated	% Total	X Mean	X Geo Mean	Y Mean	Y Geo Mean
UL	519	2.79	2.60	4.32	3.63	242.39	195.71
UR	501	2.69	2.50	329.00	56.58	219.65	166.97
LL	5822	31.29	29.11	3.89	3.32	5.04	4.18
LR	11763	63.22	58.81	2694.97	945.92	1.96	1.38

File: 061314.012
Sample ID:
Acquisition Date: 13-Jun-14
Gated Events: 18605
X Parameter: GFP (Log)
Quad Location: 11, 145

Log Data Units: Linear Values
Patient ID:
Gate: G1
Total Events: 20000
Y Parameter: An PE (Log)

Quad	Events	% Gated	% Total	X Mean	X Geo Mean	Y Mean	Y Geo Mean
UL	833	4.48	4.17	4.39	3.66	1224.38	737.15
UR	915	4.92	4.58	1008.21	138.64	1313.49	729.38
LL	5648	30.36	28.24	4.02	3.39	35.24	27.87
LR	11209	60.25	56.05	2760.44	1032.22	21.15	9.53

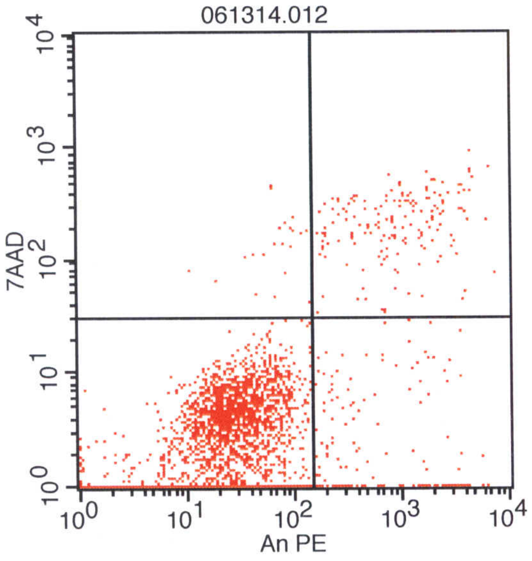

File: 061314.012
Sample ID:
Acquisition Date: 13-Jun-14
Gated Events: 18605
X Parameter: An PE (Log)
Quad Location: 149, 30

Log Data Units: Linear Values
Patient ID:
Gate: G1
Total Events: 20000
Y Parameter: 7AAD (Log)

Quad	Events	% Gated	% Total	X Mean	X Geo Mean	Y Mean	Y Geo Mean
UL	139	0.75	0.69	77.86	61.53	177.43	126.06
UR	880	4.73	4.40	1355.41	858.46	239.95	192.06
LL	16751	90.03	83.75	25.68	13.55	2.91	1.98
LR	835	4.49	4.17	1226.50	661.35	4.45	2.39

G+P

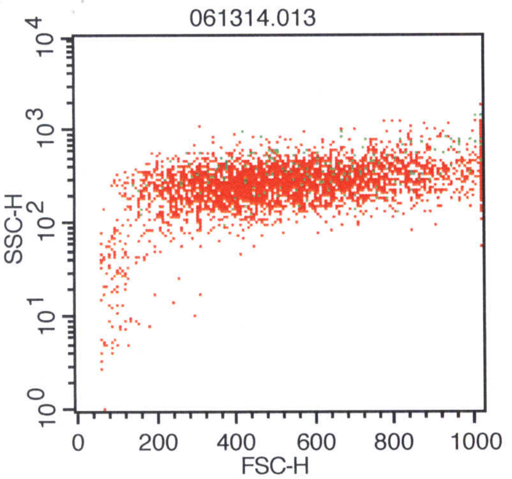

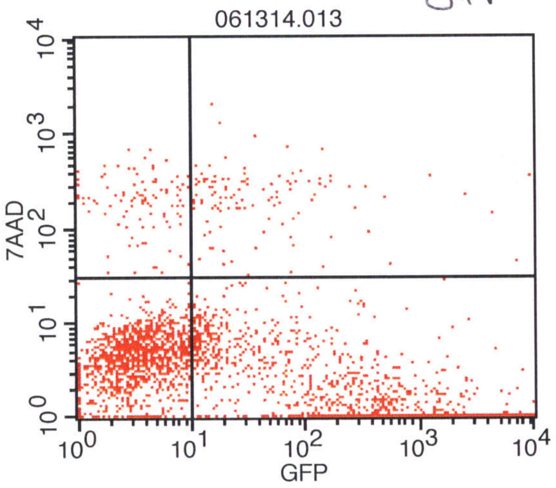

File: 061314.013
Sample ID:
Acquisition Date: 13-Jun-14
Gated Events: 20000
X Parameter: FSC-H (Linear)

Region	Events	% Gated	%
R2	1461	7.31	

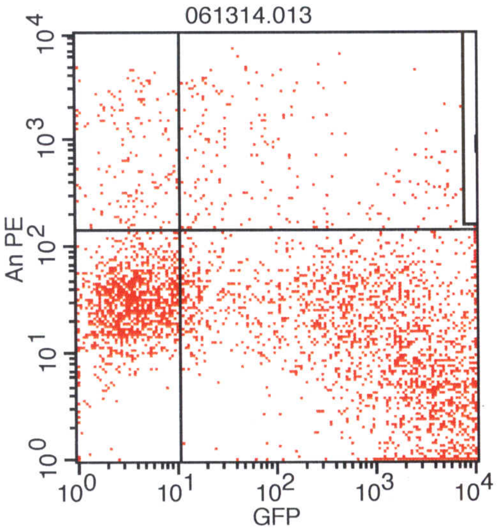

File: 061314.013
Sample ID:
Acquisition Date: 13-Jun-14
Gated Events: 18539
X Parameter: GFP (Log)
Quad Location: 10, 30

Log Data Units: Linear Values
Patient ID:
Gate: G1
Total Events: 20000
Y Parameter: 7AAD (Log)

Quad	Events	% Gated	% Total	X Mean	X Geo Mean	Y Mean	Y Geo Mean
UL	549	2.96	2.74	4.44	3.69	257.24	198.51
UR	532	2.87	2.66	250.43	47.19	231.49	171.55
LL	5682	30.65	28.41	3.99	3.39	5.06	4.19
LR	11776	63.52	58.88	2659.62	906.78	2.05	1.42

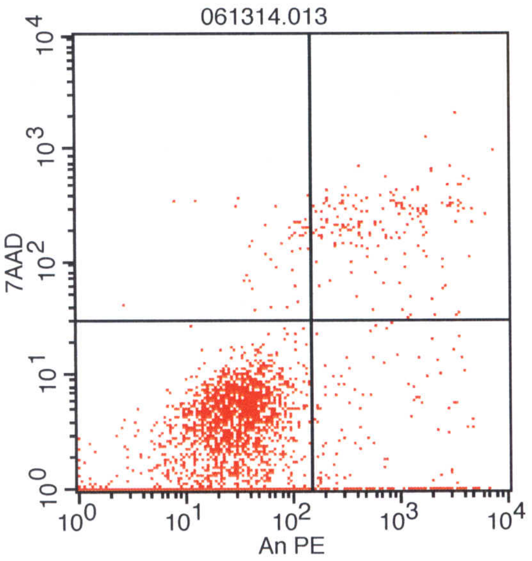

File: 061314.013
Sample ID:
Acquisition Date: 13-Jun-14
Gated Events: 18539
X Parameter: GFP (Log)
Quad Location: 11, 145

Log Data Units: Linear Values
Patient ID:
Gate: G1
Total Events: 20000
Y Parameter: An PE (Log)

Quad	Events	% Gated	% Total	X Mean	X Geo Mean	Y Mean	Y Geo Mean
UL	822	4.43	4.11	4.52	3.75	1244.52	743.04
UR	1124	6.06	5.62	1166.43	177.10	1163.19	669.06
LL	5575	30.07	27.88	4.14	3.48	36.22	28.29
LR	11018	59.43	55.09	2735.54	993.53	22.81	10.38

File: 061314.013
Sample ID:
Acquisition Date: 13-Jun-14
Gated Events: 18539
X Parameter: An PE (Log)
Quad Location: 149, 30

Log Data Units: Linear Values
Patient ID:
Gate: G1
Total Events: 20000
Y Parameter: 7AAD (Log)

Quad	Events	% Gated	% Total	X Mean	X Geo Mean	Y Mean	Y Geo Mean
UL	203	1.09	1.01	79.76	64.56	181.02	136.10
UR	877	4.73	4.38	1275.17	818.45	259.52	198.70
LL	16420	88.57	82.10	26.88	14.33	2.96	2.01
LR	1039	5.60	5.20	1162.36	640.65	4.25	2.19

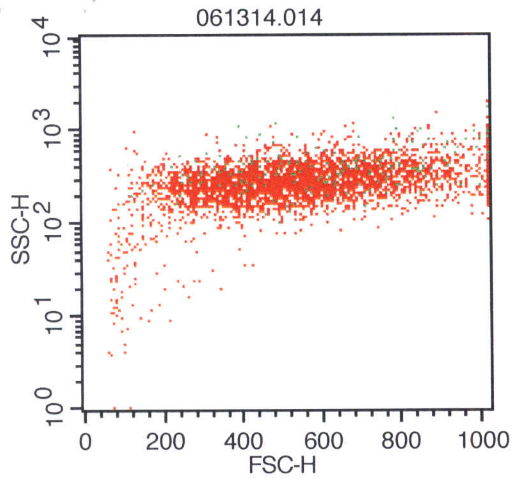

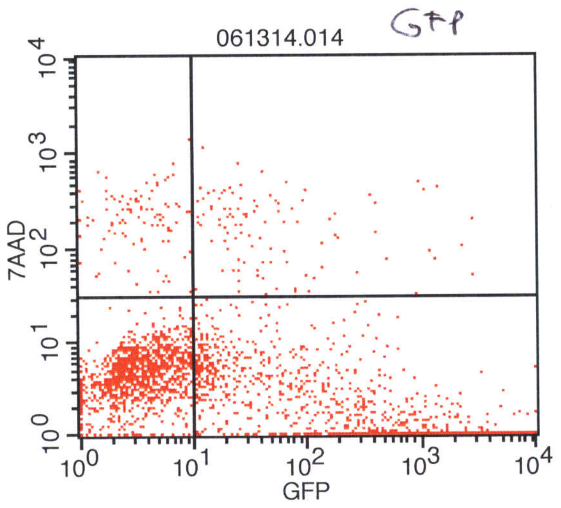

File: 061314.014
Sample ID:
Acquisition Date: 13-Jun-14
Gated Events: 20000
X Parameter: FSC-H (Linear)

Region	Events	% Gated	%
R2	1437	7.18	

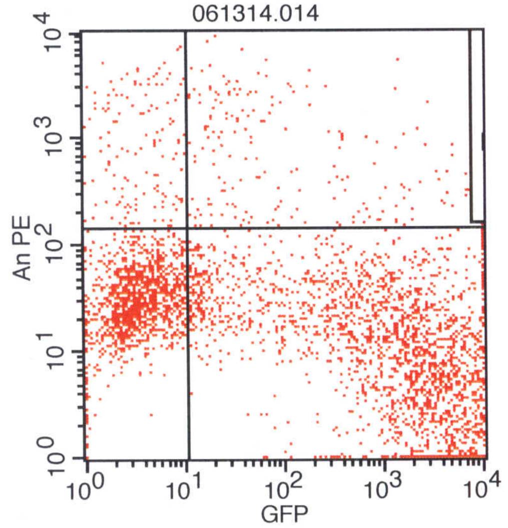

File: 061314.014
Sample ID:
Acquisition Date: 13-Jun-14
Gated Events: 18563
X Parameter: GFP (Log)
Quad Location: 10, 30

Log Data Units: Linear Values
Patient ID:
Gate: G1
Total Events: 20000
Y Parameter: 7AAD (Log)

Quad	Events	% Gated	% Total	X Mean	X Geo Mean	Y Mean	Y Geo Mean
UL	547	2.95	2.73	4.30	3.59	258.85	204.06
UR	540	2.91	2.70	243.95	49.79	226.57	170.40
LL	5826	31.39	29.13	3.95	3.37	5.17	4.29
LR	11650	62.76	58.25	2734.36	952.03	1.99	1.40

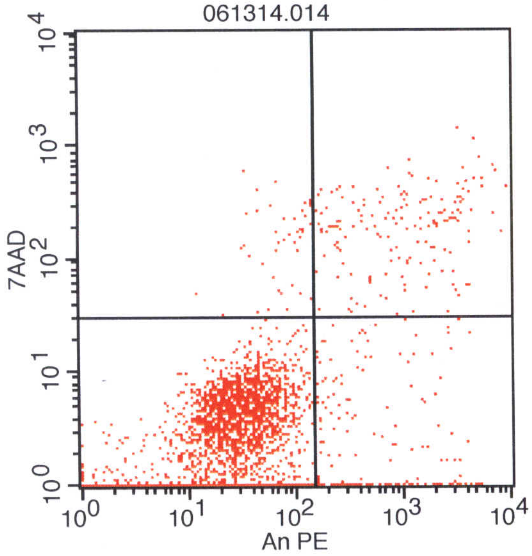

File: 061314.014
Sample ID:
Acquisition Date: 13-Jun-14
Gated Events: 18563
X Parameter: GFP (Log)
Quad Location: 11, 145

Log Data Units: Linear Values
Patient ID:
Gate: G1
Total Events: 20000
Y Parameter: An PE (Log)

Quad	Events	% Gated	% Total	X Mean	X Geo Mean	Y Mean	Y Geo Mean
UL	778	4.19	3.89	4.54	3.75	1264.55	744.15
UR	958	5.16	4.79	791.35	109.03	1338.66	746.68
LL	5741	30.93	28.71	4.07	3.44	36.79	28.67
LR	11086	59.72	55.43	2816.83	1055.42	21.36	9.62

File: 061314.014
Sample ID:
Acquisition Date: 13-Jun-14
Gated Events: 18563
X Parameter: An PE (Log)
Quad Location: 149, 30

Log Data Units: Linear Values
Patient ID:
Gate: G1
Total Events: 20000
Y Parameter: 7AAD (Log)

Quad	Events	% Gated	% Total	X Mean	X Geo Mean	Y Mean	Y Geo Mean
UL	209	1.13	1.04	84.20	65.39	194.71	150.80
UR	877	4.72	4.38	1453.16	879.76	254.52	196.70
LL	16648	89.68	83.24	26.12	13.75	2.96	2.01
LR	829	4.47	4.15	1191.10	663.61	4.89	2.49

EV Syn gfp pcDNA

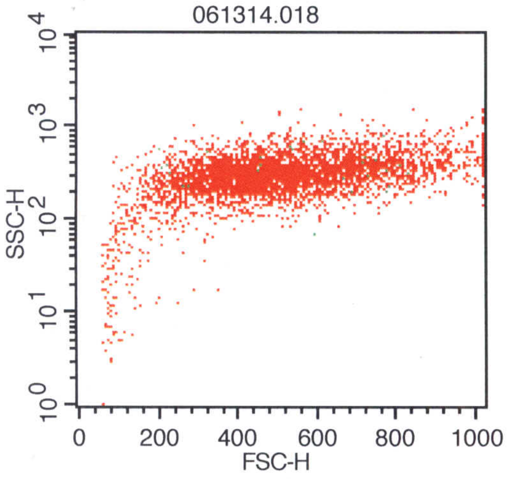

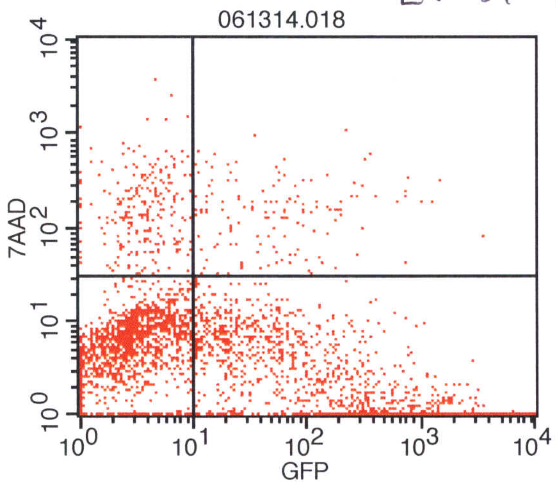

File: 061314.018
Sample ID:
Acquisition Date: 13-Jun-14
Gated Events: 20000
X Parameter: FSC-H (Linear)

Region	Events	% Gated	%
R2	263	1.31	

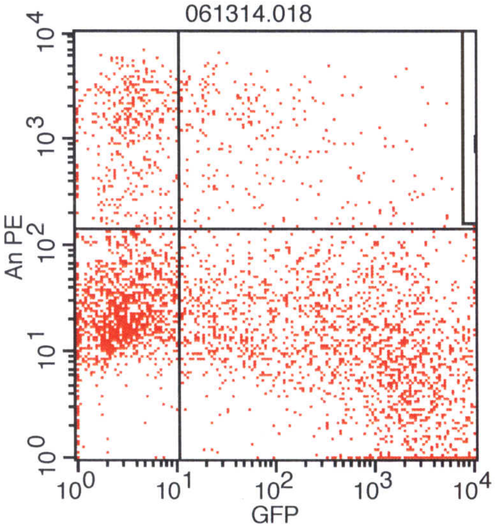

File: 061314.018
Sample ID:
Acquisition Date: 13-Jun-14
Gated Events: 19737
X Parameter: GFP (Log)
Quad Location: 10, 30

Log Data Units: Linear Values
Patient ID:
Gate: G1
Total Events: 20000
Y Parameter: 7AAD (Log)

Quad	Events	% Gated	% Total	X Mean	X Geo Mean	Y Mean	Y Geo Mean
UL	1227	6.22	6.13	3.87	3.34	211.45	143.04
UR	702	3.56	3.51	125.31	48.71	197.75	137.90
LL	7112	36.03	35.56	3.38	2.80	6.76	5.33
LR	10696	54.19	53.48	1711.91	457.16	2.85	1.77

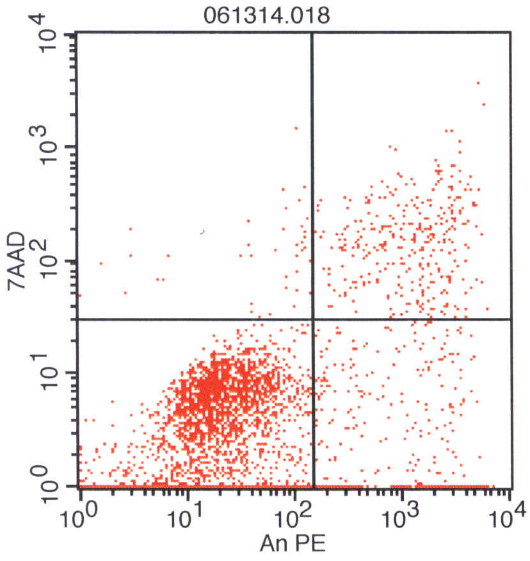

File: 061314.018
Sample ID:
Acquisition Date: 13-Jun-14
Gated Events: 19737
X Parameter: GFP (Log)
Quad Location: 11, 145

Log Data Units: Linear Values
Patient ID:
Gate: G1
Total Events: 20000
Y Parameter: An PE (Log)

Quad	Events	% Gated	% Total	X Mean	X Geo Mean	Y Mean	Y Geo Mean
UL	2145	10.87	10.72	3.87	3.22	1637.31	1051.32
UR	1397	7.08	6.98	530.58	96.26	1463.44	896.81
LL	6369	32.27	31.85	3.51	2.86	30.38	22.10
LR	9826	49.78	49.13	1796.82	520.10	20.56	10.46

File: 061314.018
Sample ID:
Acquisition Date: 13-Jun-14
Gated Events: 19737
X Parameter: An PE (Log)
Quad Location: 149, 30

Log Data Units: Linear Values
Patient ID:
Gate: G1
Total Events: 20000
Y Parameter: 7AAD (Log)

Quad	Events	% Gated	% Total	X Mean	X Geo Mean	Y Mean	Y Geo Mean
UL	148	0.75	0.74	81.02	62.29	170.25	116.10
UR	1778	9.01	8.89	1608.34	1100.65	209.78	143.84
LL	16074	81.44	80.37	24.11	13.90	4.24	2.73
LR	1737	8.80	8.69	1550.28	910.09	6.08	3.03

Ev Syn gfp pcDNA

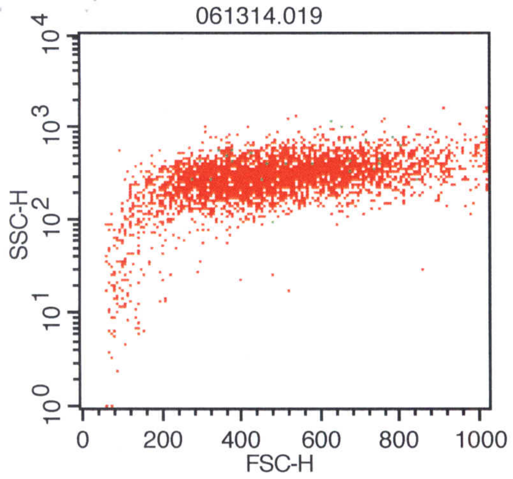

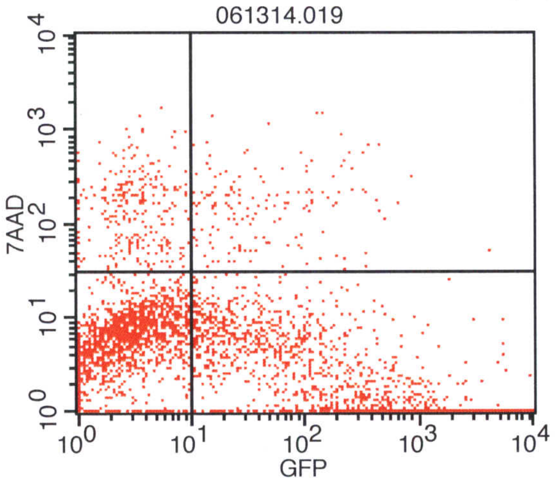

File: 061314.019
Sample ID:
Acquisition Date: 13-Jun-14
Gated Events: 20000
X Parameter: FSC-H (Linear)

Region	Events	% Gated	%
R2	267	1.33	

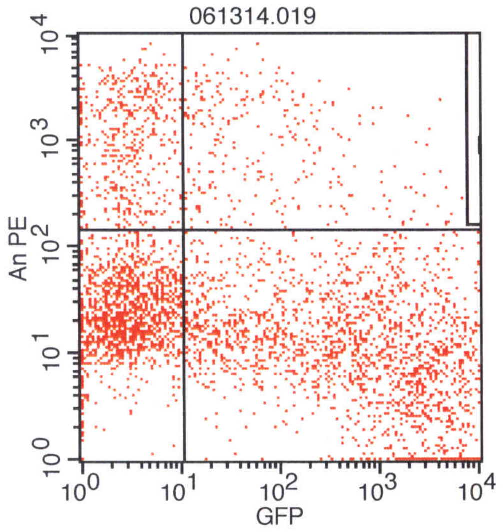

File: 061314.019
Sample ID:
Acquisition Date: 13-Jun-14
Gated Events: 19733
X Parameter: GFP (Log)
Quad Location: 10, 30

Log Data Units: Linear Values
Patient ID:
Gate: G1
Total Events: 20000
Y Parameter: 7AAD (Log)

Quad	Events	% Gated	% Total	X Mean	X Geo Mean	Y Mean	Y Geo Mean
UL	1319	6.68	6.59	3.84	3.29	212.96	144.02
UR	791	4.01	3.96	99.10	43.69	211.19	145.14
LL	7010	35.52	35.05	3.38	2.79	6.91	5.46
LR	10613	53.78	53.06	1664.67	438.86	3.02	1.83

File: 061314.019
Sample ID:
Acquisition Date: 13-Jun-14
Gated Events: 19733
X Parameter: GFP (Log)
Quad Location: 11, 145

Log Data Units: Linear Values
Patient ID:
Gate: G1
Total Events: 20000
Y Parameter: An PE (Log)

Quad	Events	% Gated	% Total	X Mean	X Geo Mean	Y Mean	Y Geo Mean
UL	2214	11.22	11.07	3.79	3.15	1650.90	1079.06
UR	1466	7.43	7.33	405.43	80.28	1484.04	919.44
LL	6282	31.83	31.41	3.52	2.87	30.57	22.23
LR	9771	49.52	48.85	1755.14	500.89	20.72	11.06

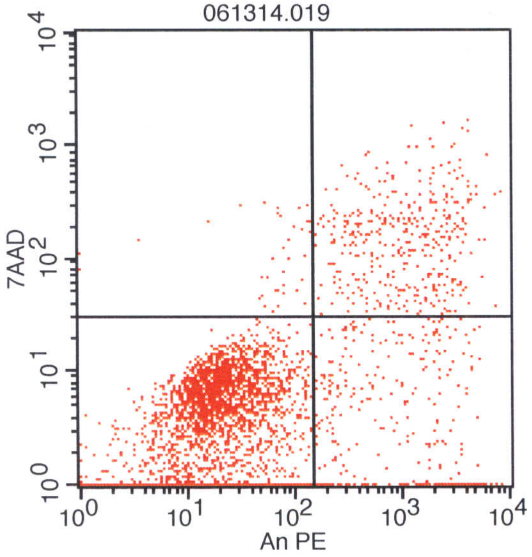

File: 061314.019
Sample ID:
Acquisition Date: 13-Jun-14
Gated Events: 19733
X Parameter: An PE (Log)
Quad Location: 149, 30

Log Data Units: Linear Values
Patient ID:
Gate: G1
Total Events: 20000
Y Parameter: 7AAD (Log)

Quad	Events	% Gated	% Total	X Mean	X Geo Mean	Y Mean	Y Geo Mean
UL	180	0.91	0.90	80.03	63.02	182.11	118.19
UR	1925	9.76	9.62	1603.77	1104.24	215.59	147.77
LL	15895	80.55	79.47	24.11	14.34	4.32	2.77
LR	1733	8.78	8.67	1581.20	942.06	6.83	3.39

EV Smgfp pcDNA

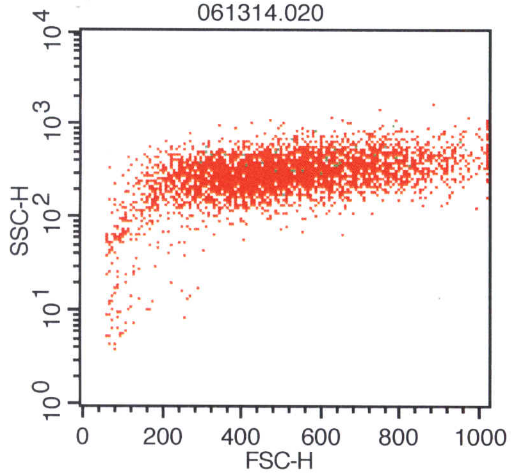

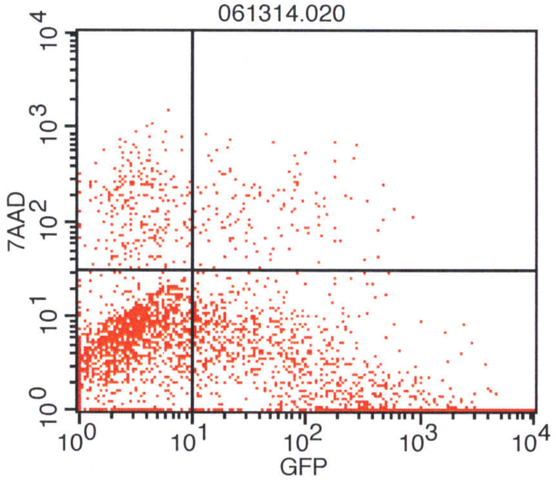

File: 061314.020
Sample ID:
Acquisition Date: 13-Jun-14
Gated Events: 20000
X Parameter: FSC-H (Linear)

Region	Events	% Gated	%
R2	322	1.61	

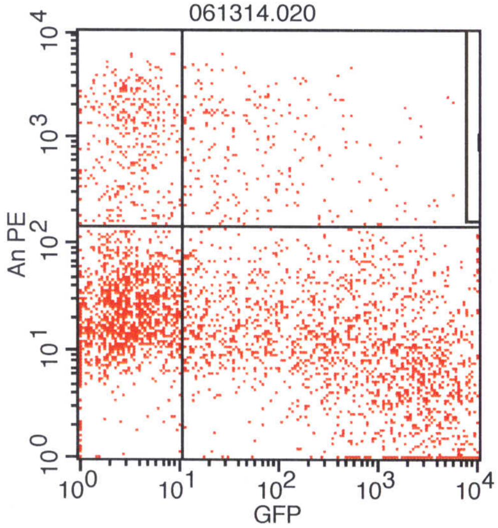

File: 061314.020
Sample ID:
Acquisition Date: 13-Jun-14
Gated Events: 19678
X Parameter: GFP (Log)
Quad Location: 10, 30

Log Data Units: Linear Values
Patient ID:
Gate: G1
Total Events: 20000
Y Parameter: 7AAD (Log)

Quad	Events	% Gated	% Total	X Mean	X Geo Mean	Y Mean	Y Geo Mean
UL	1322	6.72	6.61	3.85	3.29	205.73	142.97
UR	748	3.80	3.74	114.76	47.95	202.45	141.51
LL	7064	35.90	35.32	3.38	2.79	6.43	5.05
LR	10544	53.58	52.72	1761.03	461.15	2.75	1.71

File: 061314.020
Sample ID:
Acquisition Date: 13-Jun-14
Gated Events: 19678
X Parameter: GFP (Log)
Quad Location: 11, 145

Log Data Units: Linear Values
Patient ID:
Gate: G1
Total Events: 20000
Y Parameter: An PE (Log)

Quad	Events	% Gated	% Total	X Mean	X Geo Mean	Y Mean	Y Geo Mean
UL	2034	10.34	10.17	3.84	3.20	1421.38	936.41
UR	1304	6.63	6.52	363.99	78.07	1343.15	847.25
LL	6523	33.15	32.62	3.51	2.87	28.90	20.27
LR	9817	49.89	49.09	1851.66	525.02	18.29	9.27

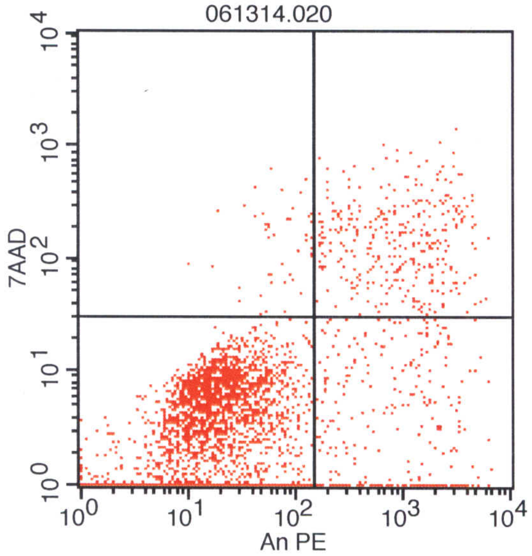

File: 061314.020
Sample ID:
Acquisition Date: 13-Jun-14
Gated Events: 19678
X Parameter: An PE (Log)
Quad Location: 149, 30

Log Data Units: Linear Values
Patient ID:
Gate: G1
Total Events: 20000
Y Parameter: 7AAD (Log)

Quad	Events	% Gated	% Total	X Mean	X Geo Mean	Y Mean	Y Geo Mean
UL	270	1.37	1.35	80.53	65.15	170.22	117.38
UR	1797	9.13	8.98	1336.02	897.73	209.99	147.03
LL	16100	81.82	80.50	21.79	12.38	3.99	2.59
LR	1511	7.68	7.56	1480.68	936.93	6.73	3.36

87P Syn gfp pcDNA

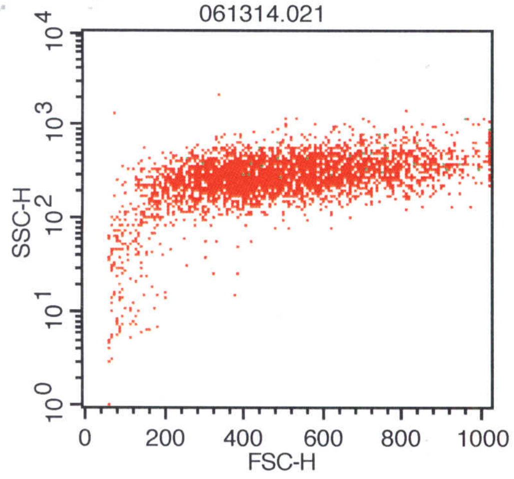

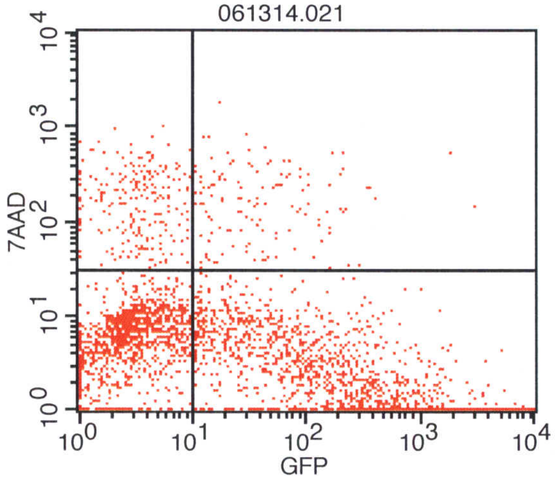

File: 061314.021
Sample ID:
Acquisition Date: 13-Jun-14
Gated Events: 20000
X Parameter: FSC-H (Linear)

Region	Events	% Gated	%
R2	274	1.37	

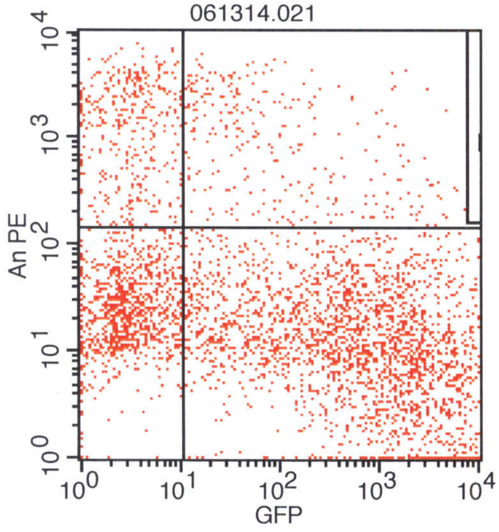

File: 061314.021
Sample ID:
Acquisition Date: 13-Jun-14
Gated Events: 19726
X Parameter: GFP (Log)
Quad Location: 10, 30

Log Data Units: Linear Values
Patient ID:
Gate: G1
Total Events: 20000
Y Parameter: 7AAD (Log)

Quad	Events	% Gated	% Total	X Mean	X Geo Mean	Y Mean	Y Geo Mean
UL	1123	5.69	5.62	3.88	3.29	227.72	163.30
UR	673	3.41	3.36	110.87	44.33	209.97	150.19
LL	5572	28.25	27.86	3.49	2.88	6.77	5.30
LR	12358	62.65	61.79	1631.82	489.70	2.69	1.71

File: 061314.021
Sample ID:
Acquisition Date: 13-Jun-14
Gated Events: 19726
X Parameter: GFP (Log)
Quad Location: 11, 145

Log Data Units: Linear Values
Patient ID:
Gate: G1
Total Events: 20000
Y Parameter: An PE (Log)

Quad	Events	% Gated	% Total	X Mean	X Geo Mean	Y Mean	Y Geo Mean
UL	1900	9.63	9.50	3.90	3.19	1864.03	1216.82
UR	1409	7.14	7.04	545.53	100.12	1507.55	903.55
LL	4927	24.98	24.64	3.60	2.96	30.83	22.51
LR	11490	58.25	57.45	1694.57	540.28	21.12	11.29

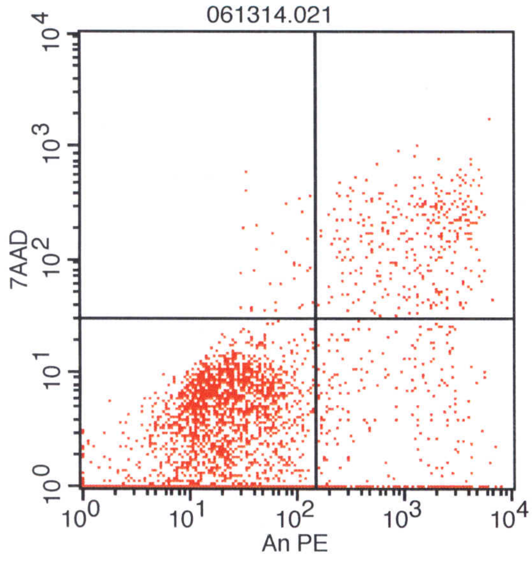

File: 061314.021
Sample ID:
Acquisition Date: 13-Jun-14
Gated Events: 19726
X Parameter: An PE (Log)
Quad Location: 149, 30

Log Data Units: Linear Values
Patient ID:
Gate: G1
Total Events: 20000
Y Parameter: 7AAD (Log)

Quad	Events	% Gated	% Total	X Mean	X Geo Mean	Y Mean	Y Geo Mean
UL	119	0.60	0.60	80.37	70.31	145.43	101.77
UR	1674	8.49	8.37	1835.84	1277.78	226.79	163.79
LL	16321	82.74	81.61	23.80	13.77	3.76	2.39
LR	1612	8.17	8.06	1606.21	918.92	5.94	2.97

87P Syn gfp pcDNA3.1

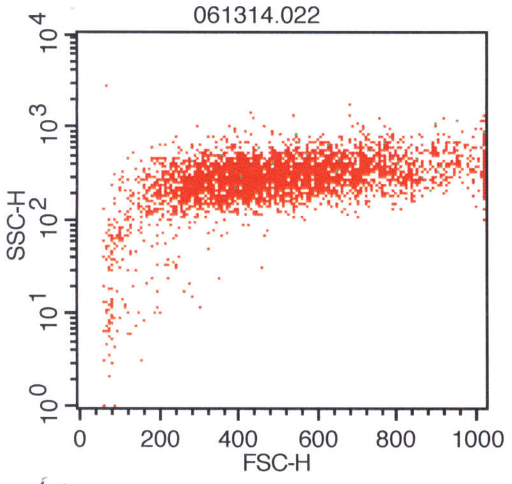

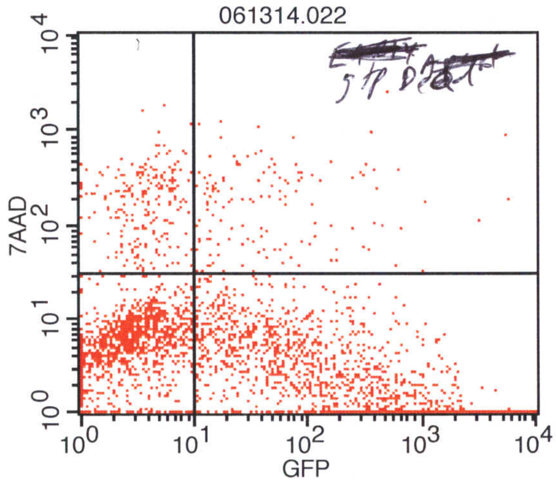

File: 061314.022
Sample ID:
Acquisition Date: 13-Jun-14
Gated Events: 20000
X Parameter: FSC-H (Linear)

Region	Events	% Gated	%
R2	231	1.16	

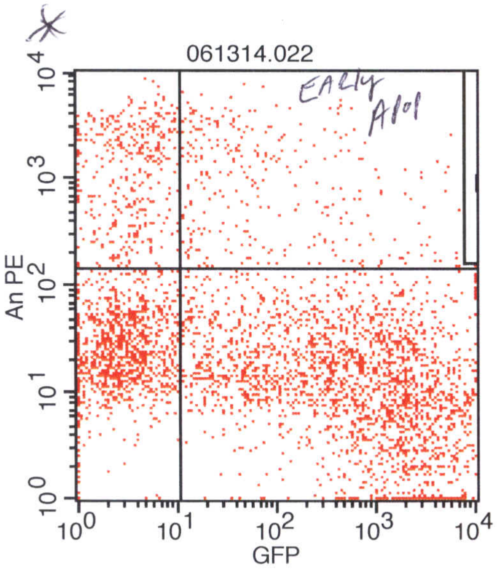

File: 061314.022
Sample ID:
Acquisition Date: 13-Jun-14
Gated Events: 19769
X Parameter: GFP (Log)
Quad Location: 10, 30

Log Data Units: Linear Values
Patient ID:
Gate: G1
Total Events: 20000
Y Parameter: 7AAD (Log)

Quad	Events	% Gated	% Total	X Mean	X Geo Mean	Y Mean	Y Geo Mean
UL	1156	5.85	5.78	3.97	3.36	229.00	160.98
UR	776	3.93	3.88	125.74	44.46	225.52	156.66
LL	5604	28.35	28.02	3.42	2.82	6.80	5.29
LR	12233	61.88	61.17	1603.24	479.99	2.81	1.76

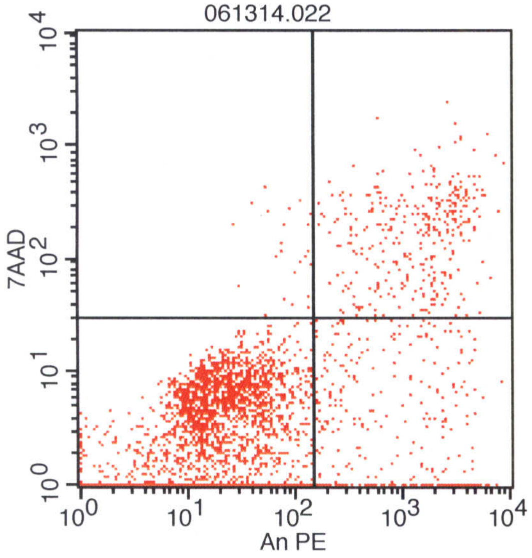

File: 061314.022
Sample ID:
Acquisition Date: 13-Jun-14
Gated Events: 19769
X Parameter: GFP (Log)
Quad Location: 11, 145

Log Data Units: Linear Values
Patient ID:
Gate: G1
Total Events: 20000
Y Parameter: An PE (Log)

Quad	Events	% Gated	% Total	X Mean	X Geo Mean	Y Mean	Y Geo Mean
UL	1936	9.79	9.68	3.94	3.23	1870.07	1241.95
UR	1529	7.73	7.65	521.41	97.99	1508.69	881.85
LL	4957	25.07	24.79	3.53	2.89	31.75	22.95
LR	11347	57.40	56.73	1666.64	528.55	21.11	11.25

File: 061314.022
Sample ID:
Acquisition Date: 13-Jun-14
Gated Events: 19769
X Parameter: An PE (Log)
Quad Location: 149, 30

Log Data Units: Linear Values
Patient ID:
Gate: G1
Total Events: 20000
Y Parameter: 7AAD (Log)

Quad	Events	% Gated	% Total	X Mean	X Geo Mean	Y Mean	Y Geo Mean
UL	138	0.70	0.69	89.61	76.86	135.09	97.98
UR	1789	9.05	8.95	1821.07	1278.81	235.29	166.08
LL	16201	81.95	81.01	24.06	13.84	3.84	2.44
LR	1641	8.30	8.21	1623.53	915.11	6.29	3.01

87P S7-GFP pcDNA3.1

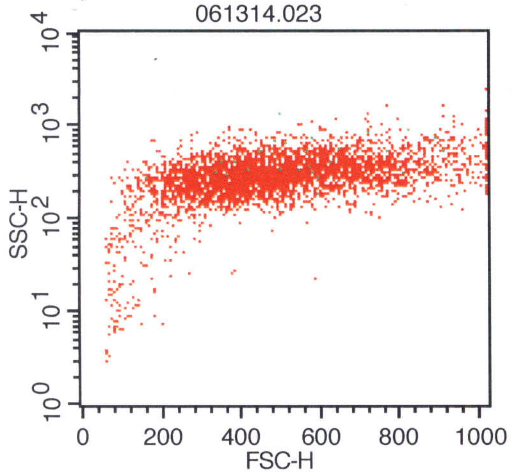

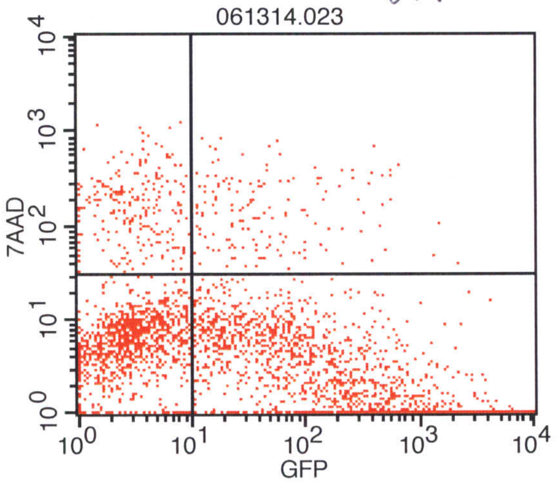

File: 061314.023
Sample ID:
Acquisition Date: 13-Jun-14
Gated Events: 20000
X Parameter: FSC-H (Linear)

Region	Events	% Gated	%
R2	240	1.20	

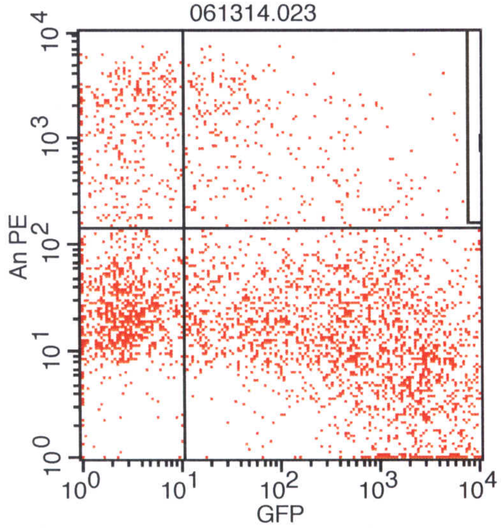

File: 061314.023
Sample ID:
Acquisition Date: 13-Jun-14
Gated Events: 19760
X Parameter: GFP (Log)
Quad Location: 10, 30

Log Data Units: Linear Values
Patient ID:
Gate: G1
Total Events: 20000
Y Parameter: 7AAD (Log)

Quad	Events	% Gated	% Total	X Mean	X Geo Mean	Y Mean	Y Geo Mean
UL	1210	6.12	6.05	3.92	3.31	219.08	156.84
UR	769	3.89	3.84	112.85	44.48	210.45	147.63
LL	5462	27.64	27.31	3.45	2.85	6.79	5.33
LR	12319	62.34	61.59	1633.21	501.01	2.73	1.72

File: 061314.023
Sample ID:
Acquisition Date: 13-Jun-14
Gated Events: 19760
X Parameter: GFP (Log)
Quad Location: 11, 145

Log Data Units: Linear Values
Patient ID:
Gate: G1
Total Events: 20000
Y Parameter: An PE (Log)

Quad	Events	% Gated	% Total	X Mean	X Geo Mean	Y Mean	Y Geo Mean
UL	1937	9.80	9.69	4.04	3.32	1831.98	1176.25
UR	1513	7.66	7.56	499.94	96.70	1531.12	915.91
LL	4888	24.74	24.44	3.55	2.90	31.15	22.77
LR	11422	57.80	57.11	1702.71	557.53	20.63	10.86

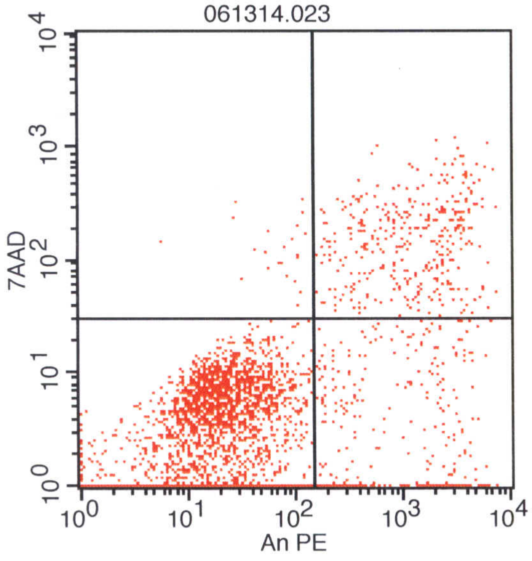

File: 061314.023
Sample ID:
Acquisition Date: 13-Jun-14
Gated Events: 19760
X Parameter: An PE (Log)
Quad Location: 149, 30

Log Data Units: Linear Values
Patient ID:
Gate: G1
Total Events: 20000
Y Parameter: 7AAD (Log)

Quad	Events	% Gated	% Total	X Mean	X Geo Mean	Y Mean	Y Geo Mean
UL	129	0.65	0.65	77.07	61.18	155.27	111.58
UR	1846	9.34	9.23	1792.09	1220.28	220.35	157.18
LL	16200	81.98	81.00	23.51	13.43	3.73	2.37
LR	1585	8.02	7.92	1611.43	909.93	6.48	3.20

VHHP Sin gfp pcDNA 3.1

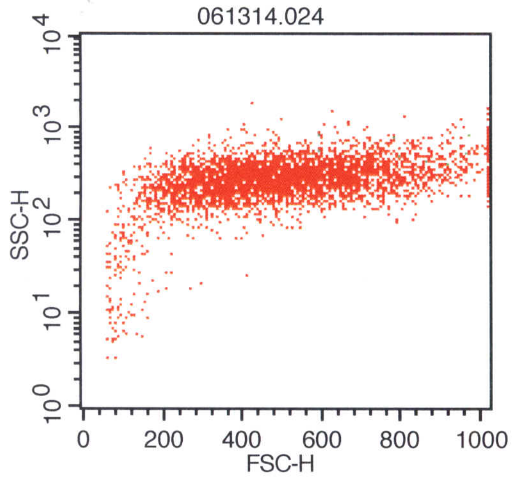

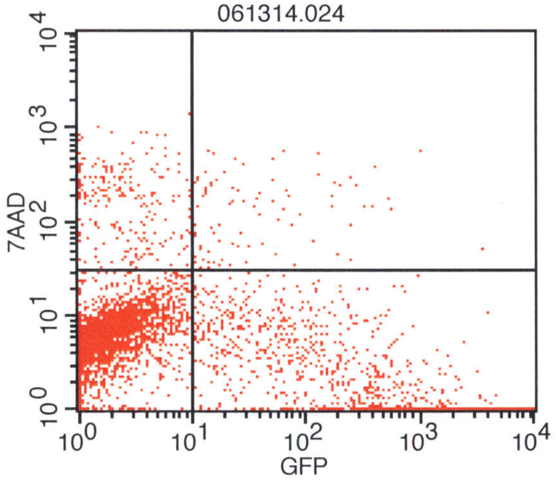

File: 061314.024
Sample ID:
Acquisition Date: 13-Jun-14
Gated Events: 20000
X Parameter: FSC-H (Linear)

Region	Events	% Gated	%
R2	61	0.30	

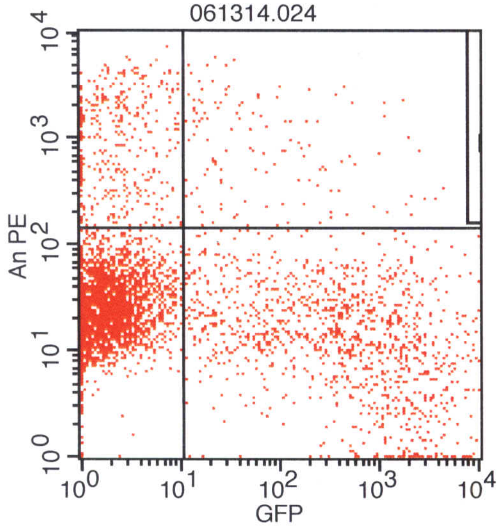

File: 061314.024
Sample ID:
Acquisition Date: 13-Jun-14
Gated Events: 19939
X Parameter: GFP (Log)
Quad Location: 10, 30

Log Data Units: Linear Values
Patient ID:
Gate: G1
Total Events: 20000
Y Parameter: 7AAD (Log)

Quad	Events	% Gated	% Total	X Mean	X Geo Mean	Y Mean	Y Geo Mean
UL	1291	6.47	6.46	3.11	2.54	218.38	152.69
UR	398	2.00	1.99	129.88	41.34	180.95	119.98
LL	11841	59.39	59.20	2.41	2.00	6.89	5.63
LR	6409	32.14	32.05	1243.15	369.56	3.37	2.00

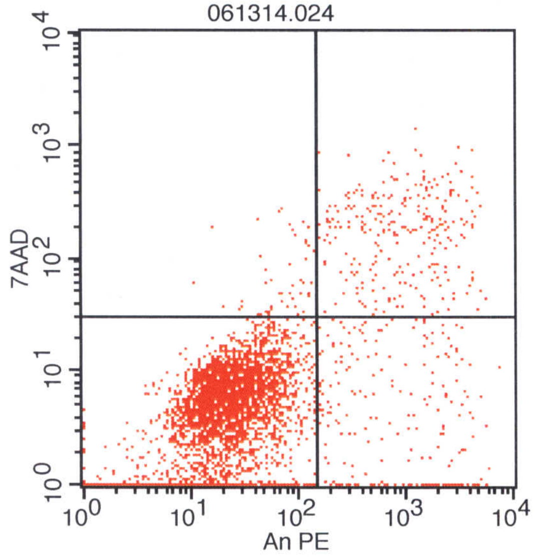

File: 061314.024
Sample ID:
Acquisition Date: 13-Jun-14
Gated Events: 19939
X Parameter: GFP (Log)
Quad Location: 11, 145

Log Data Units: Linear Values
Patient ID:
Gate: G1
Total Events: 20000
Y Parameter: An PE (Log)

Quad	Events	% Gated	% Total	X Mean	X Geo Mean	Y Mean	Y Geo Mean
UL	1871	9.38	9.35	3.11	2.51	1459.19	929.68
UR	618	3.10	3.09	356.94	77.55	1214.51	754.81
LL	11361	56.98	56.81	2.44	2.00	30.85	24.00
LR	6089	30.54	30.45	1280.57	397.98	22.91	13.00

File: 061314.024
Sample ID:
Acquisition Date: 13-Jun-14
Gated Events: 19939
X Parameter: An PE (Log)
Quad Location: 149, 30

Log Data Units: Linear Values
Patient ID:
Gate: G1
Total Events: 20000
Y Parameter: 7AAD (Log)

Quad	Events	% Gated	% Total	X Mean	X Geo Mean	Y Mean	Y Geo Mean
UL	236	1.18	1.18	77.74	65.76	120.26	84.16
UR	1448	7.26	7.24	1426.79	982.83	224.74	158.35
LL	17237	86.45	86.19	27.56	19.10	5.57	3.92
LR	1018	5.11	5.09	1386.38	789.12	7.26	3.92

VHHP [illegible] pcDNA3.1

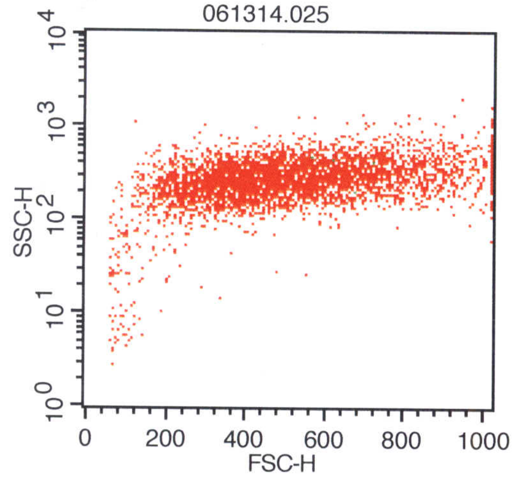

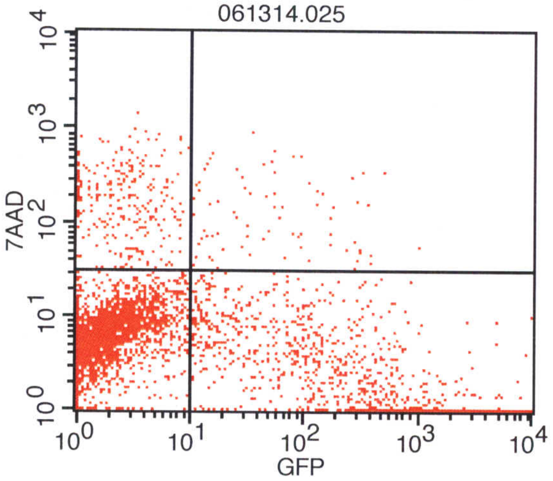

File: 061314.025
Sample ID:
Acquisition Date: 13-Jun-14
Gated Events: 20000
X Parameter: FSC-H (Linear)

Region	Events	% Gated	%
R2	59	0.29	

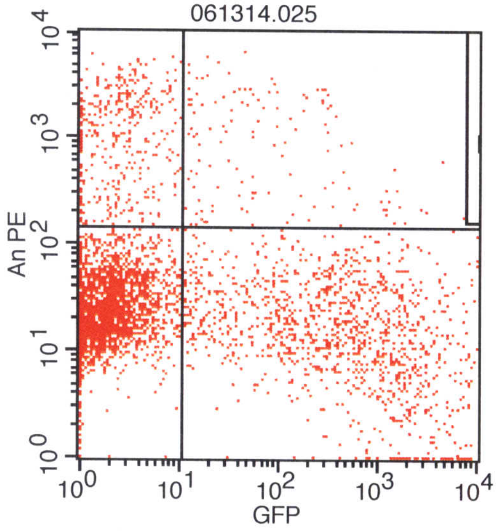

File: 061314.025
Sample ID:
Acquisition Date: 13-Jun-14
Gated Events: 19941
X Parameter: GFP (Log)
Quad Location: 10, 30

Log Data Units: Linear Values
Patient ID:
Gate: G1
Total Events: 20000
Y Parameter: 7AAD (Log)

Quad	Events	% Gated	% Total	X Mean	X Geo Mean	Y Mean	Y Geo Mean
UL	1384	6.94	6.92	3.12	2.56	227.33	160.05
UR	410	2.06	2.05	126.43	42.50	189.83	130.62
LL	11707	58.71	58.54	2.41	2.00	7.00	5.72
LR	6440	32.30	32.20	1204.07	362.95	3.41	2.02

File: 061314.025
Sample ID:
Acquisition Date: 13-Jun-14
Gated Events: 19941
X Parameter: GFP (Log)
Quad Location: 11, 145

Log Data Units: Linear Values
Patient ID:
Gate: G1
Total Events: 20000
Y Parameter: An PE (Log)

Quad	Events	% Gated	% Total	X Mean	X Geo Mean	Y Mean	Y Geo Mean
UL	1999	10.02	9.99	3.12	2.53	1470.98	930.77
UR	660	3.31	3.30	479.85	93.19	1283.43	770.62
LL	11195	56.14	55.97	2.45	2.01	30.56	23.68
LR	6087	30.53	30.44	1230.21	386.61	23.40	13.88

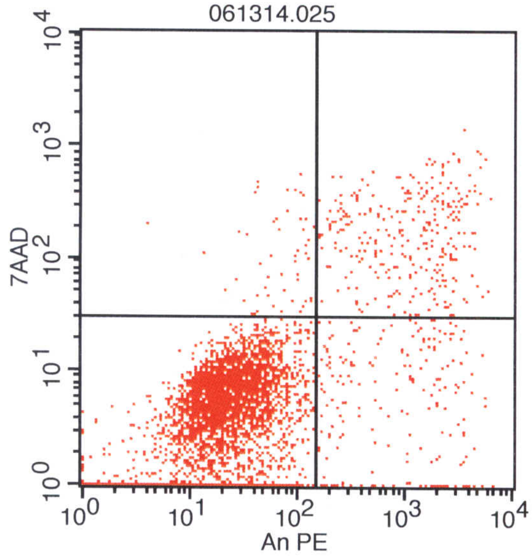

File: 061314.025
Sample ID:
Acquisition Date: 13-Jun-14
Gated Events: 19941
X Parameter: An PE (Log)
Quad Location: 149, 30

Log Data Units: Linear Values
Patient ID:
Gate: G1
Total Events: 20000
Y Parameter: 7AAD (Log)

Quad	Events	% Gated	% Total	X Mean	X Geo Mean	Y Mean	Y Geo Mean
UL	251	1.26	1.26	77.94	63.61	144.48	94.79
UR	1540	7.72	7.70	1489.97	1011.79	231.23	165.68
LL	17052	85.51	85.26	27.45	19.33	5.59	3.93
LR	1098	5.51	5.49	1356.93	765.64	7.92	4.28

VH14 P Si- gfp pcDNA3.1

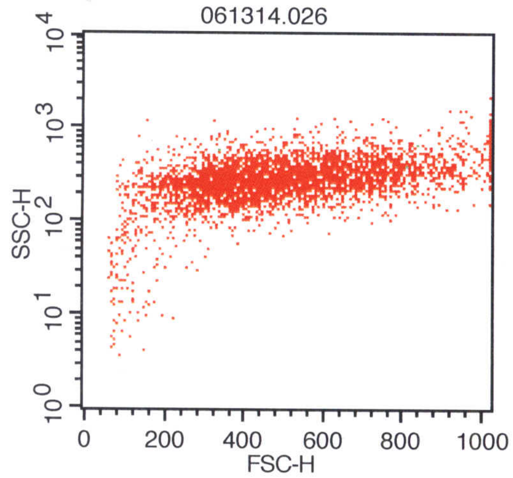

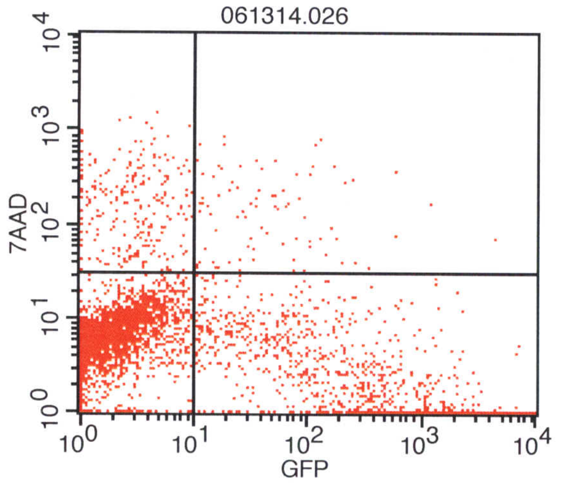

File: 061314.026
Sample ID:
Acquisition Date: 13-Jun-14
Gated Events: 20000
X Parameter: FSC-H (Linear)

Region	Events	% Gated	%
R2	39	0.19	

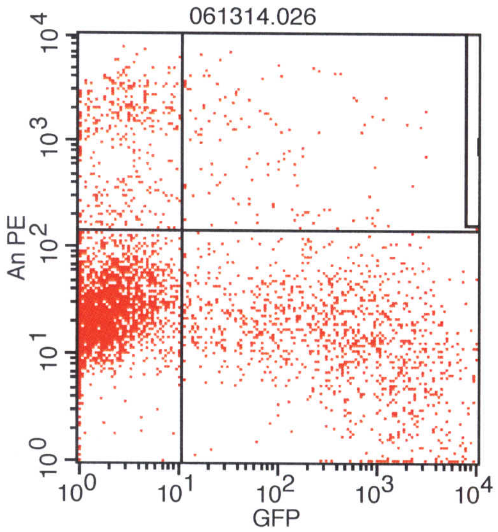

File: 061314.026
Sample ID:
Acquisition Date: 13-Jun-14
Gated Events: 19961
X Parameter: GFP (Log)
Quad Location: 10, 30

Log Data Units: Linear Values
Patient ID:
Gate: G1
Total Events: 20000
Y Parameter: 7AAD (Log)

Quad	Events	% Gated	% Total	X Mean	X Geo Mean	Y Mean	Y Geo Mean
UL	1368	6.85	6.84	3.35	2.73	232.93	157.87
UR	440	2.20	2.20	126.88	43.71	194.97	126.60
LL	11747	58.85	58.74	2.47	2.03	7.02	5.71
LR	6406	32.09	32.03	1165.87	349.43	3.50	2.09

File: 061314.026
Sample ID:
Acquisition Date: 13-Jun-14
Gated Events: 19961
X Parameter: GFP (Log)
Quad Location: 11, 145

Log Data Units: Linear Values
Patient ID:
Gate: G1
Total Events: 20000
Y Parameter: An PE (Log)

Quad	Events	% Gated	% Total	X Mean	X Geo Mean	Y Mean	Y Geo Mean
UL	1959	9.81	9.79	3.23	2.63	1496.41	956.05
UR	694	3.48	3.47	340.14	83.04	1302.05	779.20
LL	11245	56.33	56.23	2.50	2.04	31.04	24.08
LR	6063	30.37	30.31	1201.95	373.03	23.04	13.41

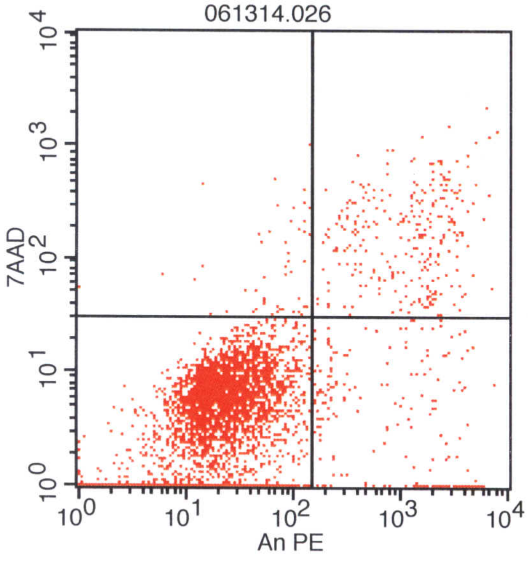

File: 061314.026
Sample ID:
Acquisition Date: 13-Jun-14
Gated Events: 19961
X Parameter: An PE (Log)
Quad Location: 149, 30

Log Data Units: Linear Values
Patient ID:
Gate: G1
Total Events: 20000
Y Parameter: 7AAD (Log)

Quad	Events	% Gated	% Total	X Mean	X Geo Mean	Y Mean	Y Geo Mean
UL	291	1.46	1.46	79.07	66.19	134.89	89.66
UR	1511	7.57	7.56	1537.25	1041.02	241.56	166.17
LL	17048	85.41	85.24	27.58	19.28	5.68	4.01
LR	1111	5.57	5.56	1357.10	789.48	7.41	3.98

EV 571 Q72G+P

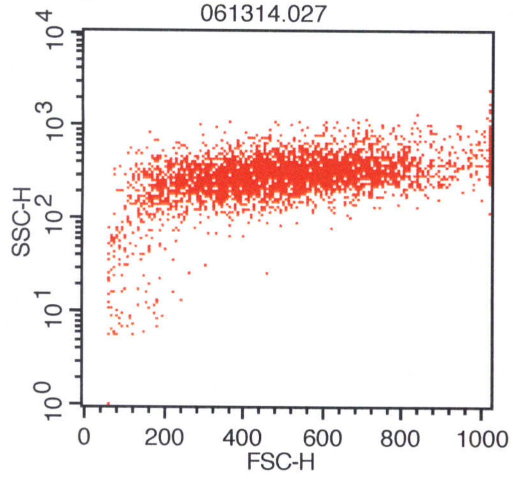

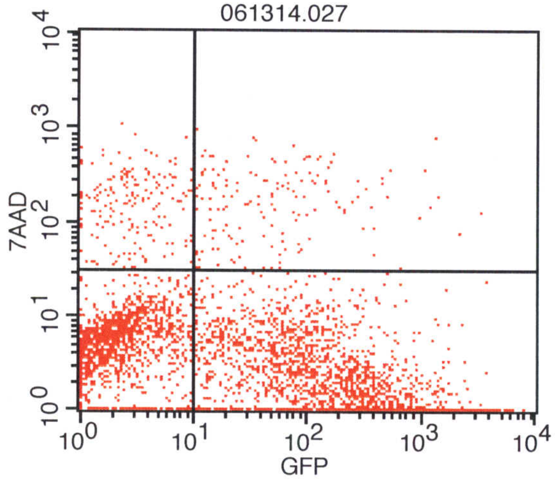

File: 061314.027
Sample ID:
Acquisition Date: 13-Jun-14
Gated Events: 20000
X Parameter: FSC-H (Linear)

Region	Events	% Gated	%
R2	4	0.02	

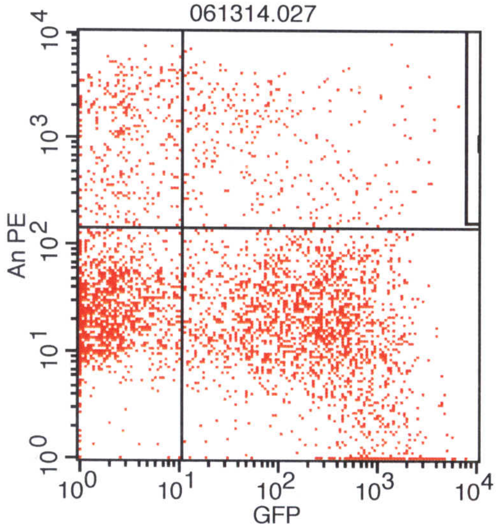

File: 061314.027
Sample ID:
Acquisition Date: 13-Jun-14
Gated Events: 19996
X Parameter: GFP (Log)
Quad Location: 10, 30

Log Data Units: Linear Values
Patient ID:
Gate: G1
Total Events: 20000
Y Parameter: 7AAD (Log)

Quad	Events	% Gated	% Total	X Mean	X Geo Mean	Y Mean	Y Geo Mean
UL	883	4.42	4.42	3.39	2.78	189.56	140.63
UR	780	3.90	3.90	208.64	54.51	192.05	142.37
LL	6787	33.94	33.93	2.77	2.22	5.94	4.60
LR	11546	57.74	57.73	493.63	202.09	3.16	2.05

File: 061314.027
Sample ID:
Acquisition Date: 13-Jun-14
Gated Events: 19996
X Parameter: GFP (Log)
Quad Location: 11, 145

Log Data Units: Linear Values
Patient ID:
Gate: G1
Total Events: 20000
Y Parameter: An PE (Log)

Quad	Events	% Gated	% Total	X Mean	X Geo Mean	Y Mean	Y Geo Mean
UL	1821	9.11	9.11	3.60	2.89	1724.51	1095.68
UR	1629	8.15	8.14	339.91	88.52	1480.82	908.04
LL	5992	29.97	29.96	2.79	2.20	31.93	23.09
LR	10554	52.78	52.77	502.84	216.94	25.67	14.42

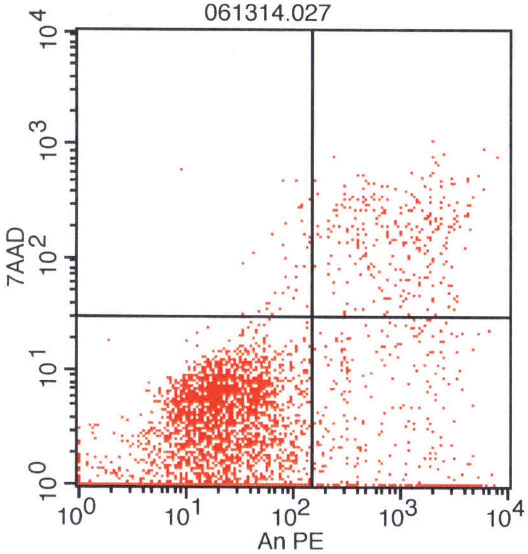

File: 061314.027
Sample ID:
Acquisition Date: 13-Jun-14
Gated Events: 19996
X Parameter: An PE (Log)
Quad Location: 149, 30

Log Data Units: Linear Values
Patient ID:
Gate: G1
Total Events: 20000
Y Parameter: 7AAD (Log)

Quad	Events	% Gated	% Total	X Mean	X Geo Mean	Y Mean	Y Geo Mean
UL	167	0.84	0.83	77.34	59.85	158.46	110.07
UR	1491	7.46	7.46	1471.40	1027.14	194.88	146.23
LL	16406	82.05	82.03	27.63	16.94	4.00	2.75
LR	1932	9.66	9.66	1736.42	1010.96	5.91	2.91

EV Sir Q729H

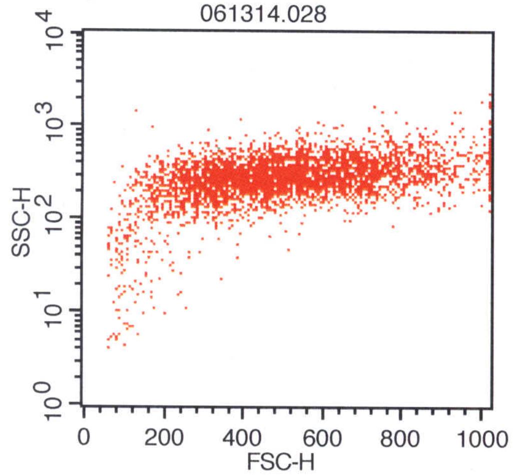

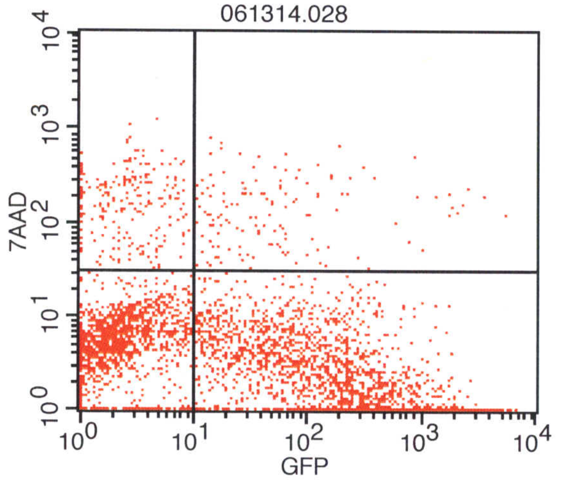

File: 061314.028
Sample ID:
Acquisition Date: 13-Jun-14
Gated Events: 20000
X Parameter: FSC-H (Linear)

Region	Events	% Gated	%
R2	1	0.01	

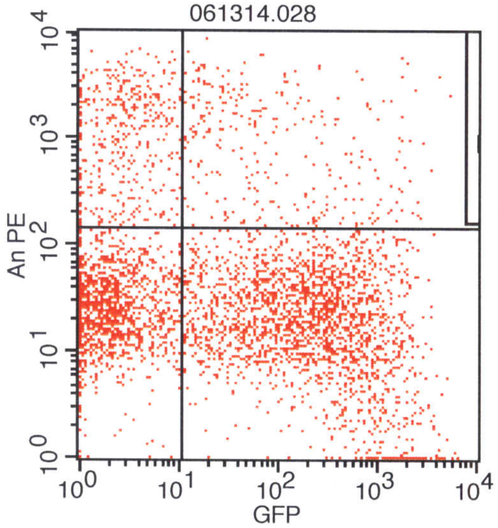

File: 061314.028
Sample ID:
Acquisition Date: 13-Jun-14
Gated Events: 19999
X Parameter: GFP (Log)
Quad Location: 10, 30

Log Data Units: Linear Values
Patient ID:
Gate: G1
Total Events: 20000
Y Parameter: 7AAD (Log)

Quad	Events	% Gated	% Total	X Mean	X Geo Mean	Y Mean	Y Geo Mean
UL	1088	5.44	5.44	3.49	2.81	192.47	143.55
UR	802	4.01	4.01	243.69	57.90	199.87	144.75
LL	6703	33.52	33.52	2.77	2.22	6.26	4.90
LR	11406	57.03	57.03	500.21	203.63	3.33	2.14

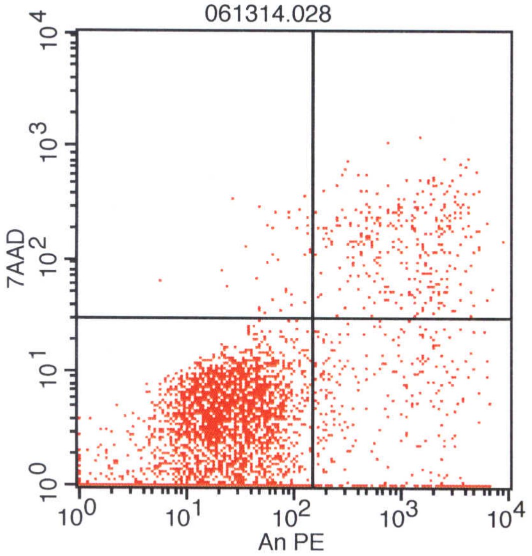

File: 061314.028
Sample ID:
Acquisition Date: 13-Jun-14
Gated Events: 19999
X Parameter: GFP (Log)
Quad Location: 11, 145

Log Data Units: Linear Values
Patient ID:
Gate: G1
Total Events: 20000
Y Parameter: An PE (Log)

Quad	Events	% Gated	% Total	X Mean	X Geo Mean	Y Mean	Y Geo Mean
UL	1994	9.97	9.97	3.64	2.90	1684.47	1060.96
UR	1646	8.23	8.23	387.86	100.10	1461.33	868.14
LL	5919	29.60	29.59	2.77	2.19	32.42	23.46
LR	10440	52.20	52.20	503.94	214.12	27.55	16.49

File: 061314.028
Sample ID:
Acquisition Date: 13-Jun-14
Gated Events: 19999
X Parameter: An PE (Log)
Quad Location: 149, 30

Log Data Units: Linear Values
Patient ID:
Gate: G1
Total Events: 20000
Y Parameter: 7AAD (Log)

Quad	Events	% Gated	% Total	X Mean	X Geo Mean	Y Mean	Y Geo Mean
UL	169	0.85	0.84	78.87	66.42	180.12	124.23
UR	1715	8.58	8.58	1547.61	1058.49	197.72	146.98
LL	16218	81.09	81.09	29.00	18.56	4.19	2.87
LR	1897	9.49	9.49	1637.27	919.76	6.40	3.31

EV 571 Q72[illegible]

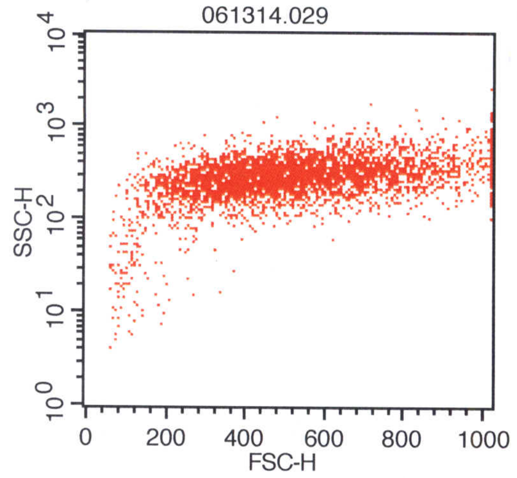

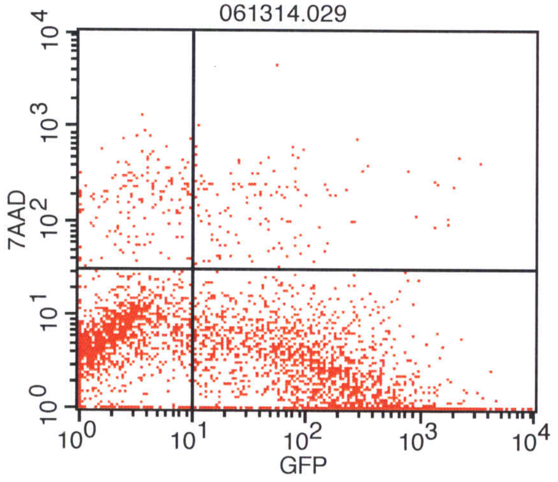

File: 061314.029
Sample ID:
Acquisition Date: 13-Jun-14
Gated Events: 20000
X Parameter: FSC-H (Linear)

Region	Events	% Gated	%
R2	1	0.01	

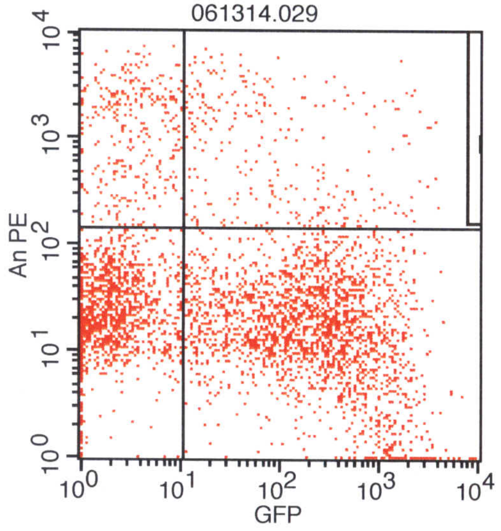

File: 061314.029
Sample ID:
Acquisition Date: 13-Jun-14
Gated Events: 19999
X Parameter: GFP (Log)
Quad Location: 10, 30

Log Data Units: Linear Values
Patient ID:
Gate: G1
Total Events: 20000
Y Parameter: 7AAD (Log)

Quad	Events	% Gated	% Total	X Mean	X Geo Mean	Y Mean	Y Geo Mean
UL	904	4.52	4.52	3.55	2.87	202.72	144.80
UR	786	3.93	3.93	240.44	59.22	204.58	139.63
LL	6655	33.28	33.27	2.81	2.25	6.28	4.88
LR	11654	58.27	58.27	500.18	199.86	3.29	2.11

File: 061314.029
Sample ID:
Acquisition Date: 13-Jun-14
Gated Events: 19999
X Parameter: GFP (Log)
Quad Location: 11, 145

Log Data Units: Linear Values
Patient ID:
Gate: G1
Total Events: 20000
Y Parameter: An PE (Log)

Quad	Events	% Gated	% Total	X Mean	X Geo Mean	Y Mean	Y Geo Mean
UL	1766	8.83	8.83	3.71	2.97	1731.05	1095.37
UR	1653	8.27	8.27	377.68	99.54	1480.63	861.64
LL	5925	29.63	29.62	2.83	2.23	31.58	22.77
LR	10655	53.28	53.27	506.10	211.18	25.89	15.27

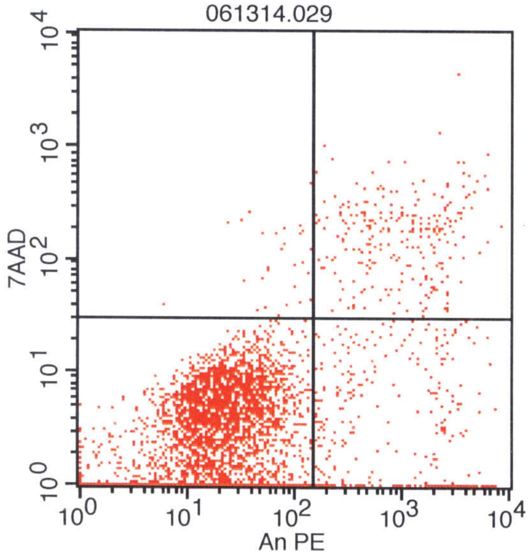

File: 061314.029
Sample ID:
Acquisition Date: 13-Jun-14
Gated Events: 19999
X Parameter: An PE (Log)
Quad Location: 149, 30

Log Data Units: Linear Values
Patient ID:
Gate: G1
Total Events: 20000
Y Parameter: 7AAD (Log)

Quad	Events	% Gated	% Total	X Mean	X Geo Mean	Y Mean	Y Geo Mean
UL	198	0.99	0.99	75.56	60.04	150.24	106.67
UR	1488	7.44	7.44	1546.59	1050.96	211.15	148.57
LL	16419	82.10	82.09	27.61	17.44	4.18	2.83
LR	1894	9.47	9.47	1688.34	954.40	6.13	3.14

87 B [illegible]

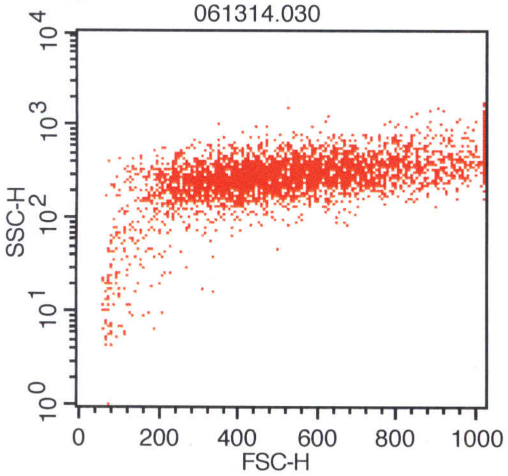

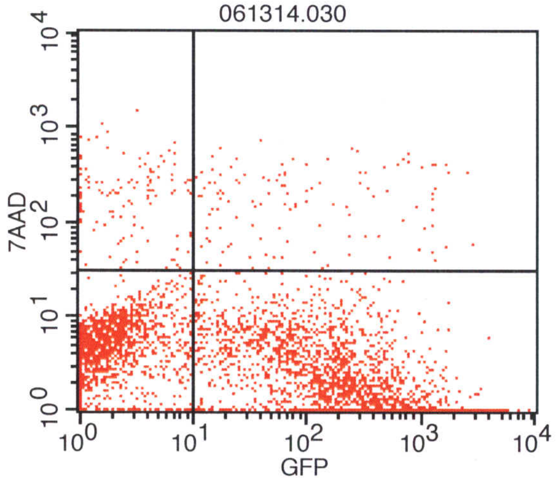

File: 061314.030
Sample ID:
Acquisition Date: 13-Jun-14
Gated Events: 20000
X Parameter: FSC-H (Linear)

Region	Events	% Gated	% [illegible]
R2	4	0.02	

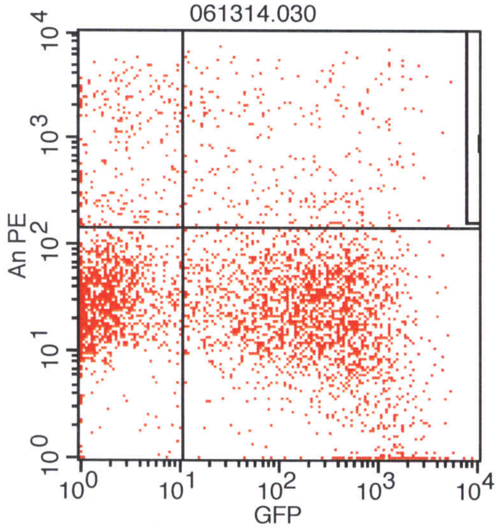

File: 061314.030
Sample ID:
Acquisition Date: 13-Jun-14
Gated Events: 19996
X Parameter: GFP (Log)
Quad Location: 10, 30

Log Data Units: Linear Values
Patient ID:
Gate: G1
Total Events: 20000
Y Parameter: 7AAD (Log)

Quad	Events	% Gated	% Total	X Mean	X Geo Mean	Y Mean	Y Geo Mean
UL	613	3.07	3.06	3.30	2.57	231.44	167.88
UR	585	2.93	2.93	302.66	88.23	217.93	147.65
LL	6388	31.95	31.94	2.54	2.06	5.74	4.64
LR	12410	62.06	62.05	538.56	231.55	3.12	2.06

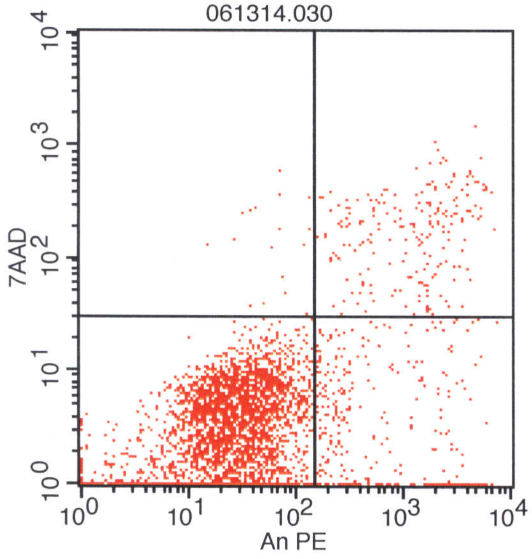

File: 061314.030
Sample ID:
Acquisition Date: 13-Jun-14
Gated Events: 19996
X Parameter: GFP (Log)
Quad Location: 11, 145

Log Data Units: Linear Values
Patient ID:
Gate: G1
Total Events: 20000
Y Parameter: An PE (Log)

Quad	Events	% Gated	% Total	X Mean	X Geo Mean	Y Mean	Y Geo Mean
UL	1229	6.15	6.14	3.43	2.69	1565.21	880.32
UR	1539	7.70	7.70	439.23	136.58	1494.71	796.55
LL	5847	29.24	29.23	2.53	2.03	36.00	26.85
LR	11381	56.92	56.90	543.35	241.56	29.47	17.45

File: 061314.030
Sample ID:
Acquisition Date: 13-Jun-14
Gated Events: 19996
X Parameter: An PE (Log)
Quad Location: 149, 30

Log Data Units: Linear Values
Patient ID:
Gate: G1
Total Events: 20000
Y Parameter: 7AAD (Log)

Quad	Events	% Gated	% Total	X Mean	X Geo Mean	Y Mean	Y Geo Mean
UL	118	0.59	0.59	82.17	66.33	158.98	114.30
UR	1075	5.38	5.38	1725.89	1111.57	232.98	164.61
LL	17141	85.72	85.70	31.55	20.11	3.86	2.69
LR	1662	8.31	8.31	1422.44	713.51	5.66	2.99

[illegible]

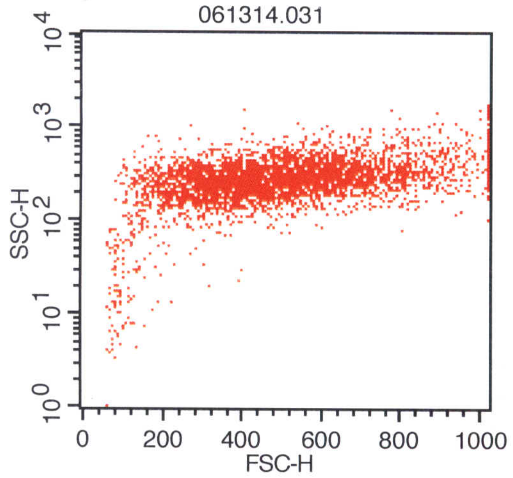

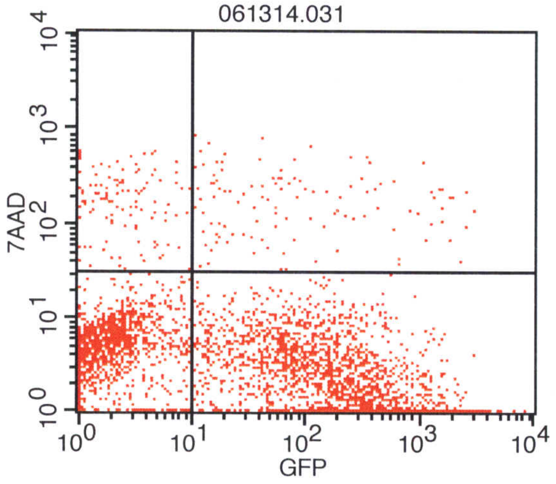

File: 061314.031
Sample ID:
Acquisition Date: 13-Jun-14
Gated Events: 20000
X Parameter: FSC-H (Linear)

Region	Events	% Gated	%
R2	0	0.00	

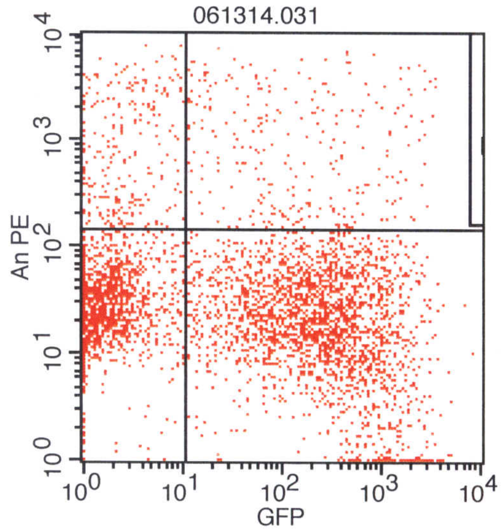

File: 061314.031
Sample ID:
Acquisition Date: 13-Jun-14
Gated Events: 20000
X Parameter: GFP (Log)
Quad Location: 10, 30

Log Data Units: Linear Values
Patient ID:
Gate: G1
Total Events: 20000
Y Parameter: 7AAD (Log)

Quad	Events	% Gated	% Total	X Mean	X Geo Mean	Y Mean	Y Geo Mean
UL	581	2.90	2.90	3.23	2.56	221.52	165.01
UR	588	2.94	2.94	346.79	90.67	208.97	154.96
LL	6231	31.16	31.16	2.48	2.02	5.80	4.65
LR	12600	63.00	63.00	528.09	231.23	3.01	2.02

File: 061314.031
Sample ID:
Acquisition Date: 13-Jun-14
Gated Events: 20000
X Parameter: GFP (Log)
Quad Location: 11, 145

Log Data Units: Linear Values
Patient ID:
Gate: G1
Total Events: 20000
Y Parameter: An PE (Log)

Quad	Events	% Gated	% Total	X Mean	X Geo Mean	Y Mean	Y Geo Mean
UL	1173	5.87	5.87	3.56	2.79	1545.44	900.96
UR	1415	7.07	7.07	466.77	147.83	1514.40	811.86
LL	5728	28.64	28.64	2.46	1.98	34.85	25.89
LR	11684	58.42	58.42	530.33	238.44	29.34	17.22

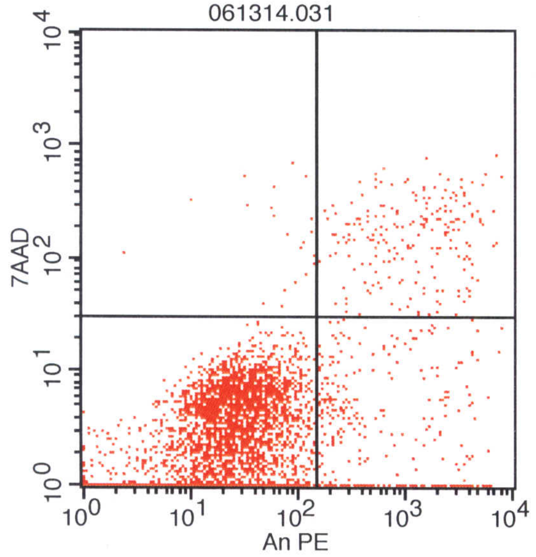

File: 061314.031
Sample ID:
Acquisition Date: 13-Jun-14
Gated Events: 20000
X Parameter: An PE (Log)
Quad Location: 149, 30

Log Data Units: Linear Values
Patient ID:
Gate: G1
Total Events: 20000
Y Parameter: 7AAD (Log)

Quad	Events	% Gated	% Total	X Mean	X Geo Mean	Y Mean	Y Geo Mean
UL	115	0.57	0.57	84.53	71.04	160.52	113.21
UR	1051	5.25	5.25	1729.35	1126.52	221.72	166.82
LL	17321	86.61	86.61	30.96	19.58	3.78	2.63
LR	1513	7.56	7.56	1410.84	720.25	5.69	3.06

87P Syn Q72 9V

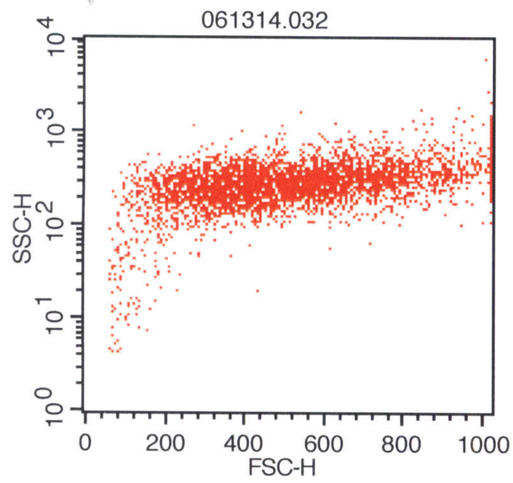

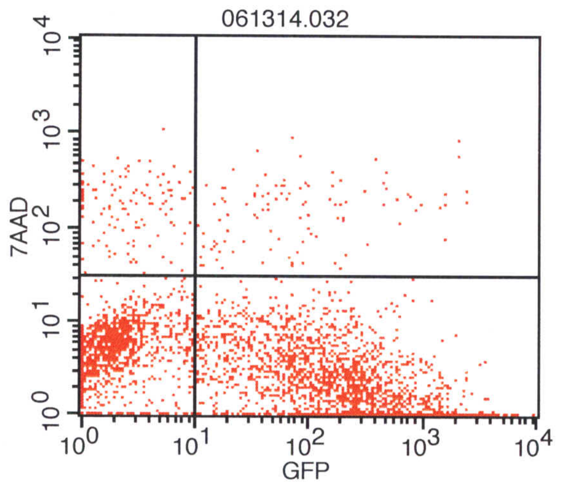

File: 061314.032
Sample ID:
Acquisition Date: 13-Jun-14
Gated Events: 20000
X Parameter: FSC-H (Linear)

Region	Events	% Gated	%
R2	1	0.01	

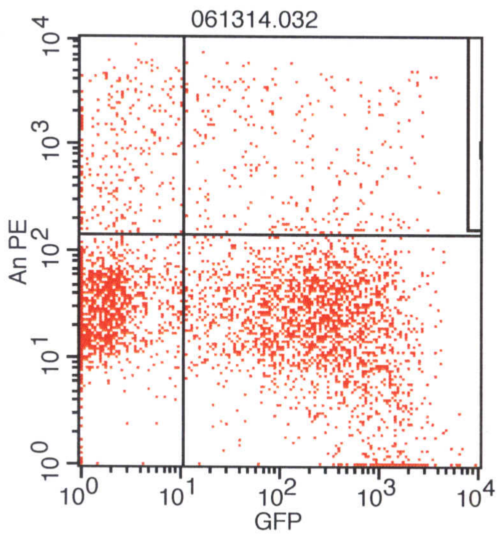

File: 061314.032
Sample ID:
Acquisition Date: 13-Jun-14
Gated Events: 19999
X Parameter: GFP (Log)
Quad Location: 10, 30

Log Data Units: Linear Values
Patient ID:
Gate: G1
Total Events: 20000
Y Parameter: 7AAD (Log)

Quad	Events	% Gated	% Total	X Mean	X Geo Mean	Y Mean	Y Geo Mean
UL	647	3.24	3.23	3.32	2.63	204.12	156.85
UR	571	2.86	2.85	335.98	85.43	189.63	135.86
LL	6283	31.42	31.41	2.48	2.01	5.69	4.60
LR	12498	62.49	62.49	536.80	232.03	3.07	2.04

File: 061314.032
Sample ID:
Acquisition Date: 13-Jun-14
Gated Events: 19999
X Parameter: GFP (Log)
Quad Location: 11, 145

Log Data Units: Linear Values
Patient ID:
Gate: G1
Total Events: 20000
Y Parameter: An PE (Log)

Quad	Events	% Gated	% Total	X Mean	X Geo Mean	Y Mean	Y Geo Mean
UL	1235	6.18	6.17	3.38	2.67	1479.20	864.55
UR	1436	7.18	7.18	454.04	143.94	1426.41	752.23
LL	5789	28.95	28.95	2.51	2.00	35.20	26.35
LR	11539	57.70	57.69	541.45	240.38	29.89	17.51

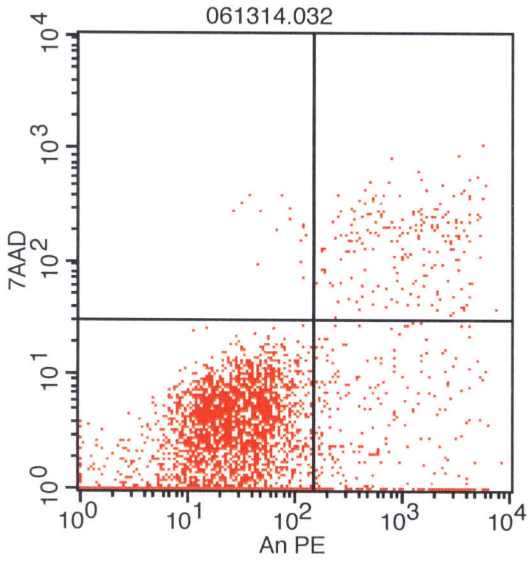

File: 061314.032
Sample ID:
Acquisition Date: 13-Jun-14
Gated Events: 19999
X Parameter: An PE (Log)
Quad Location: 149, 30

Log Data Units: Linear Values
Patient ID:
Gate: G1
Total Events: 20000
Y Parameter: 7AAD (Log)

Quad	Events	% Gated	% Total	X Mean	X Geo Mean	Y Mean	Y Geo Mean
UL	120	0.60	0.60	75.81	61.19	165.24	116.88
UR	1092	5.46	5.46	1609.63	1017.92	201.77	151.64
LL	17232	86.16	86.16	31.51	19.97	3.79	2.65
LR	1555	7.78	7.78	1359.42	696.69	5.82	3.10

[illegible]

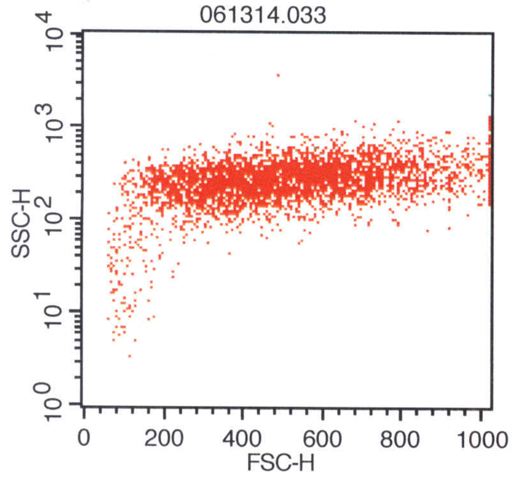

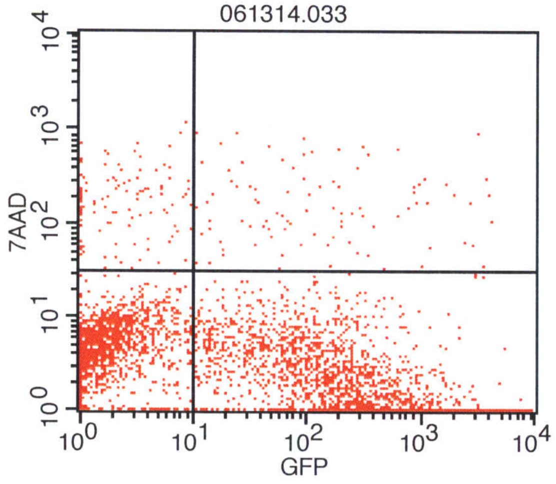

File: 061314.033
Sample ID:
Acquisition Date: 13-Jun-14
Gated Events: 20000
X Parameter: FSC-H (Linear)

Region	Events	% Gated	%
R2	4	0.02	

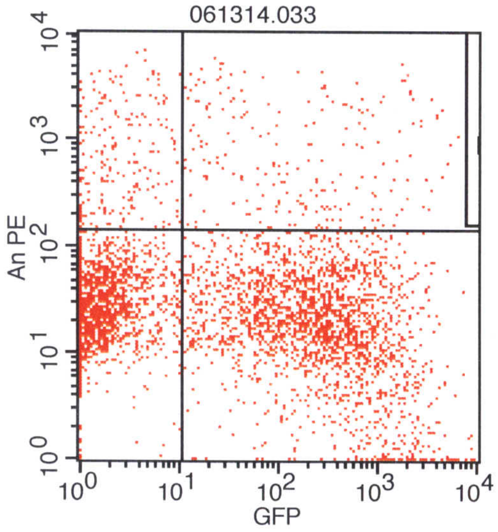

File: 061314.033
Sample ID:
Acquisition Date: 13-Jun-14
Gated Events: 19996
X Parameter: GFP (Log)
Quad Location: 10, 30

Log Data Units: Linear Values
Patient ID:
Gate: G1
Total Events: 20000
Y Parameter: 7AAD (Log)

Quad	Events	% Gated	% Total	X Mean	X Geo Mean	Y Mean	Y Geo Mean
UL	567	2.84	2.83	3.09	2.41	231.05	173.07
UR	518	2.59	2.59	474.53	97.84	202.24	142.72
LL	7131	35.66	35.66	2.27	1.85	5.61	4.58
LR	11780	58.91	58.90	610.53	242.00	2.95	1.98

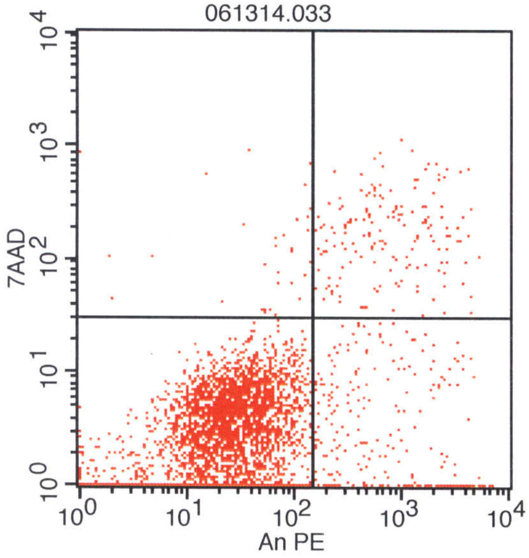

File: 061314.033
Sample ID:
Acquisition Date: 13-Jun-14
Gated Events: 19996
X Parameter: GFP (Log)
Quad Location: 11, 145

Log Data Units: Linear Values
Patient ID:
Gate: G1
Total Events: 20000
Y Parameter: An PE (Log)

Quad	Events	% Gated	% Total	X Mean	X Geo Mean	Y Mean	Y Geo Mean
UL	1061	5.31	5.30	3.28	2.56	1311.36	741.68
UR	1209	6.05	6.04	674.60	169.87	1310.01	728.62
LL	6723	33.62	33.62	2.28	1.83	34.45	26.22
LR	11003	55.03	55.02	601.78	247.11	27.93	16.43

File: 061314.033
Sample ID:
Acquisition Date: 13-Jun-14
Gated Events: 19996
X Parameter: An PE (Log)
Quad Location: 149, 30

Log Data Units: Linear Values
Patient ID:
Gate: G1
Total Events: 20000
Y Parameter: 7AAD (Log)

Quad	Events	% Gated	% Total	X Mean	X Geo Mean	Y Mean	Y Geo Mean
UL	126	0.63	0.63	77.54	55.55	186.69	117.96
UR	956	4.78	4.78	1397.78	906.57	221.92	164.88
LL	17630	88.17	88.15	30.26	19.54	3.83	2.69
LR	1284	6.42	6.42	1272.94	652.34	5.71	3.04

UH141 S1- Q72 gfp

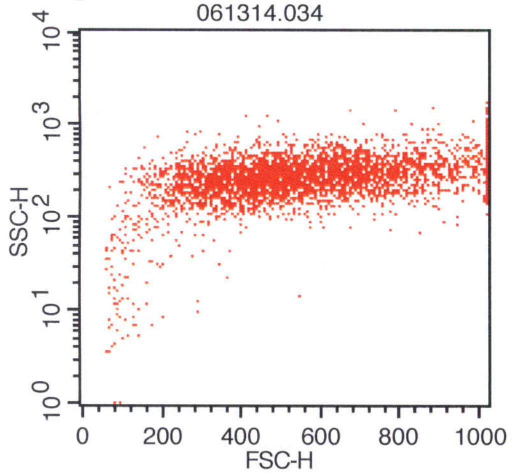

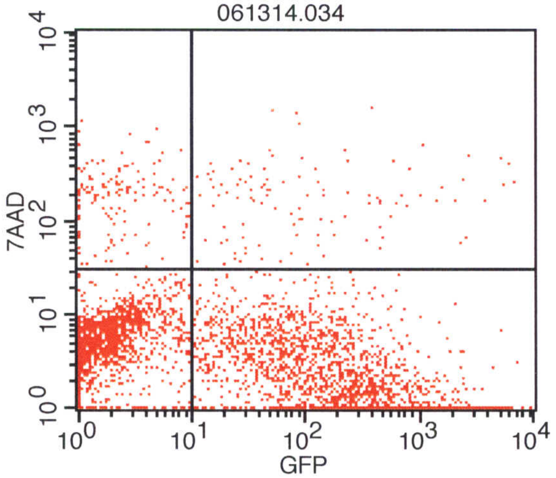

File: 061314.034
Sample ID:
Acquisition Date: 13-Jun-14
Gated Events: 20000
X Parameter: FSC-H (Linear)

Region	Events	% Gated	%
R2	9	0.04	

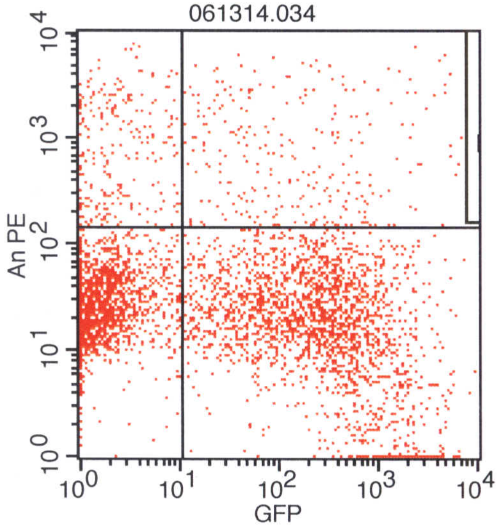

File: 061314.034
Sample ID:
Acquisition Date: 13-Jun-14
Gated Events: 19991
X Parameter: GFP (Log)
Quad Location: 10, 30

Log Data Units: Linear Values
Patient ID:
Gate: G1
Total Events: 20000
Y Parameter: 7AAD (Log)

Quad	Events	% Gated	% Total	X Mean	X Geo Mean	Y Mean	Y Geo Mean
UL	664	3.32	3.32	3.07	2.38	230.03	170.78
UR	580	2.90	2.90	428.91	96.54	219.08	153.60
LL	7174	35.89	35.87	2.30	1.88	5.82	4.76
LR	11573	57.89	57.87	605.14	241.35	3.10	2.05

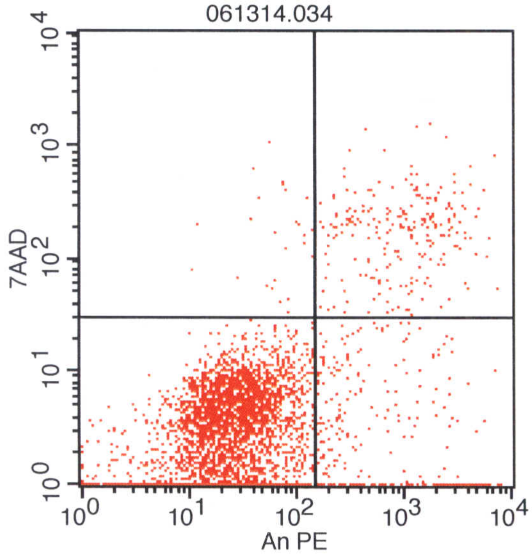

File: 061314.034
Sample ID:
Acquisition Date: 13-Jun-14
Gated Events: 19991
X Parameter: GFP (Log)
Quad Location: 11, 145

Log Data Units: Linear Values
Patient ID:
Gate: G1
Total Events: 20000
Y Parameter: An PE (Log)

Quad	Events	% Gated	% Total	X Mean	X Geo Mean	Y Mean	Y Geo Mean
UL	1177	5.89	5.88	3.20	2.46	1416.34	795.29
UR	1322	6.61	6.61	593.75	161.41	1344.24	759.74
LL	6731	33.67	33.66	2.30	1.86	34.54	26.34
LR	10761	53.83	53.80	600.90	246.36	28.44	16.80

File: 061314.034
Sample ID:
Acquisition Date: 13-Jun-14
Gated Events: 19991
X Parameter: An PE (Log)
Quad Location: 149, 30

Log Data Units: Linear Values
Patient ID:
Gate: G1
Total Events: 20000
Y Parameter: 7AAD (Log)

Quad	Events	% Gated	% Total	X Mean	X Geo Mean	Y Mean	Y Geo Mean
UL	133	0.67	0.66	75.48	59.61	192.74	121.83
UR	1106	5.53	5.53	1543.15	1001.02	229.67	169.56
LL	17394	87.01	86.97	30.68	19.89	4.03	2.83
LR	1358	6.79	6.79	1275.57	658.68	5.64	2.99

UHIHP S77 @ 72 [illegible]

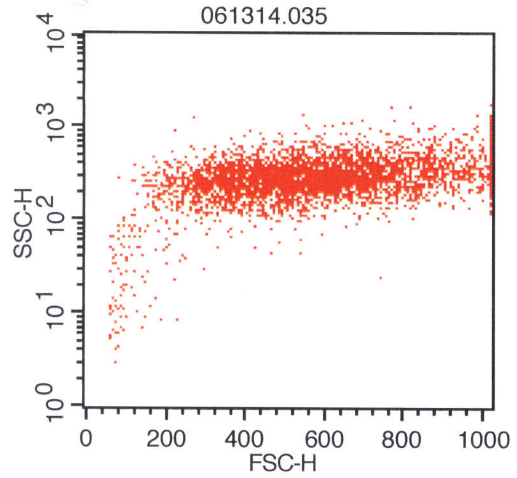

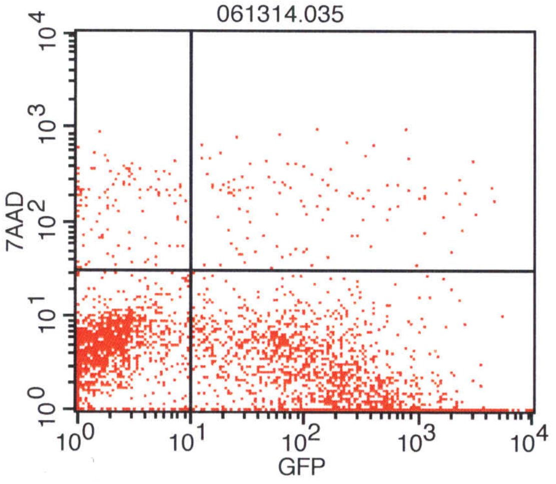

File: 061314.035
Sample ID:
Acquisition Date: 13-Jun-14
Gated Events: 20000
X Parameter: FSC-H (Linear)

Region	Events	% Gated	%
R2	4	0.02	

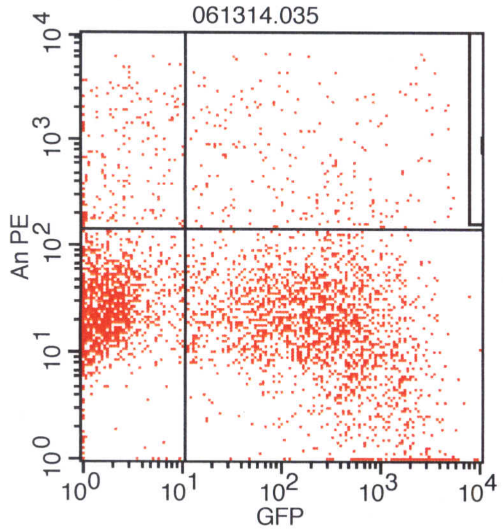

File: 061314.035
Sample ID:
Acquisition Date: 13-Jun-14
Gated Events: 19996
X Parameter: GFP (Log)
Quad Location: 10, 30

Log Data Units: Linear Values
Patient ID:
Gate: G1
Total Events: 20000
Y Parameter: 7AAD (Log)

Quad	Events	% Gated	% Total	X Mean	X Geo Mean	Y Mean	Y Geo Mean
UL	570	2.85	2.85	3.05	2.40	225.96	166.54
UR	538	2.69	2.69	461.77	105.66	213.01	147.72
LL	7333	36.67	36.66	2.31	1.90	5.46	4.47
LR	11555	57.79	57.77	632.18	251.50	2.86	1.91

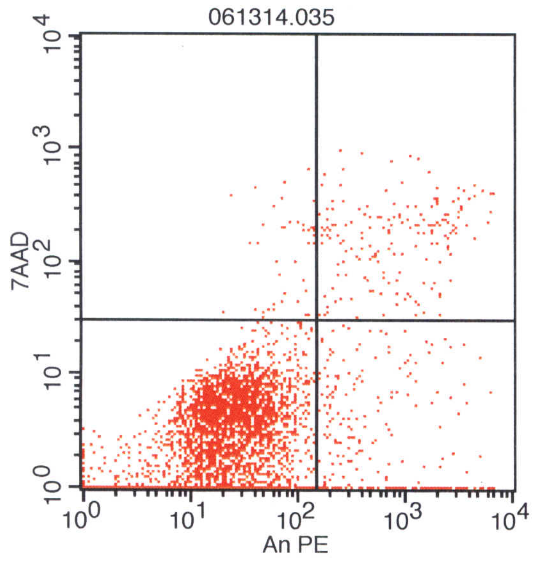

File: 061314.035
Sample ID:
Acquisition Date: 13-Jun-14
Gated Events: 19996
X Parameter: GFP (Log)
Quad Location: 11, 145

Log Data Units: Linear Values
Patient ID:
Gate: G1
Total Events: 20000
Y Parameter: An PE (Log)

Quad	Events	% Gated	% Total	X Mean	X Geo Mean	Y Mean	Y Geo Mean
UL	975	4.88	4.88	3.35	2.59	1378.28	745.40
UR	1117	5.59	5.58	636.00	171.37	1304.01	712.36
LL	7007	35.04	35.03	2.31	1.89	33.04	24.90
LR	10897	54.50	54.48	627.89	256.49	25.82	14.54

File: 061314.035
Sample ID:
Acquisition Date: 13-Jun-14
Gated Events: 19996
X Parameter: An PE (Log)
Quad Location: 149, 30

Log Data Units: Linear Values
Patient ID:
Gate: G1
Total Events: 20000
Y Parameter: 7AAD (Log)

Quad	Events	% Gated	% Total	X Mean	X Geo Mean	Y Mean	Y Geo Mean
UL	206	1.03	1.03	78.64	62.51	168.15	121.09
UR	899	4.50	4.50	1459.72	891.61	232.11	167.70
LL	17730	88.67	88.65	28.28	17.76	3.78	2.64
LR	1161	5.81	5.80	1277.70	649.59	5.37	2.85
