# Supplementary material for: Bifunctional Anti-Non-Amyloid Component α-Synuclein Nanobodies Are Protective In Situ
Source: PLoS One. 2016 Nov 8;11(11):e0165964. doi: 10.1371/journal.pone.0165964 (PMC5100967; doi:10.1371/journal.pone.0165964)
Supplement: S4 Fig — (PDF) [file pone.0165964.s004.pdf]

Feb 20 2014

ST14A - settings

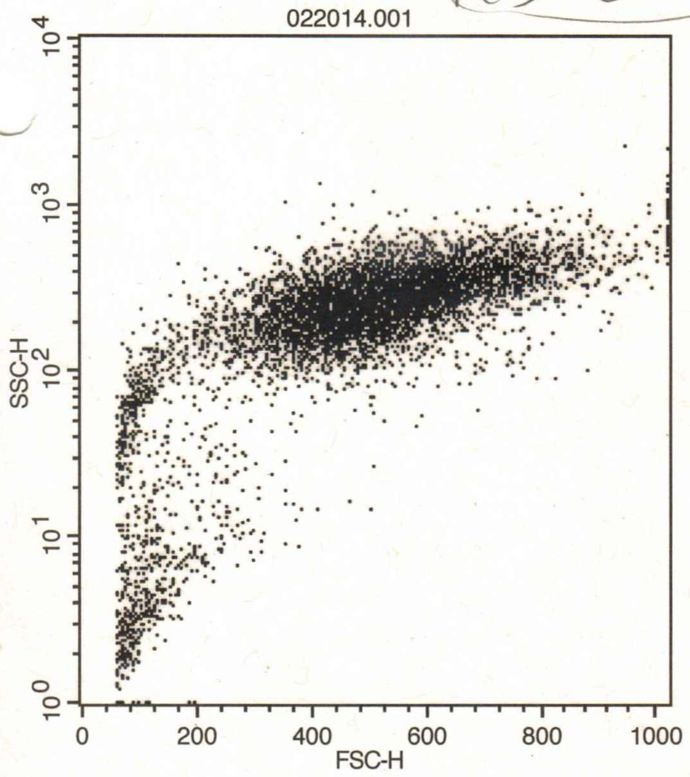

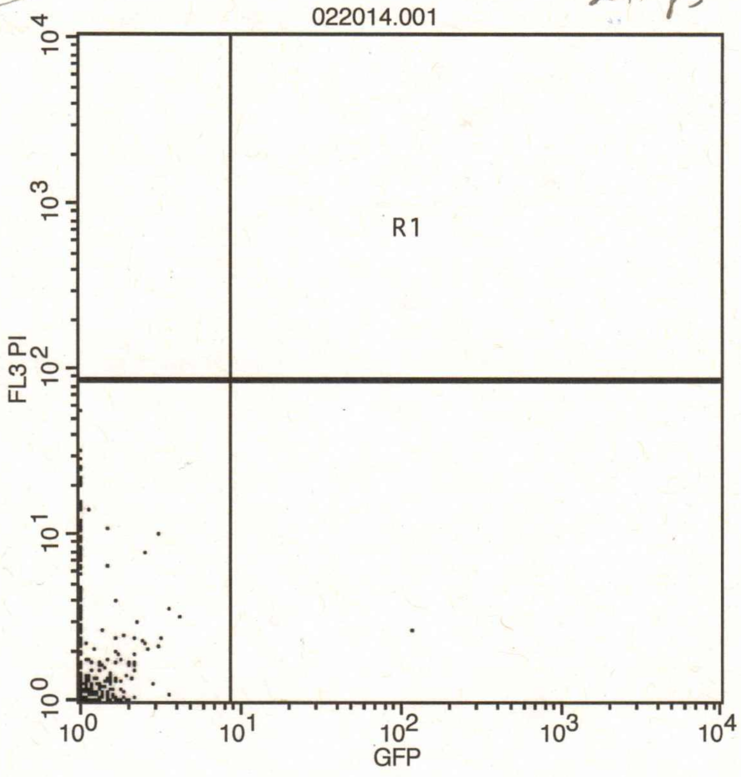

Quadrant Statistics

File: 022014.001 | Log Data Units: Linear Values
Sample ID: | Patient ID:
Tube: Untitled | Panel: Untitled Acquisition Tube List
Acquisition Date: 20-Feb-14 | Gate: No Gate
Gated Events: 6690 | Total Events: 6690
X Parameter: GFP (Log) | Y Parameter: FL3 PI (Log)
Quad Location: 9, 83

Quad	Events	% Gated	% Total	X Mean	X Geo Mean	Y Mean	Y Geo Mean
UL	0	0.00	0.00	***	***	***	***
UR	0	0.00	0.00	***	***	***	***
LL	6689	99.99	99.99	1.01	1.01	1.12	1.05
LR	1	0.01	0.01	119.71	119.71	2.64	2.64

GFP settings

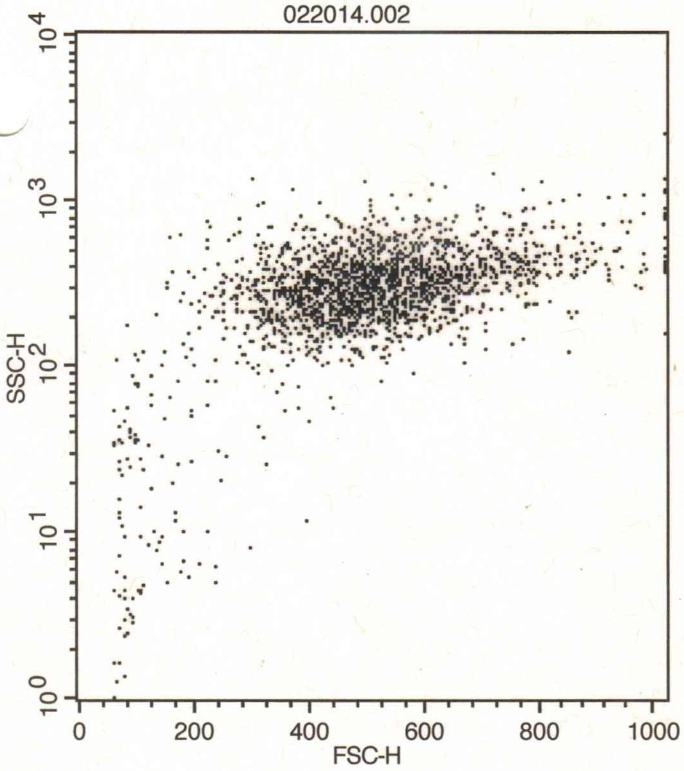

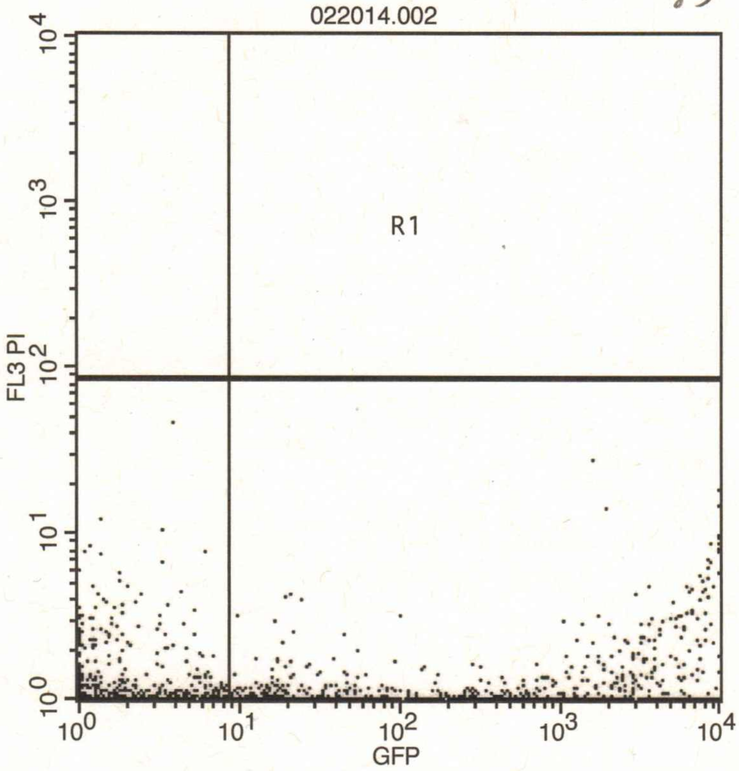

Quadrant Statistics

File: 022014.002
Sample ID:
Tube: Untitled
Acquisition Date: 20-Feb-14
Gated Events: 2100
X Parameter: GFP (Log)
Quad Location: 9, 83

Log Data Units: Linear Values
Patient ID:
Panel: Untitled Acquisition Tube List
Gate: No Gate
Total Events: 2100
Y Parameter: FL3 PI (Log)

Quad	Events	% Gated	% Total	X Mean	X Geo Mean	Y Mean	Y Geo Mean
UL	0	0.00	0.00	***	***	***	***
UR	0	0.00	0.00	***	***	***	***
LL	996	47.43	47.43	2.33	1.80	1.29	1.15
LR	1104	52.57	52.57	1558.96	330.03	1.33	1.16

STMA Settings

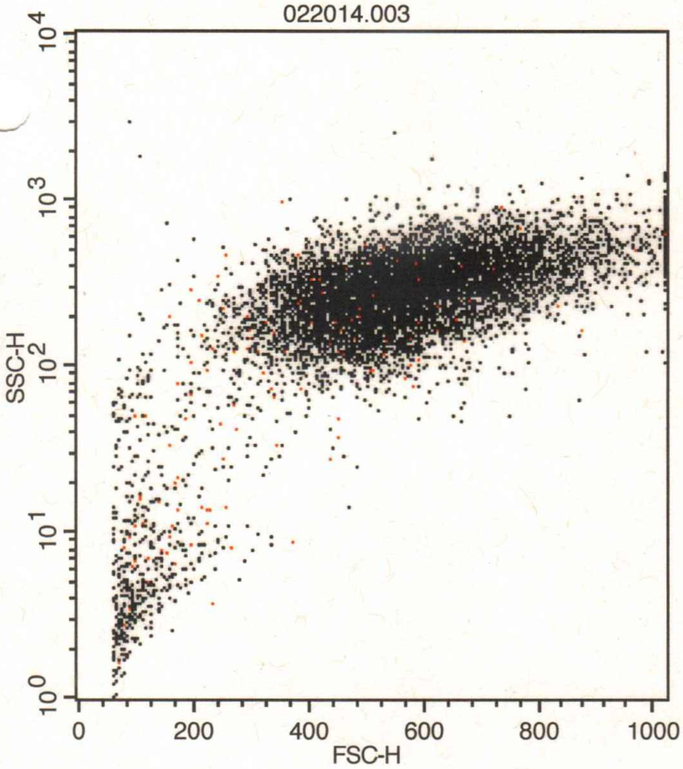

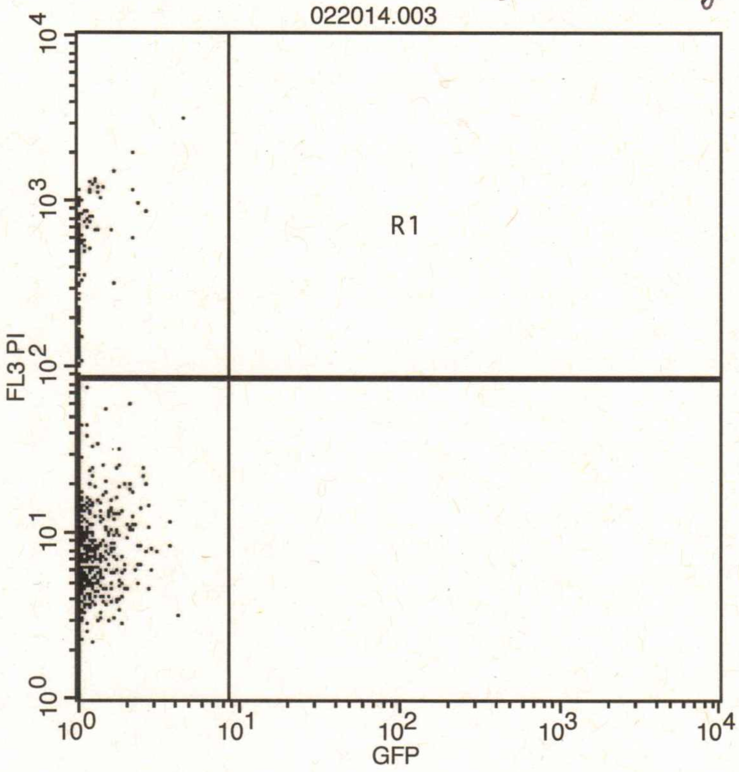

Quadrant Statistics

File: 022014.003
Sample ID:
Tube: Untitled
Acquisition Date: 20-Feb-14
Gated Events: 10000
X Parameter: GFP (Log)
Quad Location: 9, 83

Log Data Units: Linear Values
Patient ID:
Panel: Untitled Acquisition Tube List
Gate: No Gate
Total Events: 10000
Y Parameter: FL3 PI (Log)

Quad	Events	% Gated	% Total	X Mean	X Geo Mean	Y Mean	Y Geo Mean
UL	154	1.54	1.54	1.11	1.08	507.69	360.81
UR	0	0.00	0.00	***	***	***	***
LL	9846	98.46	98.46	1.01	1.01	5.61	4.46
LR	0	0.00	0.00	***	***	***	***

ST14A Sc Hi-ys

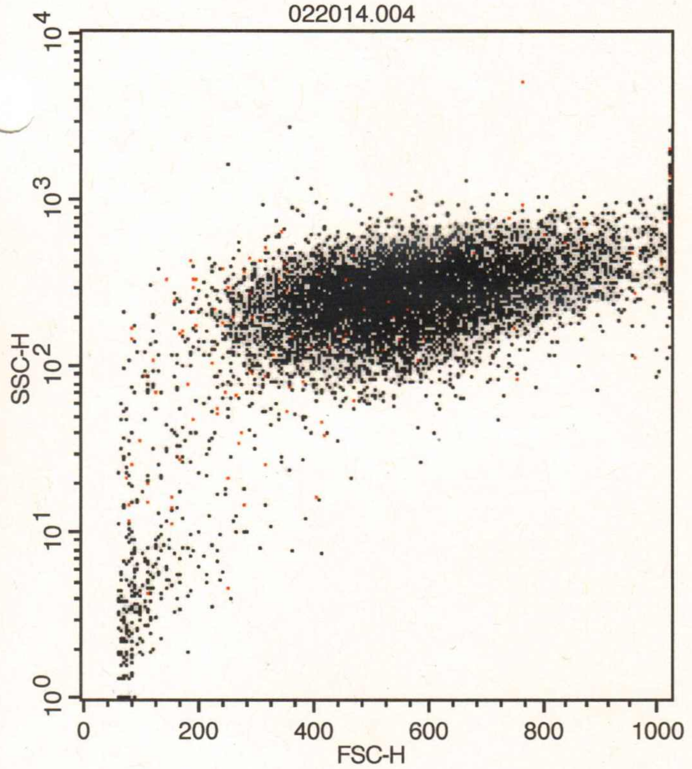

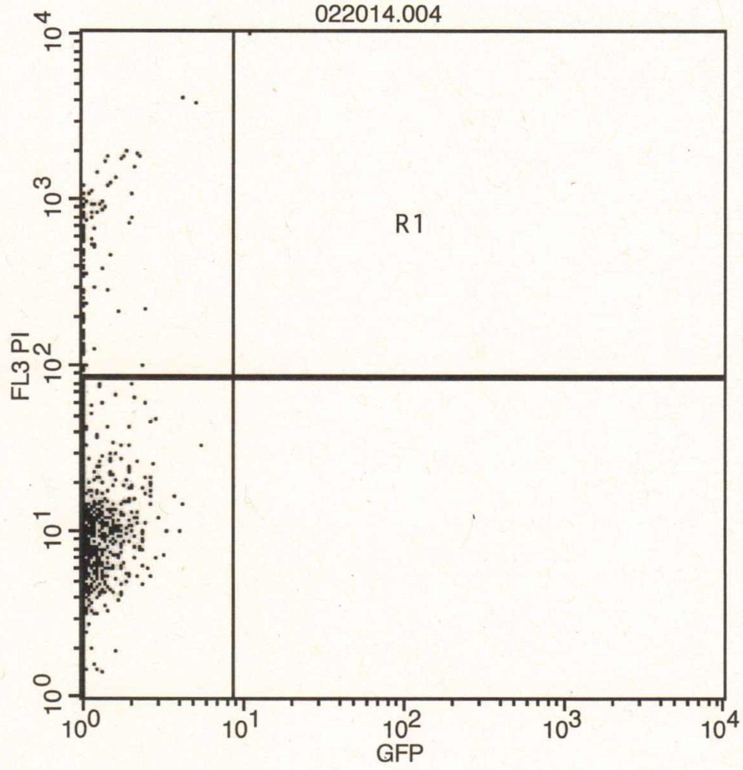

Quadrant Statistics

File: 022014.004	Log Data Units: Linear Values
Sample ID:	Patient ID:
Tube: Untitled	Panel: Untitled Acquisition Tube List
Acquisition Date: 20-Feb-14	Gate: No Gate
Gated Events: 10000	Total Events: 10000
X Parameter: GFP (Log)	Y Parameter: FL3 PI (Log)
Quad Location: 9, 83	

Quad	Events	% Gated	% Total	X Mean	X Geo Mean	Y Mean	Y Geo Mean
UL	133	1.33	1.33	1.21	1.15	620.22	418.20
UR	1	0.01	0.01	10.75	10.75	9910.46	9910.46
LL	9866	98.66	98.66	1.02	1.02	6.30	5.04
LR	0	0.00	0.00	***	***	***	***

S#14A - settings

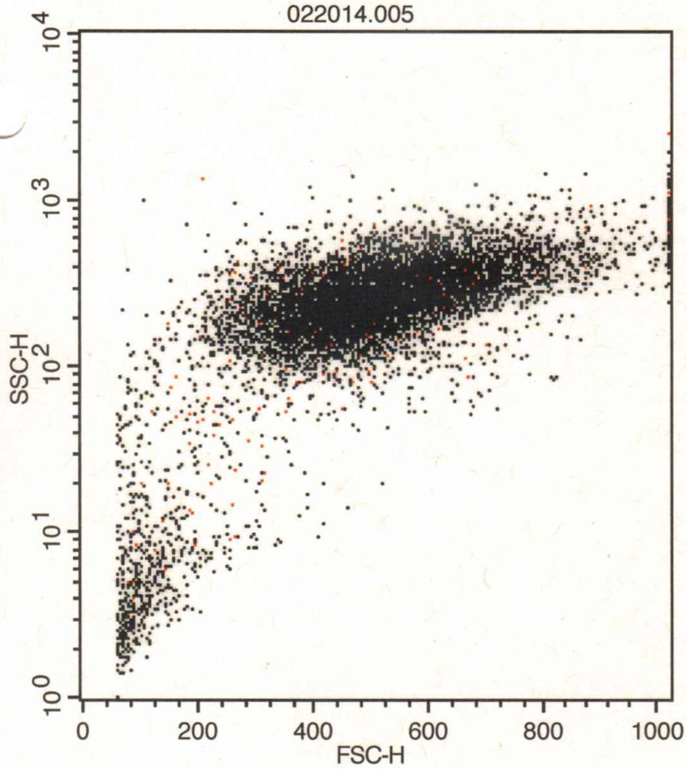

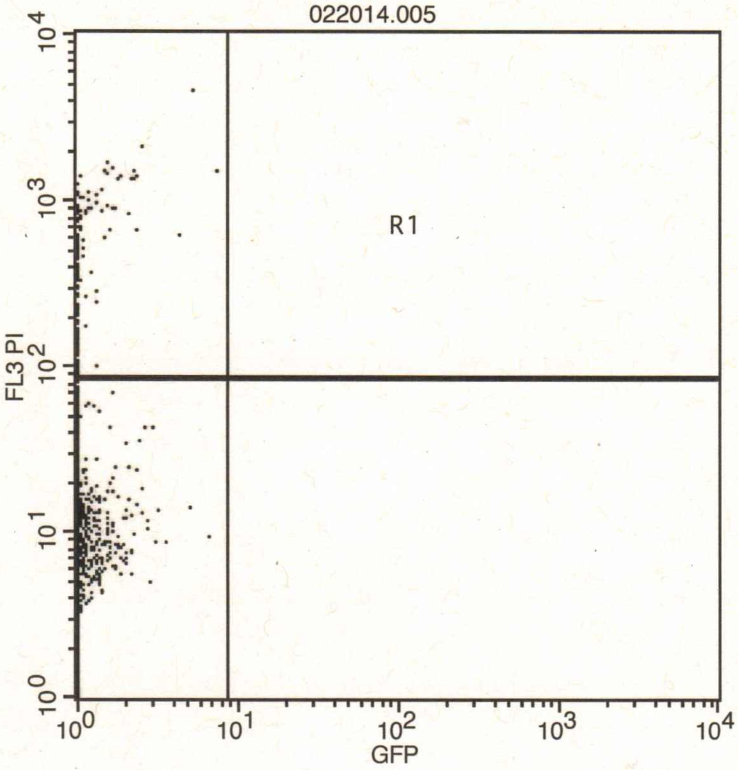

Quadrant Statistics

File: 022014.005	Log Data Units: Linear Values
Sample ID:	Patient ID:
Tube: Untitled	Panel: Untitled Acquisition Tube List
Acquisition Date: 20-Feb-14	Gate: No Gate
Gated Events: 10000	Total Events: 10000
X Parameter: GFP (Log)	Y Parameter: FL3 PI (Log)
Quad Location: 9, 83	

Quad	Events	% Gated	% Total	X Mean	X Geo Mean	Y Mean	Y Geo Mean
UL	158	1.58	1.58	1.22	1.14	583.01	413.26
UR	0	0.00	0.00	***	***	***	***
LL	9842	98.42	98.42	1.01	1.01	6.45	5.23
LR	0	0.00	0.00	***	***	***	***

Empty Vector / GFP

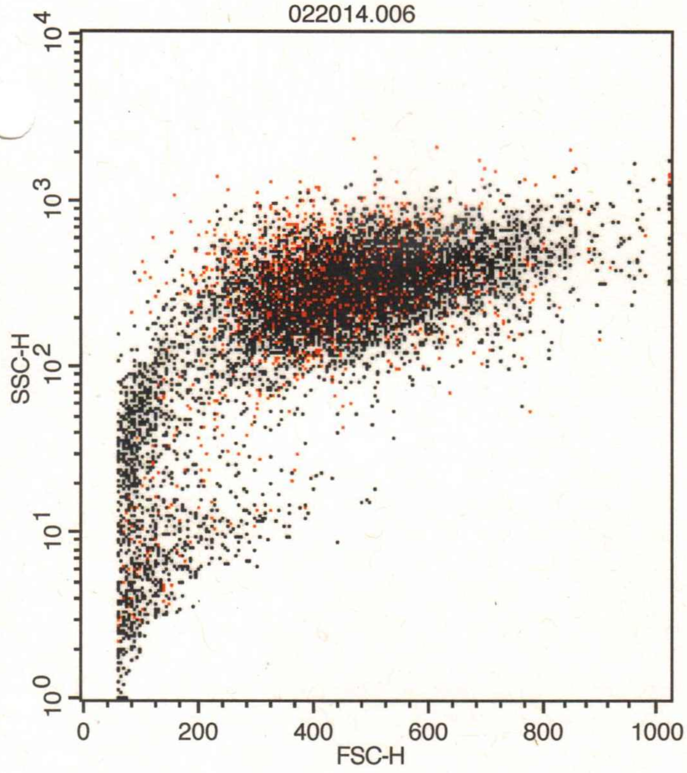

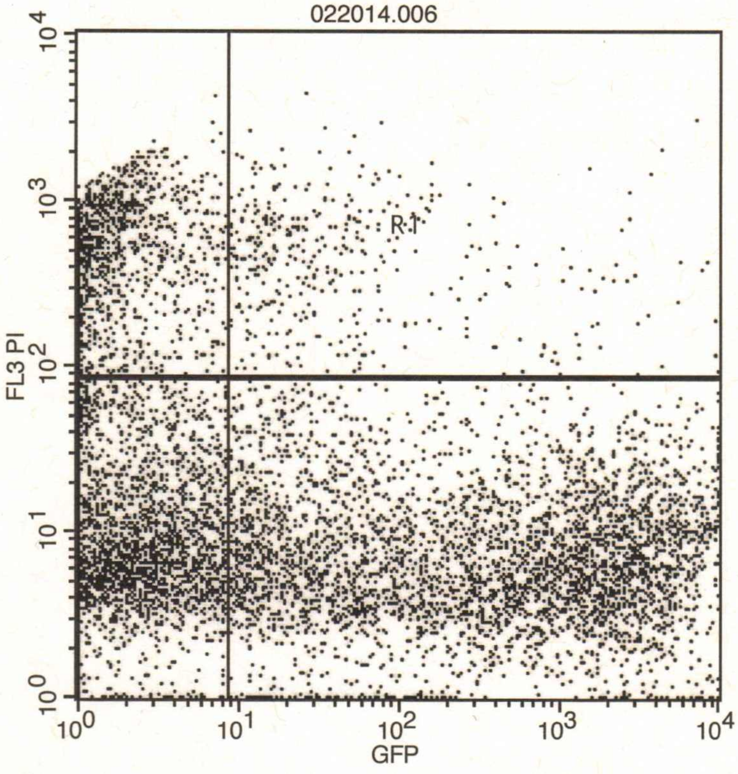

Quadrant Statistics

File: 022014.006
Sample ID:
Tube: Untitled
Acquisition Date: 20-Feb-14
Gated Events: 10000
X Parameter: GFP (Log)
Quad Location: 9, 83

Log Data Units: Linear Values
Patient ID:
Panel: Untitled Acquisition Tube List
Gate: No Gate
Total Events: 10000
Y Parameter: FL3 PI (Log)

Quad	Events	% Gated	% Total	X Mean	X Geo Mean	Y Mean	Y Geo Mean
UL	1302	13.02	13.02	2.06	1.69	520.36	374.88
UR	406	4.06	4.06	396.34	53.01	563.95	405.26
LL	4308	43.08	43.08	2.15	1.66	14.12	7.90
LR	3984	39.84	39.84	1383.06	301.20	10.41	6.77

Empty Vector / GFP

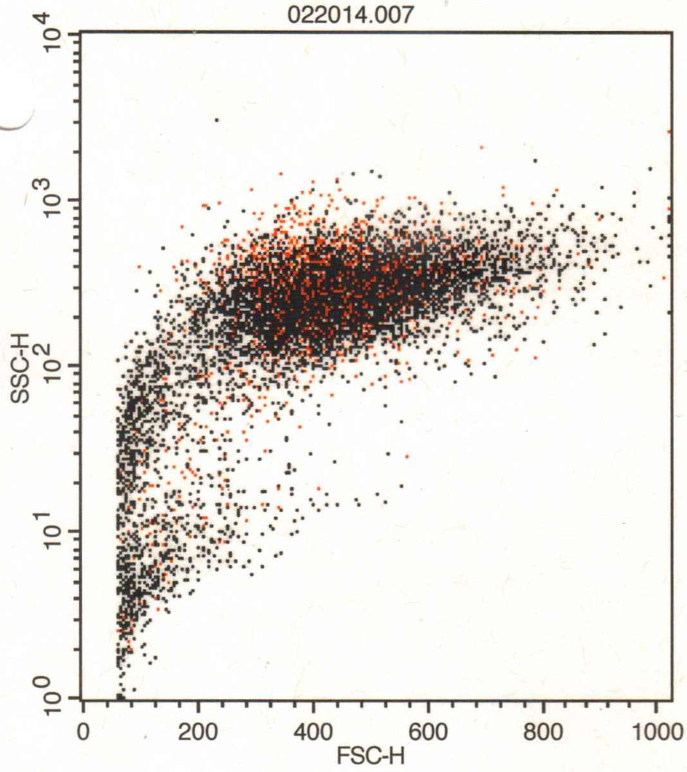

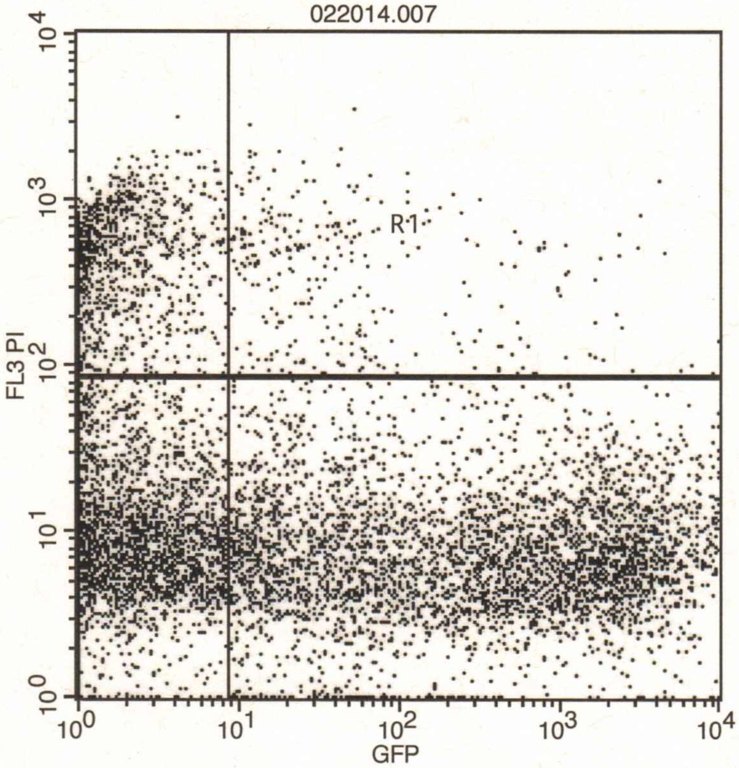

Quadrant Statistics

File: 022014.007
Sample ID:
Tube: Untitled
Acquisition Date: 20-Feb-14
Gated Events: 10000
X Parameter: GFP (Log)
Quad Location: 9, 83

Log Data Units: Linear Values
Patient ID:
Panel: Untitled Acquisition Tube List
Gate: No Gate
Total Events: 10000
Y Parameter: FL3 PI (Log)

Quad	Events	% Gated	% Total	X Mean	X Geo Mean	Y Mean	Y Geo Mean
UL	1234	12.34	12.34	2.04	1.67	483.89	369.39
UR	315	3.15	3.15	327.98	52.68	490.72	339.96
LL	4442	44.42	44.42	2.09	1.62	13.87	8.17
LR	4009	40.09	40.09	1206.70	275.03	10.04	6.89

Empty Vector / GFP

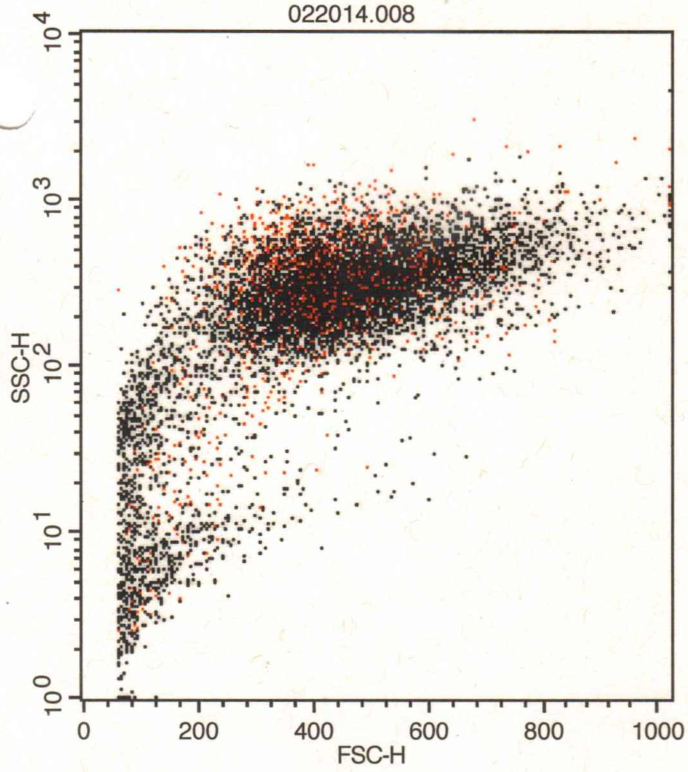

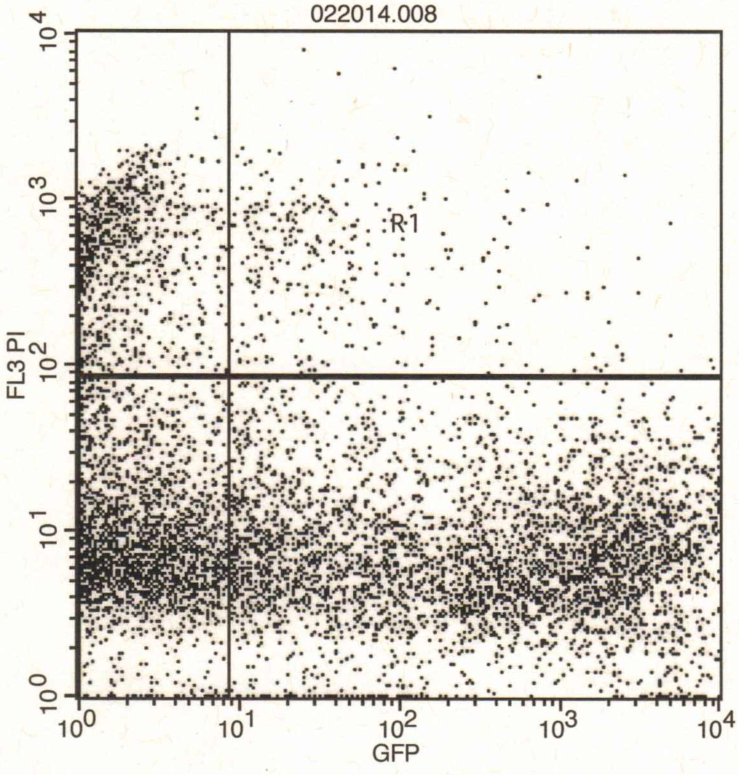

Quadrant Statistics

File: 022014.008
Sample ID:
Tube: Untitled
Acquisition Date: 20-Feb-14
Gated Events: 10000
X Parameter: GFP (Log)
Quad Location: 9, 83

Log Data Units: Linear Values
Patient ID:
Panel: Untitled Acquisition Tube List
Gate: No Gate
Total Events: 10000
Y Parameter: FL3 PI (Log)

Quad	Events	% Gated	% Total	X Mean	X Geo Mean	Y Mean	Y Geo Mean
UL	1151	11.51	11.51	2.01	1.67	514.88	369.41
UR	325	3.25	3.25	293.63	52.80	615.75	408.09
LL	4334	43.34	43.34	2.14	1.66	13.09	7.75
LR	4190	41.90	41.90	1395.80	301.88	10.31	6.78

5787 PEST / GFP

022014.009

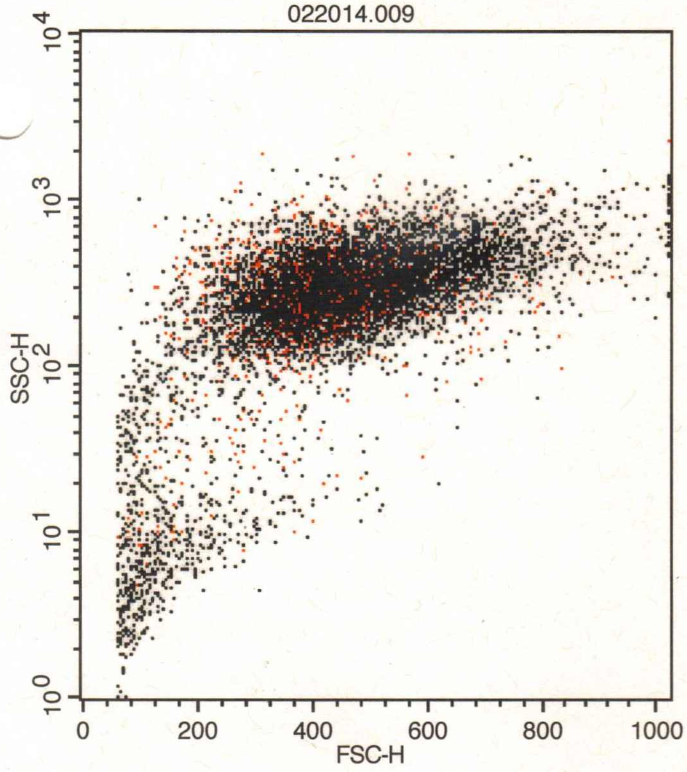

022014.009

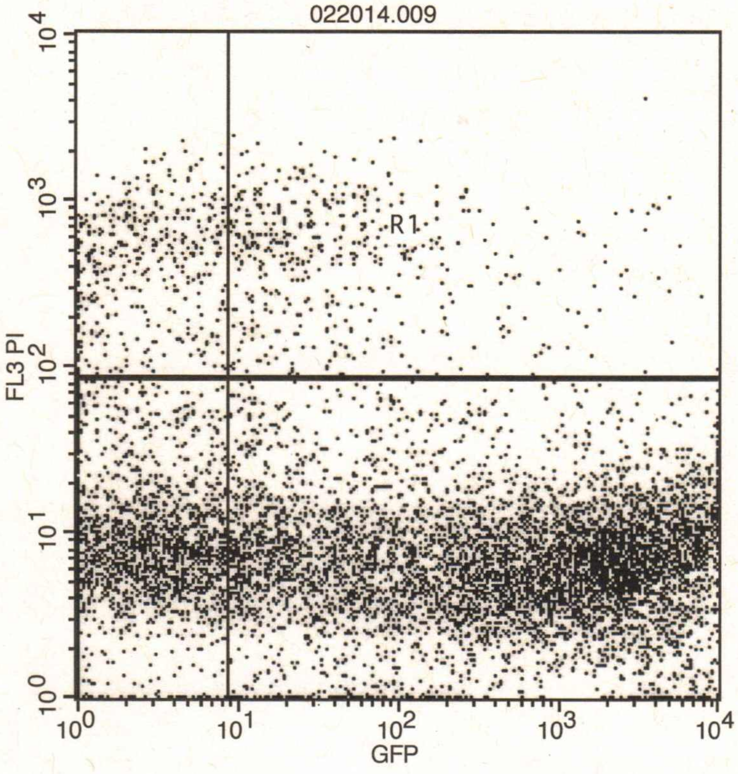

Quadrant Statistics

File: 022014.009	Log Data Units: Linear Values
Sample ID:	Patient ID:
Tube: Untitled	Panel: Untitled Acquisition Tube List
Acquisition Date: 20-Feb-14	Gate: No Gate
Gated Events: 10000	Total Events: 10000
X Parameter: GFP (Log)	Y Parameter: FL3 PI (Log)
Quad Location: 9, 83	

Quad	Events	% Gated	% Total	X Mean	X Geo Mean	Y Mean	Y Geo Mean
UL	467	4.67	4.67	3.14	2.44	510.10	387.03
UR	482	4.82	4.82	288.17	50.26	582.38	432.20
LL	2562	25.62	25.62	2.74	2.10	11.52	7.41
LR	6489	64.89	64.89	1836.35	482.67	8.84	6.31

Syn87PEST / GFP

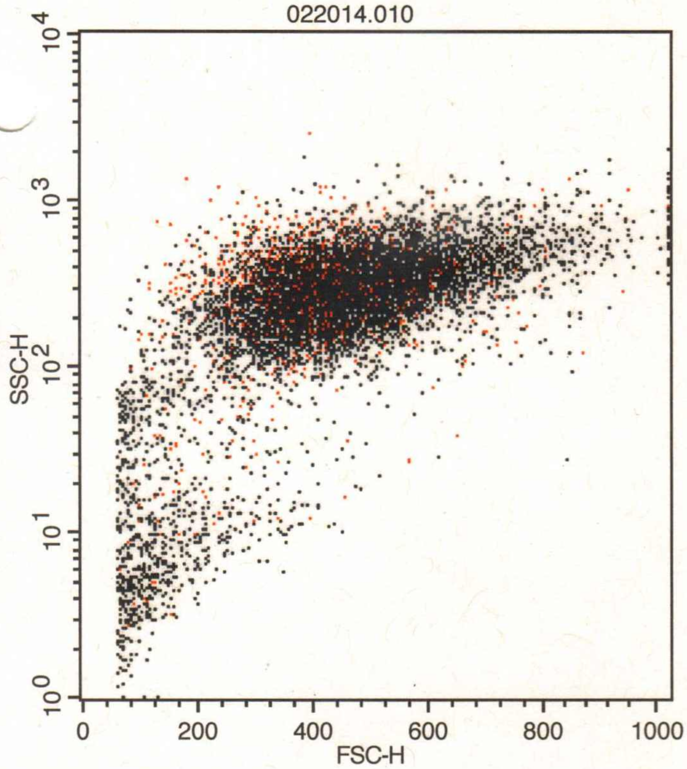

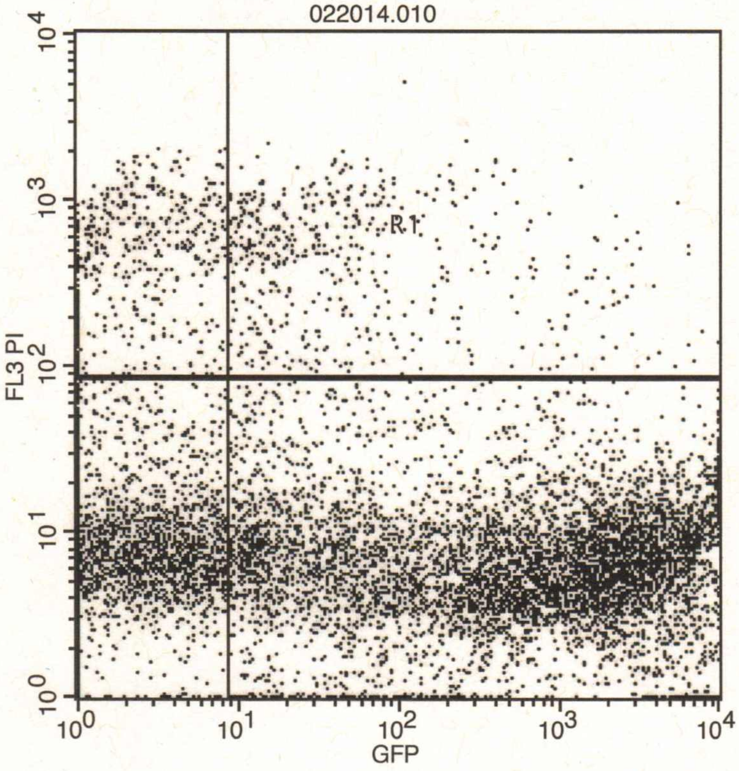

Quadrant Statistics

File: 022014.010
Sample ID:
Tube: Untitled
Acquisition Date: 20-Feb-14
Gated Events: 10000
X Parameter: GFP (Log)
Quad Location: 9, 83

Log Data Units: Linear Values
Patient ID:
Panel: Untitled Acquisition Tube List
Gate: No Gate
Total Events: 10000
Y Parameter: FL3 PI (Log)

Quad	Events	% Gated	% Total	X Mean	X Geo Mean	Y Mean	Y Geo Mean
UL	476	4.76	4.76	3.05	2.40	572.39	424.00
UR	515	5.15	5.15	345.20	55.32	564.48	413.62
LL	2655	26.55	26.55	2.67	2.04	11.23	7.34
LR	6354	63.54	63.54	1842.96	488.99	9.01	6.29

S7187 PEST / GFP

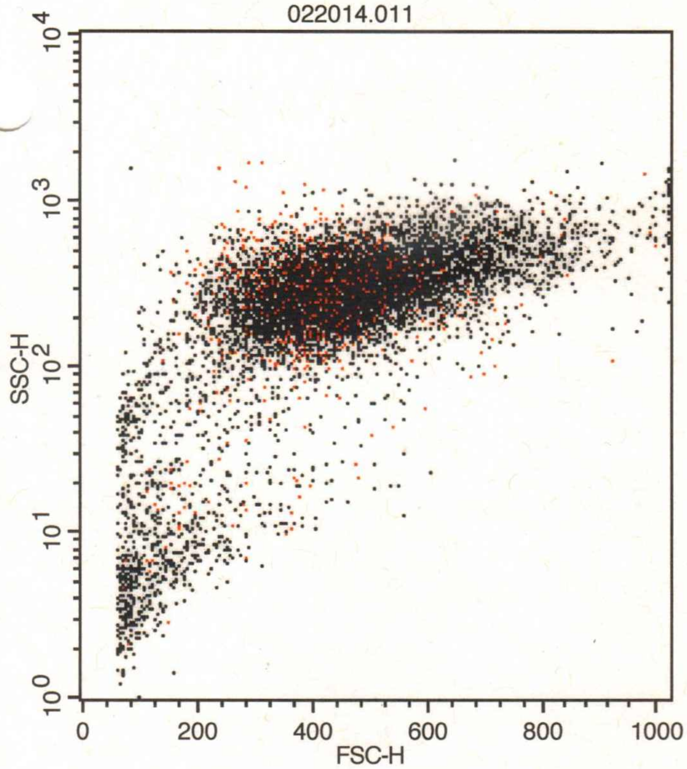

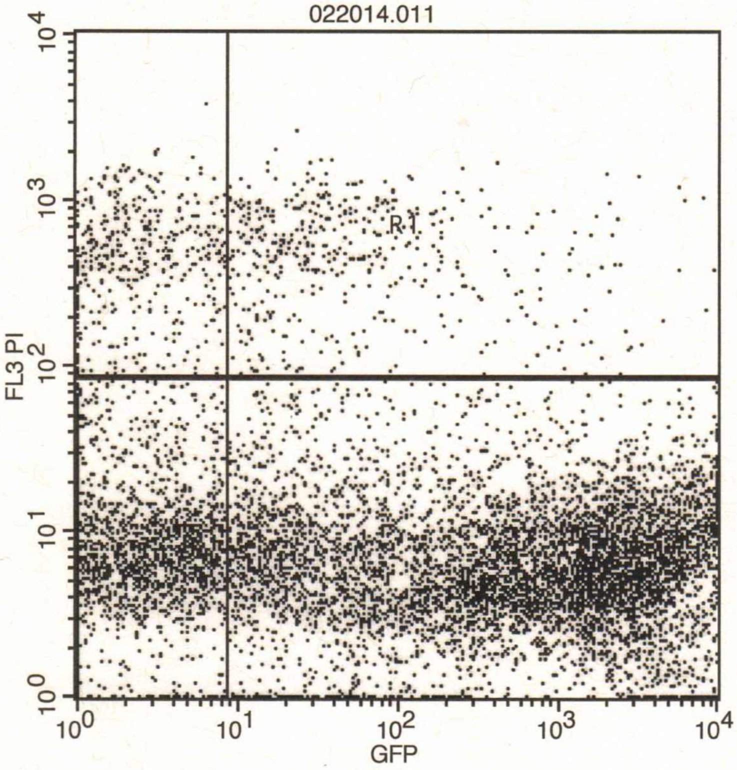

Quadrant Statistics

File: 022014.011	Log Data Units: Linear Values
Sample ID:	Patient ID:
Tube: Untitled	Panel: Untitled Acquisition Tube List
Acquisition Date: 20-Feb-14	Gate: No Gate
Gated Events: 10000	Total Events: 10000
X Parameter: GFP (Log)	Y Parameter: FL3 PI (Log)
Quad Location: 9, 83	

Quad	Events	% Gated	% Total	X Mean	X Geo Mean	Y Mean	Y Geo Mean
UL	481	4.81	4.81	2.94	2.32	531.45	408.70
UR	463	4.63	4.63	348.44	60.31	555.54	427.32
LL	2619	26.19	26.19	2.70	2.05	10.99	6.99
LR	6437	64.37	64.37	1828.74	494.20	8.66	6.05

VHH PEST / GFP

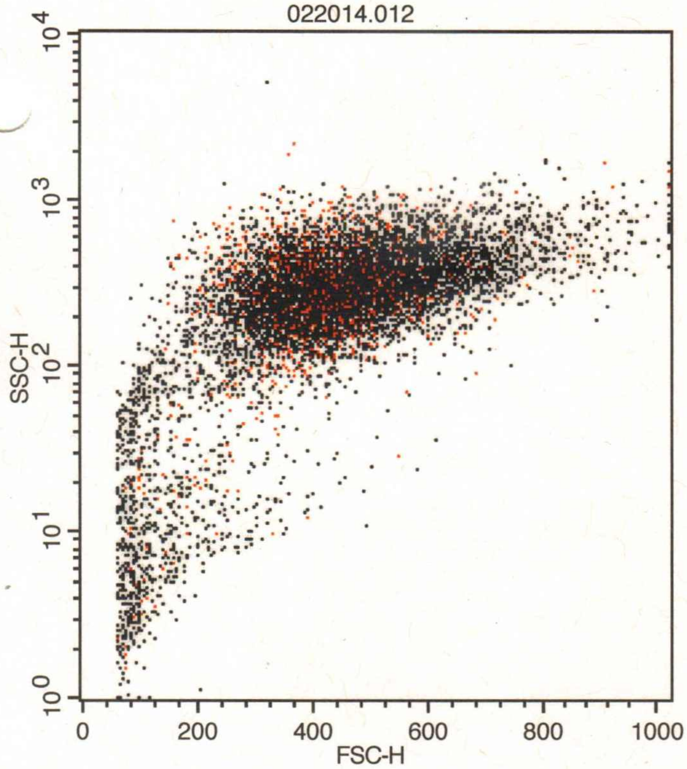

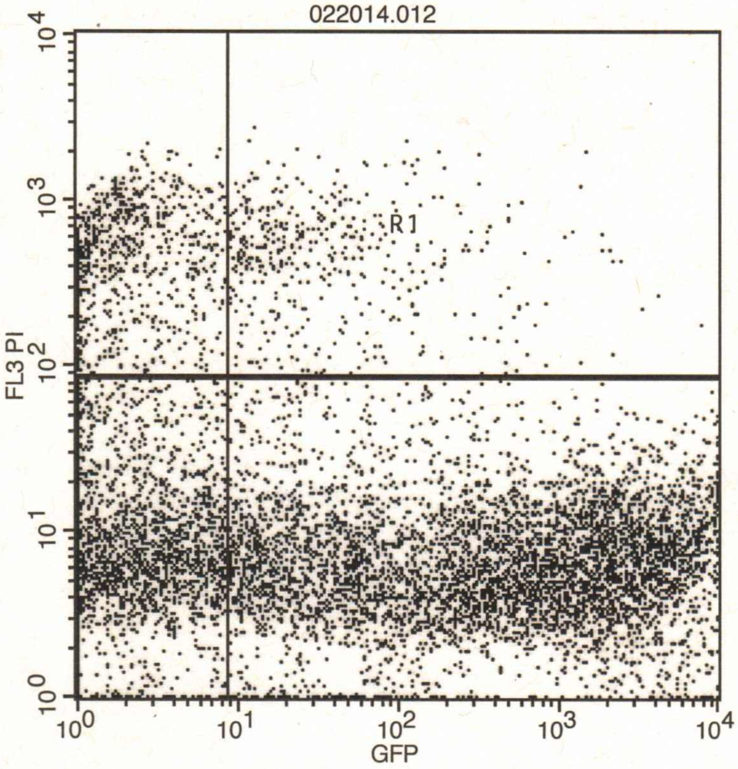

Quadrant Statistics

File: 022014.012
Sample ID:
Tube: Untitled
Acquisition Date: 20-Feb-14
Gated Events: 10000
X Parameter: GFP (Log)
Quad Location: 9, 83

Log Data Units: Linear Values
Patient ID:
Panel: Untitled Acquisition Tube List
Gate: No Gate
Total Events: 10000
Y Parameter: FL3 PI (Log)

Quad	Events	% Gated	% Total	X Mean	X Geo Mean	Y Mean	Y Geo Mean
UL	868	8.68	8.68	2.50	1.98	518.93	395.96
UR	456	4.56	4.56	155.31	39.40	546.10	415.26
LL	3129	31.29	31.29	2.49	1.88	13.33	7.56
LR	5547	55.47	55.47	1516.21	374.39	8.85	6.17

VH14 PEST/GFP

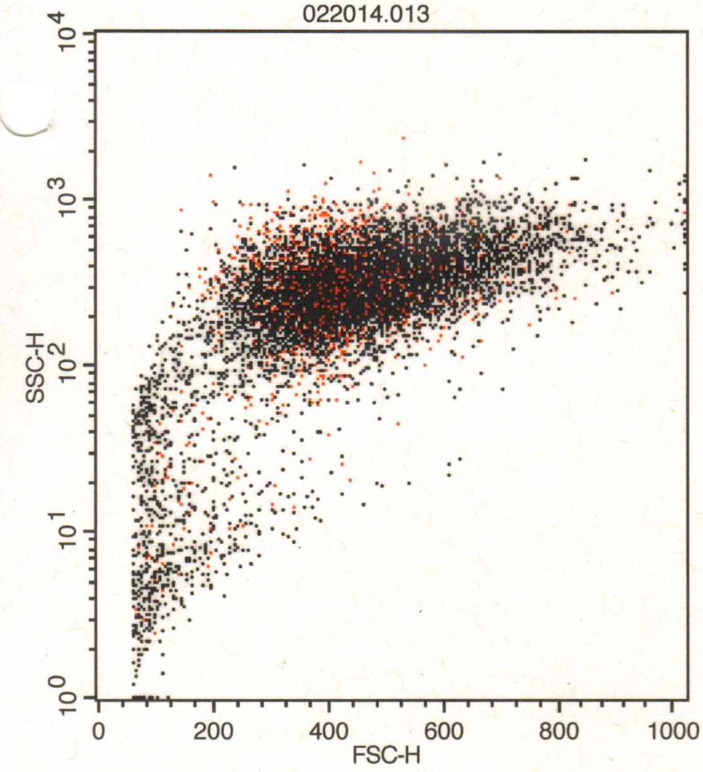

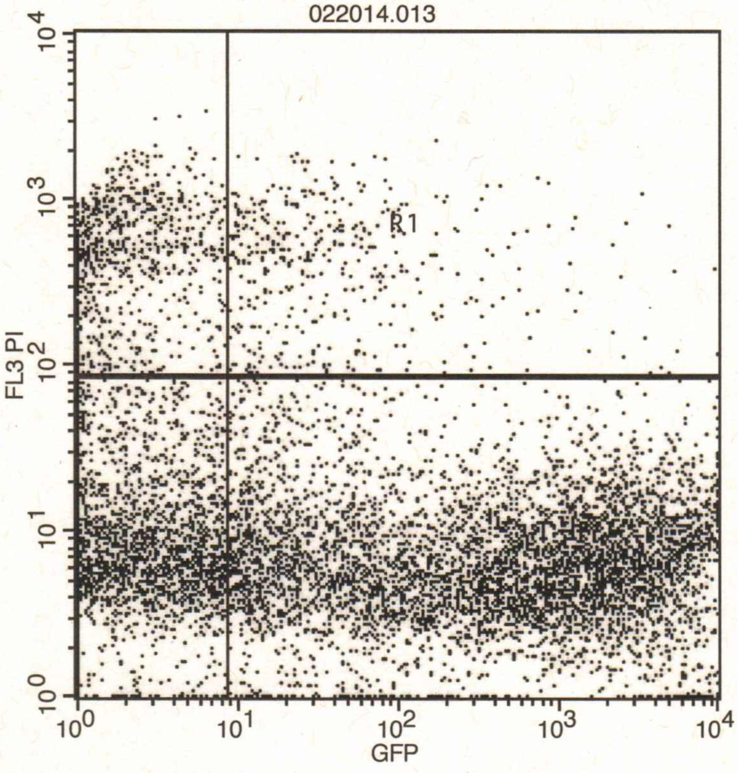

Quadrant Statistics

File: 022014.013
Sample ID:
Tube: Untitled
Acquisition Date: 20-Feb-14
Gated Events: 10000
X Parameter: GFP (Log)
Quad Location: 9, 83

Log Data Units: Linear Values
Patient ID:
Panel: Untitled Acquisition Tube List
Gate: No Gate
Total Events: 10000
Y Parameter: FL3 PI (Log)

Quad	Events	% Gated	% Total	X Mean	X Geo Mean	Y Mean	Y Geo Mean
UL	930	9.30	9.30	2.34	1.86	525.66	391.77
UR	423	4.23	4.23	254.66	45.24	515.21	381.00
LL	3138	31.38	31.38	2.61	1.96	12.87	7.58
LR	5509	55.09	55.09	1514.60	368.53	8.92	6.21

VHH PEST/GFP

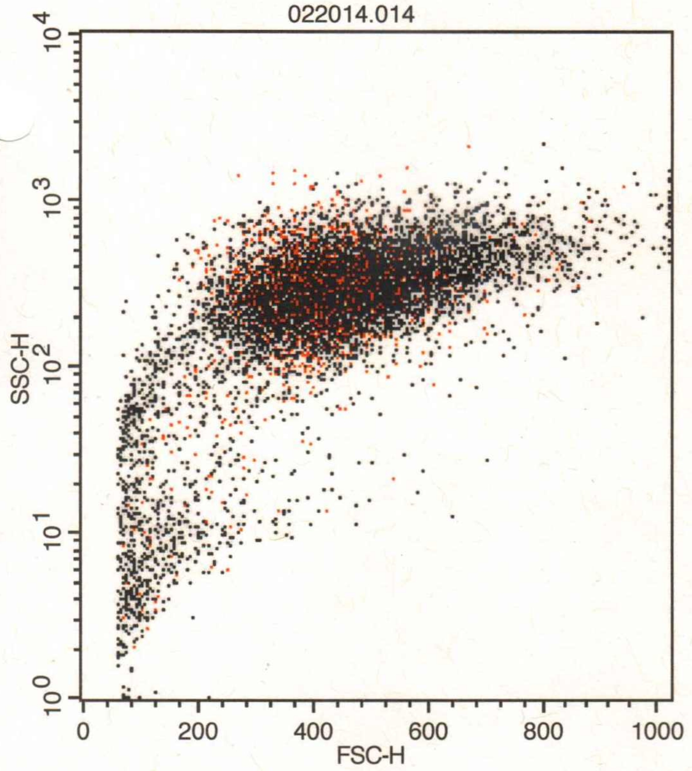

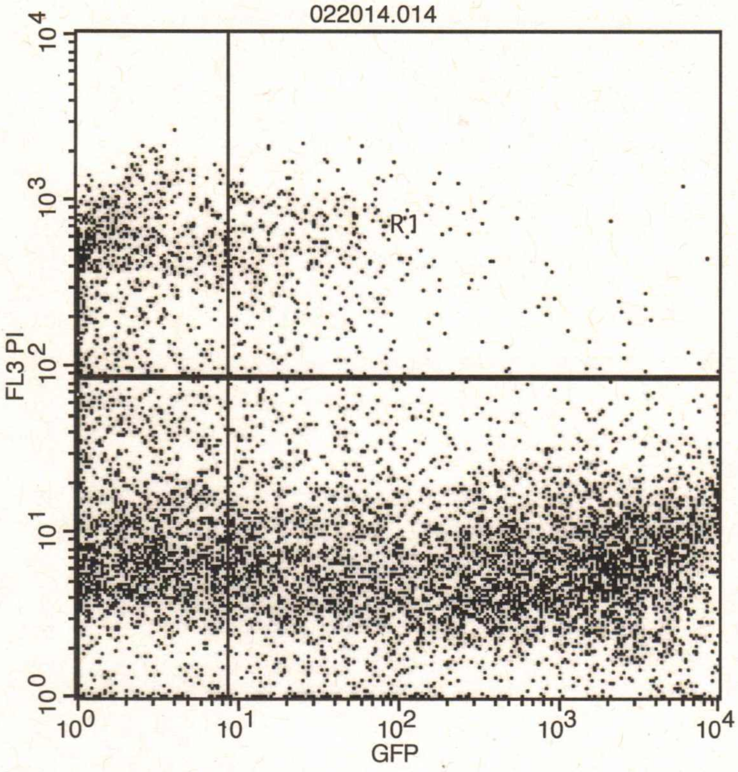

Quadrant Statistics

File: 022014.014
Sample ID:
Tube: Untitled
Acquisition Date: 20-Feb-14
Gated Events: 10000
X Parameter: GFP (Log)
Quad Location: 9, 83

Log Data Units: Linear Values
Patient ID:
Panel: Untitled Acquisition Tube List
Gate: No Gate
Total Events: 10000
Y Parameter: FL3 PI (Log)

Quad	Events	% Gated	% Total	X Mean	X Geo Mean	Y Mean	Y Geo Mean
UL	984	9.84	9.84	2.44	1.92	492.21	370.25
UR	450	4.50	4.50	224.99	37.94	526.21	397.53
LL	3073	30.73	30.73	2.49	1.89	12.82	7.37
LR	5493	54.93	54.93	1534.25	368.50	8.89	6.11

Empty Vector / Syn ~GFP

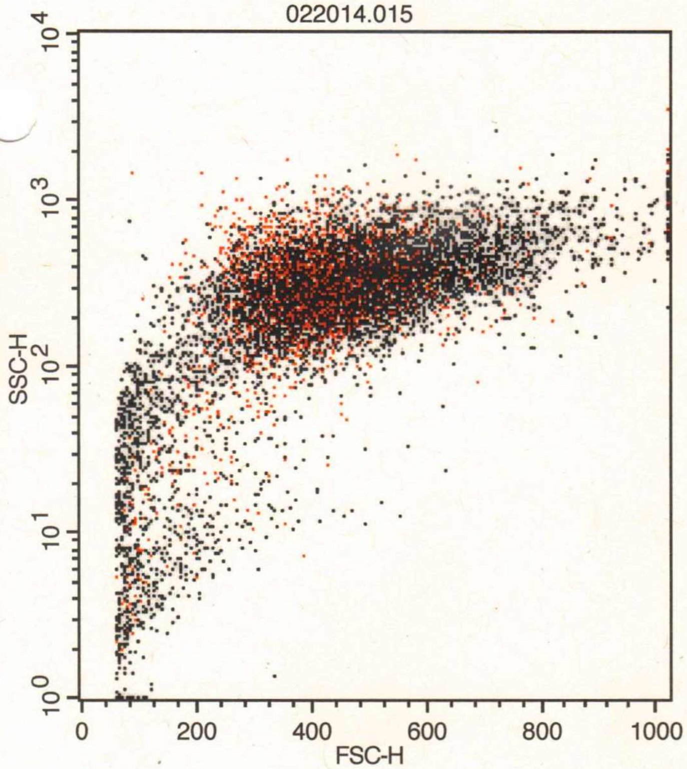

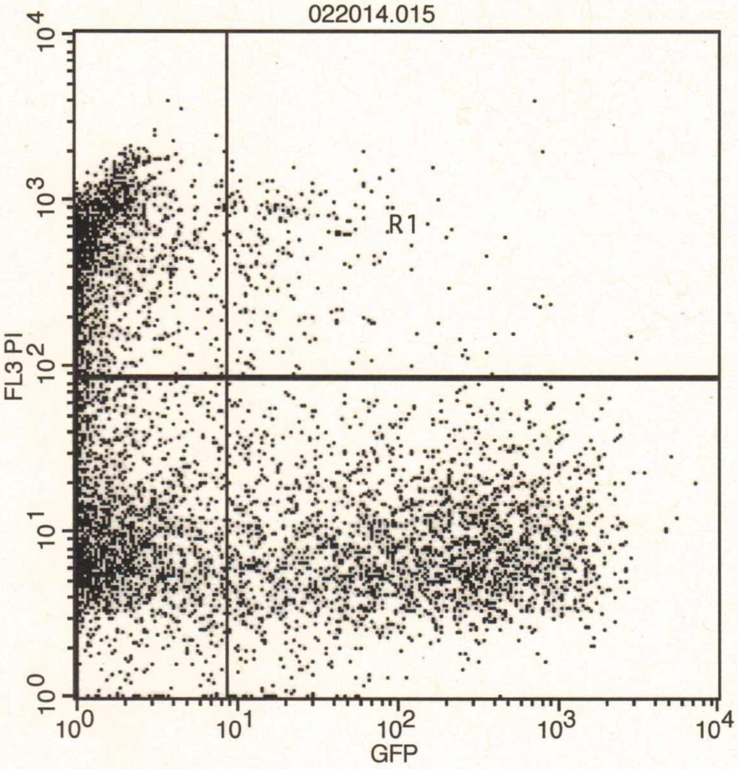

Quadrant Statistics

File: 022014.015
Sample ID:
Tube: Untitled
Acquisition Date: 20-Feb-14
Gated Events: 10000
X Parameter: GFP (Log)
Quad Location: 9, 83

Log Data Units: Linear Values
Patient ID:
Panel: Untitled Acquisition Tube List
Gate: No Gate
Total Events: 10000
Y Parameter: FL3 PI (Log)

Quad	Events	% Gated	% Total	X Mean	X Geo Mean	Y Mean	Y Geo Mean
UL	1993	19.93	19.93	1.67	1.44	534.70	395.97
UR	235	2.35	2.35	97.10	32.32	568.91	417.25
LL	5326	53.26	53.26	1.54	1.30	14.37	8.38
LR	2446	24.46	24.46	362.99	143.83	11.46	8.03

Empty Vector / Syn GFP

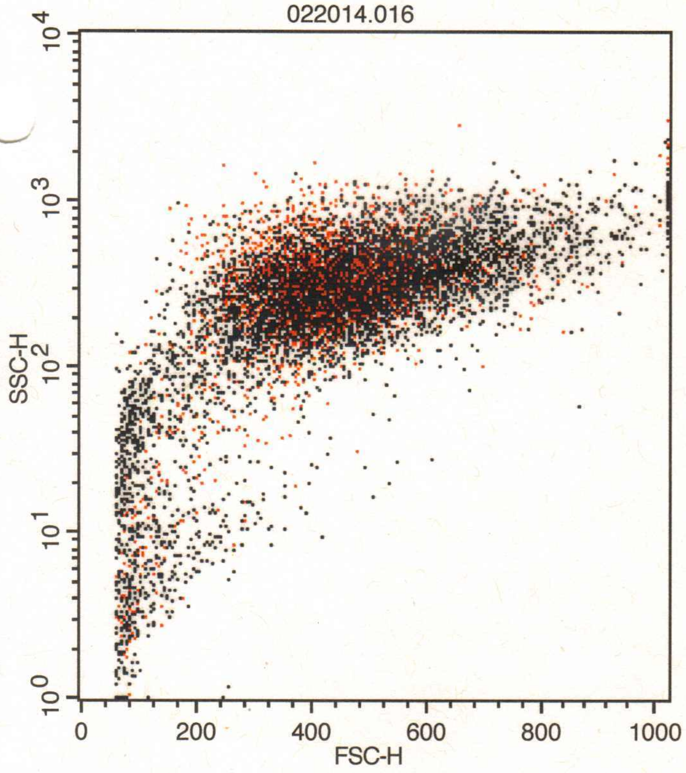

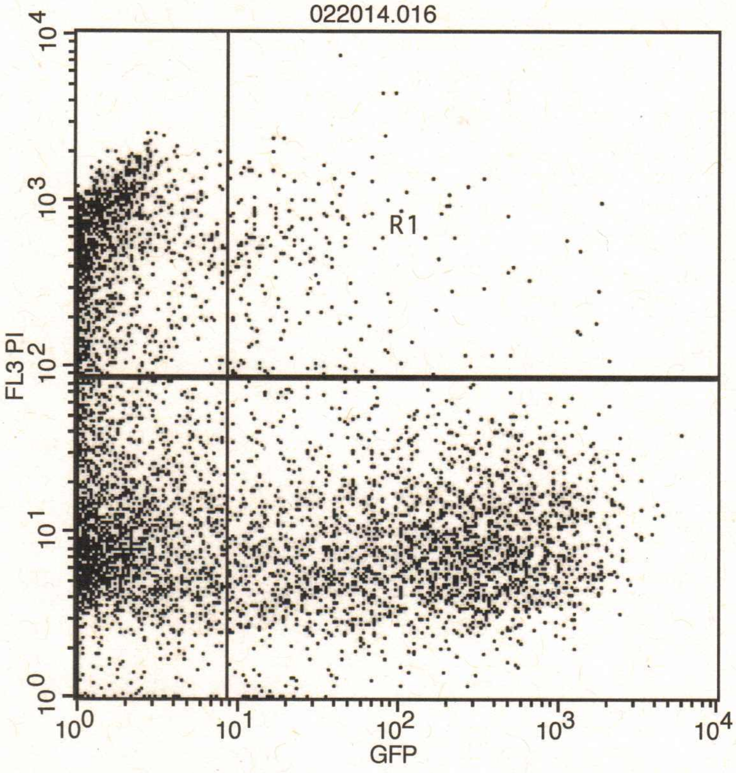

Quadrant Statistics

File: 022014.016
Sample ID:
Tube: Untitled
Acquisition Date: 20-Feb-14
Gated Events: 10000
X Parameter: GFP (Log)
Quad Location: 9, 83

Log Data Units: Linear Values
Patient ID:
Panel: Untitled Acquisition Tube List
Gate: No Gate
Total Events: 10000
Y Parameter: FL3 PI (Log)

Quad	Events	% Gated	% Total	X Mean	X Geo Mean	Y Mean	Y Geo Mean
UL	1930	19.30	19.30	1.65	1.43	542.21	399.18
UR	224	2.24	2.24	113.72	33.51	629.93	410.67
LL	5439	54.39	54.39	1.57	1.32	14.36	8.70
LR	2407	24.07	24.07	366.15	150.67	11.26	8.00

Empty Vector / Syn~GFP

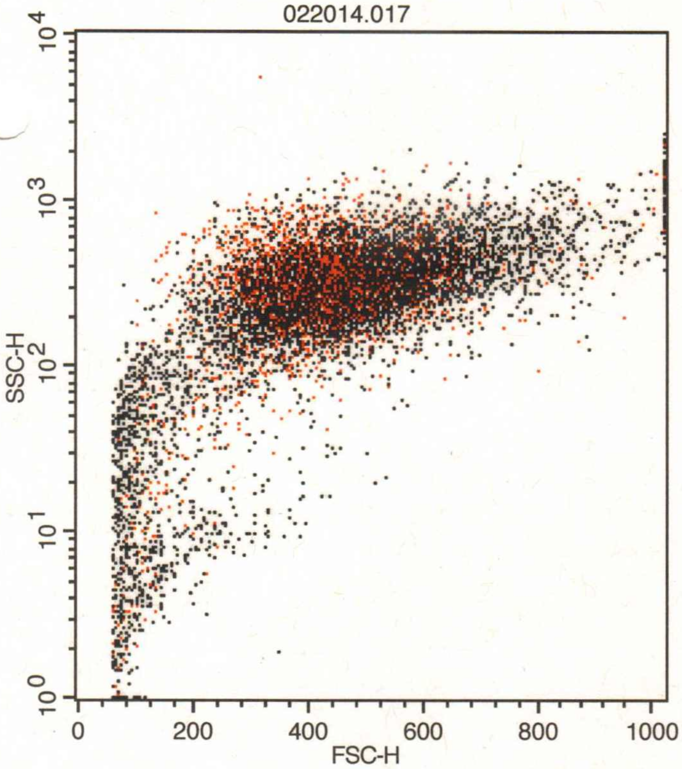

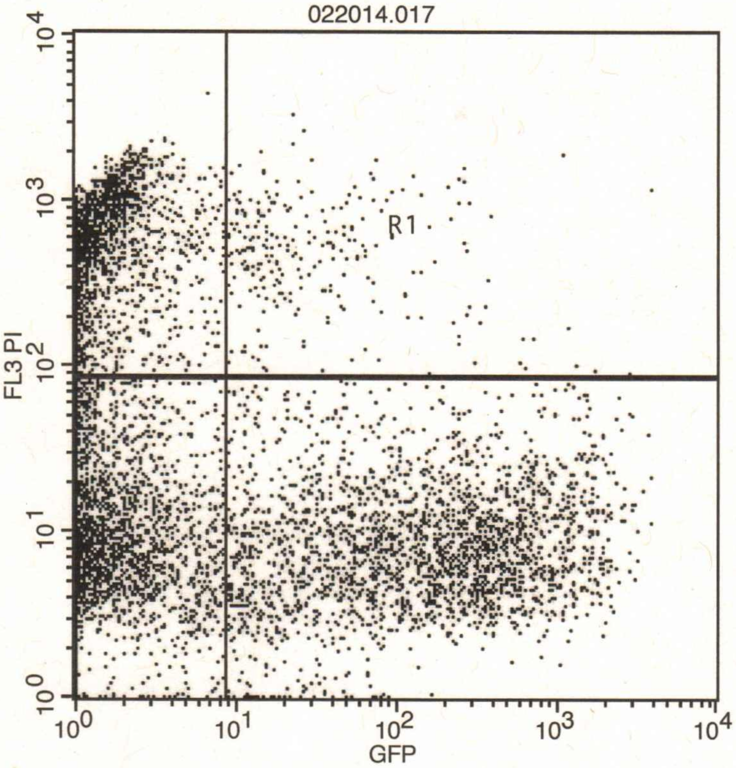

Quadrant Statistics

File: 022014.017
Sample ID:
Tube: Untitled
Acquisition Date: 20-Feb-14
Gated Events: 10000
X Parameter: GFP (Log)
Quad Location: 9, 83

Log Data Units: Linear Values
Patient ID:
Panel: Untitled Acquisition Tube List
Gate: No Gate
Total Events: 10000
Y Parameter: FL3 PI (Log)

Quad	Events	% Gated	% Total	X Mean	X Geo Mean	Y Mean	Y Geo Mean
UL	2138	21.38	21.38	1.71	1.47	565.20	416.85
UR	256	2.56	2.56	110.65	33.66	577.59	436.21
LL	5248	52.48	52.48	1.54	1.30	14.56	8.67
LR	2358	23.58	23.58	358.70	136.78	11.75	8.08

Syn 87PEST / Syn~GFP

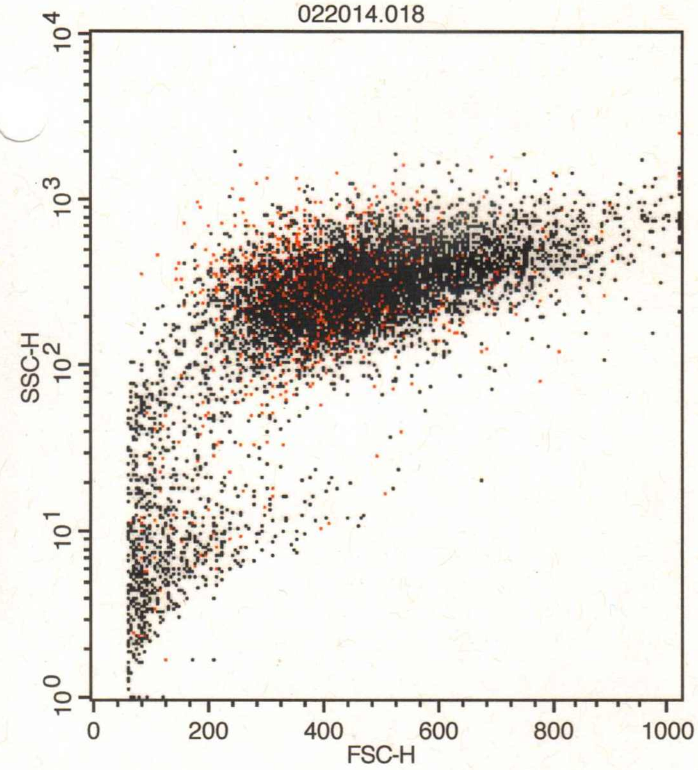

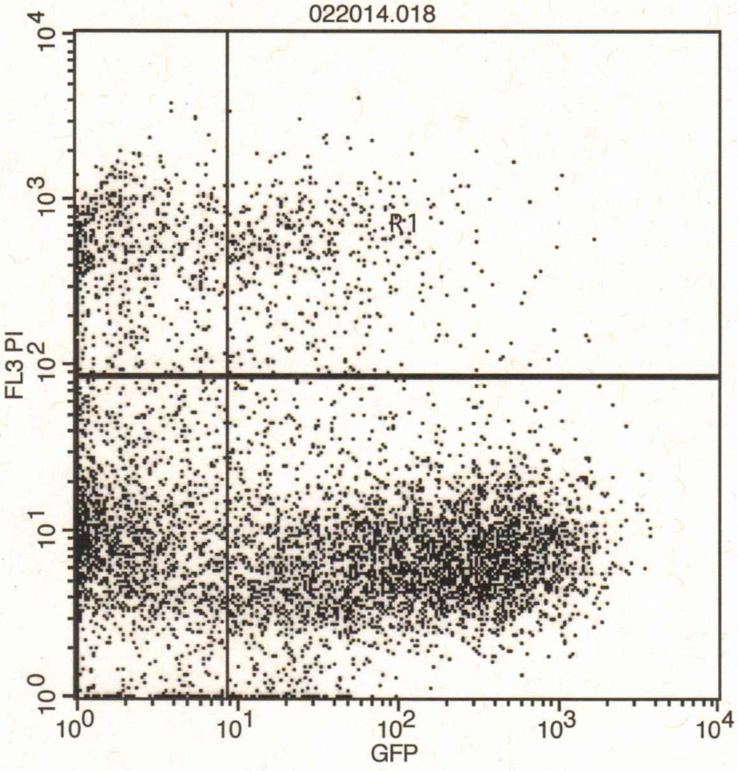

Quadrant Statistics

File: 022014.018
Sample ID:
Tube: Untitled
Acquisition Date: 20-Feb-14
Gated Events: 10000
X Parameter: GFP (Log)
Quad Location: 9, 83

Log Data Units: Linear Values
Patient ID:
Panel: Untitled Acquisition Tube List
Gate: No Gate
Total Events: 10000
Y Parameter: FL3 PI (Log)

Quad	Events	% Gated	% Total	X Mean	X Geo Mean	Y Mean	Y Geo Mean
UL	844	8.44	8.44	2.35	1.84	511.63	376.88
UR	464	4.64	4.64	85.70	36.87	579.49	429.23
LL	4573	45.73	45.73	1.70	1.37	11.35	7.45
LR	4119	41.19	41.19	294.64	128.76	9.06	6.70

Sp 87PEST / Sp ~GFP

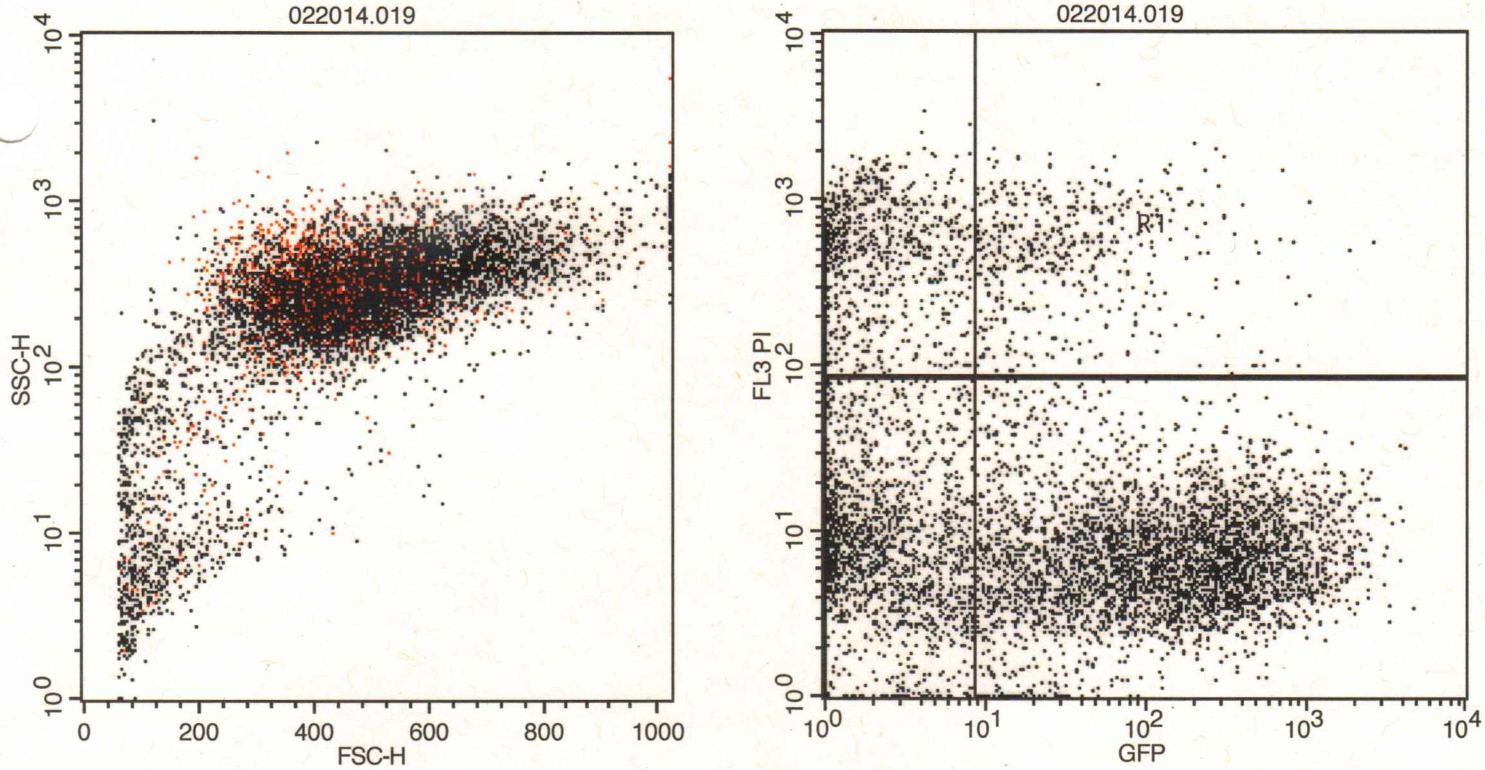

Quadrant Statistics

File: 022014.019
Sample ID:
Tube: Untitled
Acquisition Date: 20-Feb-14
Gated Events: 10000
X Parameter: GFP (Log)
Quad Location: 9, 83

Log Data Units: Linear Values
Patient ID:
Panel: Untitled Acquisition Tube List
Gate: No Gate
Total Events: 10000
Y Parameter: FL3 PI (Log)

Quad	Events	% Gated	% Total	X Mean	X Geo Mean	Y Mean	Y Geo Mean
UL	962	9.62	9.62	2.27	1.82	531.22	401.62
UR	480	4.80	4.80	83.13	34.97	590.80	458.26
LL	4610	46.10	46.10	1.73	1.37	11.51	7.42
LR	3948	39.48	39.48	296.40	126.58	9.18	6.70

Syn 87PEST / Syn~GFP

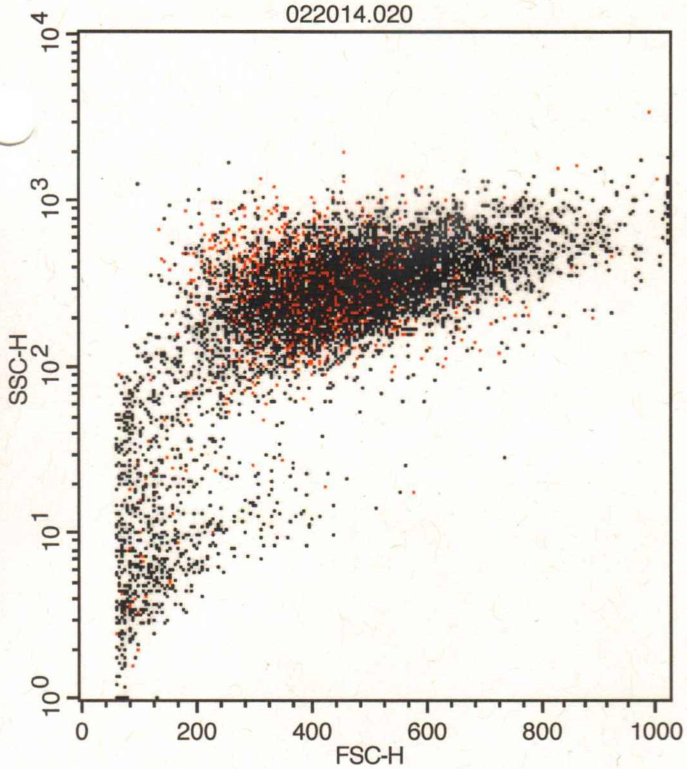

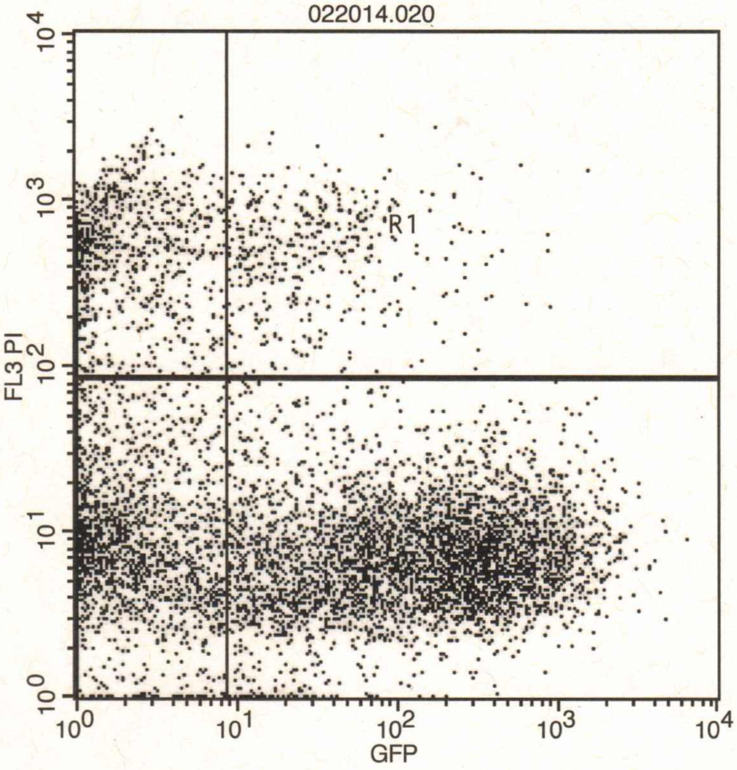

Quadrant Statistics

File: 022014.020
Sample ID:
Tube: Untitled
Acquisition Date: 20-Feb-14
Gated Events: 10000
X Parameter: GFP (Log)
Quad Location: 9, 83

Log Data Units: Linear Values
Patient ID:
Panel: Untitled Acquisition Tube List
Gate: No Gate
Total Events: 10000
Y Parameter: FL3 PI (Log)

Quad	Events	% Gated	% Total	X Mean	X Geo Mean	Y Mean	Y Geo Mean
UL	933	9.33	9.33	2.30	1.83	538.49	397.14
UR	418	4.18	4.18	63.19	32.20	583.71	457.84
LL	4614	46.14	46.14	1.70	1.36	11.46	7.47
LR	4035	40.35	40.35	315.09	132.20	9.25	6.84

VHH PEST / Syn-GFP

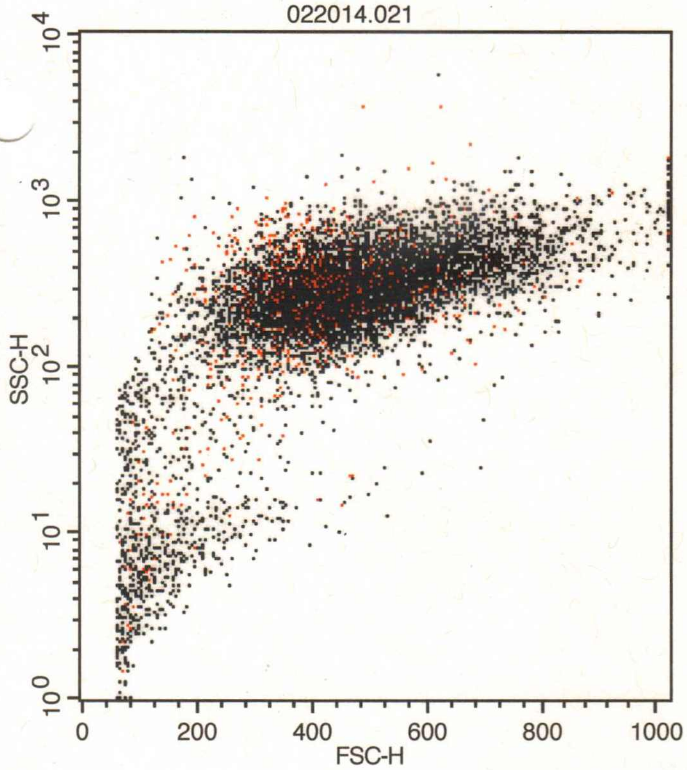

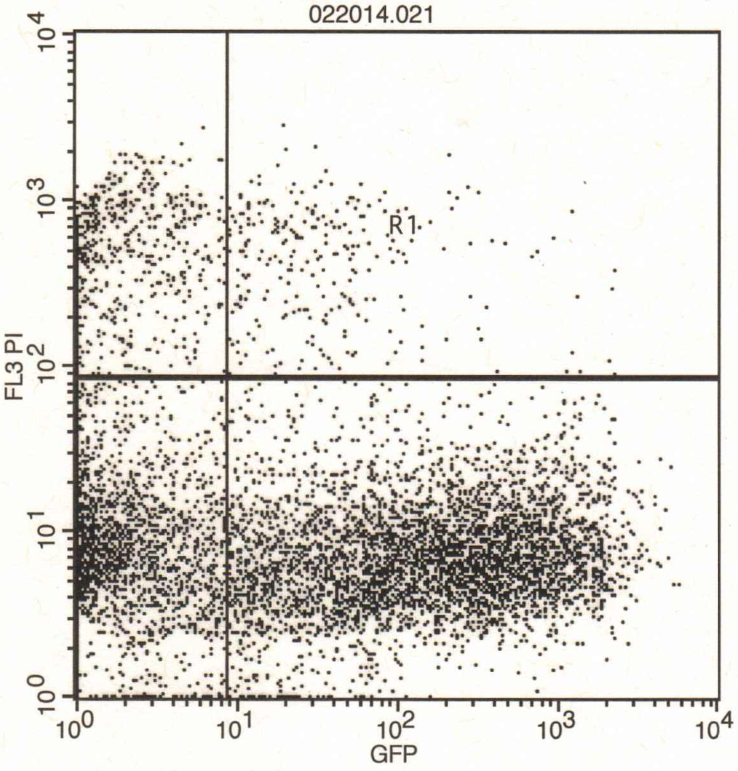

Quadrant Statistics

File: 022014.021
Sample ID:
Tube: Untitled
Acquisition Date: 20-Feb-14
Gated Events: 10000
X Parameter: GFP (Log)
Quad Location: 9, 83

Log Data Units: Linear Values
Patient ID:
Panel: Untitled Acquisition Tube List
Gate: No Gate
Total Events: 10000
Y Parameter: FL3 PI (Log)

Quad	Events	% Gated	% Total	X Mean	X Geo Mean	Y Mean	Y Geo Mean
UL	730	7.30	7.30	2.12	1.69	515.10	385.81
UR	268	2.68	2.68	115.92	36.72	527.90	394.24
LL	4785	47.85	47.85	1.67	1.36	10.46	7.11
LR	4217	42.17	42.17	395.45	154.02	9.23	6.87

VHIMPEST/S77~GFP

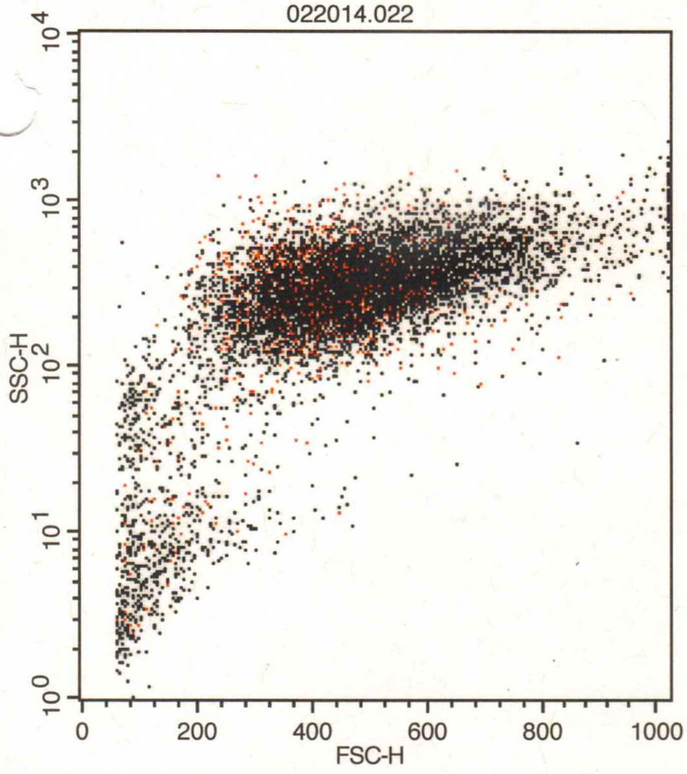

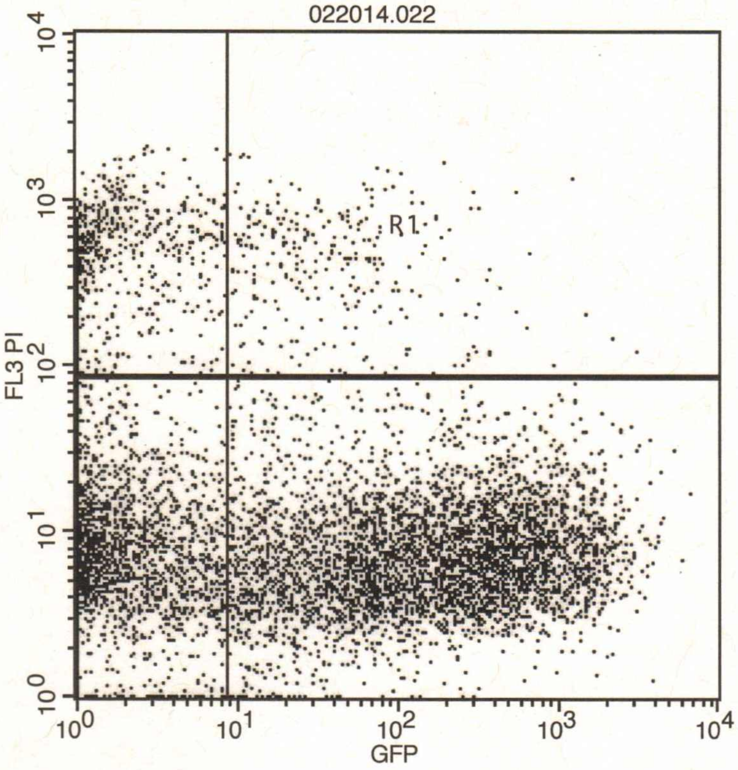

Quadrant Statistics

File: 022014.022
Sample ID:
Tube: Untitled
Acquisition Date: 20-Feb-14
Gated Events: 10000
X Parameter: GFP (Log)
Quad Location: 9, 83

Log Data Units: Linear Values
Patient ID:
Panel: Untitled Acquisition Tube List
Gate: No Gate
Total Events: 10000
Y Parameter: FL3 PI (Log)

Quad	Events	% Gated	% Total	X Mean	X Geo Mean	Y Mean	Y Geo Mean
UL	721	7.21	7.21	2.17	1.70	514.68	399.78
UR	291	2.91	2.91	99.92	40.82	509.89	392.53
LL	4626	46.26	46.26	1.69	1.37	10.37	7.04
LR	4362	43.62	43.62	393.13	154.29	9.36	6.97

VH 14PEST / S77 ~GFP

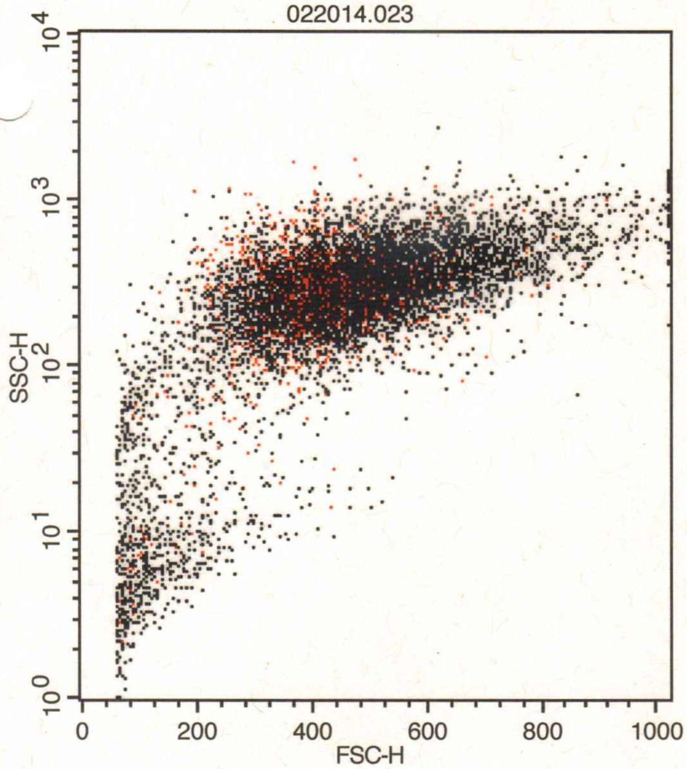

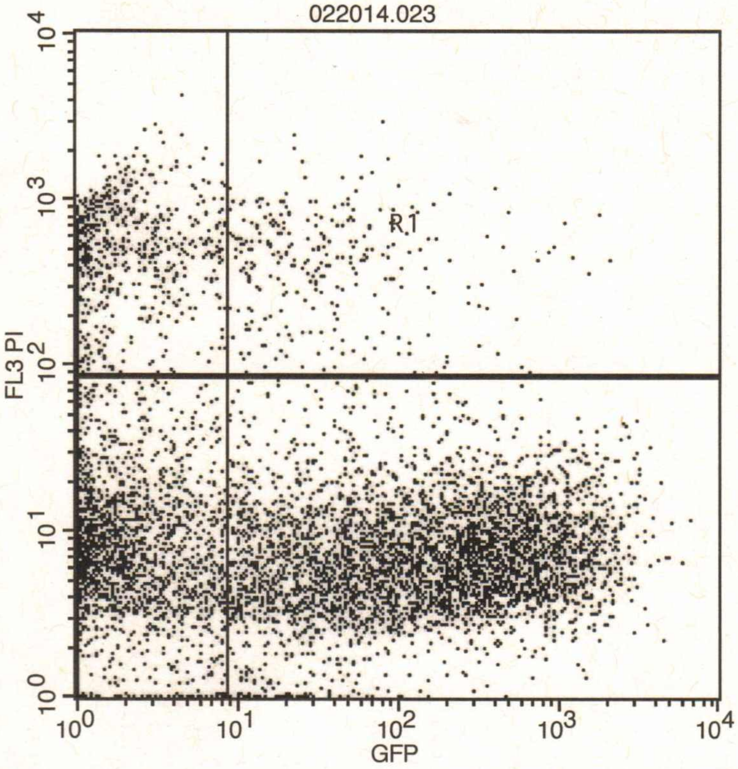

2.15
JH

Quadrant Statistics

File: 022014.023
Sample ID:
Tube: Untitled
Acquisition Date: 20-Feb-14
Gated Events: 10000
X Parameter: GFP (Log)
Quad Location: 9, 83

Log Data Units: Linear Values
Patient ID:
Panel: Untitled Acquisition Tube List
Gate: No Gate
Total Events: 10000
Y Parameter: FL3 PI (Log)

Quad	Events	% Gated	% Total	X Mean	X Geo Mean	Y Mean	Y Geo Mean
UL	820	8.20	8.20	2.23	1.76	524.78	401.03
UR	287	2.87	2.87	99.01	38.14	505.74	391.77
LL	4708	47.08	47.08	1.70	1.37	10.89	7.21
LR	4185	41.85	41.85	392.16	151.36	9.13	6.87
